# Surface lattice engineering for fine-tuned spatial configuration of nanocrystals

**DOI:** 10.1038/s41467-021-25969-7

**Published:** 2021-09-27

**Authors:** Bo Jiang, Yifei Yuan, Wei Wang, Kun He, Chao Zou, Wei Chen, Yun Yang, Shun Wang, Vitaliy Yurkiv, Jun Lu

**Affiliations:** 1 Nanomaterials and Chemistry Key Laboratory, Wenzhou University, Wenzhou, China; 2 Chemical Sciences and Engineering Division, Argonne National Laboratory, Lemont, IL USA; 3 Department of chemistry and Center for pharmacy, University of Bergen, Bergen, Norway; 4 Department of Mechanical and Industrial Engineering, University of Illinois at Chicago, Chicago, IL USA

**Keywords:** Nanoparticles, Synthesis and processing

## Abstract

Hybrid nanocrystals combining different properties together are important multifunctional materials that underpin further development in catalysis, energy storage, et al., and they are often constructed using heterogeneous seeded growth. Their spatial configuration (shape, composition, and dimension) is primarily determined by the heterogeneous deposition process which depends on the lattice mismatch between deposited material and seed. Precise control of nanocrystals spatial configuration is crucial to applications, but suffers from the limited tunability of lattice mismatch. Here, we demonstrate that surface lattice engineering can be used to break this bottleneck. Surface lattices of various Au nanocrystal seeds are fine-tuned using this strategy regardless of their shape, size, and crystalline structure, creating adjustable lattice mismatch for subsequent growth of other metals; hence, diverse hybrid nanocrystals with fine-tuned spatial configuration can be synthesized. This study may pave a general approach for rationally designing and constructing target nanocrystals including metal, semiconductor, and oxide.

## Abstract (web summary)

The spatial configuration of heterostructured nanocrystals has important roles in their applications, but it is difficult to fine tune. Here the authors report a surface lattice engineering approach that can be used to achieve precise control of geometrical symmetry, spatial composition, and dimension of metal heterostructured nanocrystals.

## Introduction

Hybrid nanocrystals have various applications in many fields, including energy conversion^1–3^, catalysis^4–10^, cancer therapy^11^, and optics^12–15^. Accurate control over their spatial configuration (shape, composition, and dimension) is vital to adjusting their physicochemical properties, and consequently, to their effects on different functions^16–20^. Heterogeneous seeded growth, in which depositing one material on pre-made seeds composed of chemically different material, is the most widely used technology to construct these hybrid nanocrystals; the lattice mismatch of material pair strongly governs growth process and final spatial configuration^21–26^. Growth on a highly lattice-mismatched surface can increase system energy due to strain accumulation, and it thus prefers to grow only on one site, leading to low-symmetry Janus-like product with a small heterogeneous interface, for examples, Cu–Au and Cu–Pd material pairs^27–32^. In the case of slight lattice mismatch, homogeneous high-symmetry growth surrounding seeds is thermodynamically favored^33–39^, creating core-shell nanocrystals, for example, Au–Ag material pair^34^. Surely, achieving fine control over lattice mismatch can enhance our ability to tune nanocrystals spatial configuration. Currently, altering material pair is the only way to change lattice mismatch; yet, it is still impossible to fine tune lattice mismatch, since the lattice parameters of each material are fixed value and their difference is discontinuous. Consequently, the ability to precisely control the ultimate spatial configuration of nanocrystals is largely limited. Breaking this limitation will naturally make a path to unexplored construction of hybrid nanocrystals with fine-tuned spatial configuration.

In this work, we report a surface lattice engineering strategy, in which lattice mismatch is tuned by coating one material on seed surface instead of using different types of material pairs; moreover, the fine-tuning of lattice mismatch can be easily realized by controlling the amount of coated material, thus largely enhancing the ability to control the spatial configuration of nanocrystals. To prove that this approach has general applicability but not depends on shape, size, and crystalline structure of seeds, various Au nanocrystals including penta-fold twinned (PFT) nanodecahedrons (AuNDs), nanorods (AuNRs), nanobipyramids (AuNBs), and single crystalline nanoplates (AuNPs) and truncated octahedrons (AuTNOs) are chosen as seeds in this study, for their preparations can be realized using existing technologies^40–46^. Pt, which has a lattice mismatch larger than 3% relative to Au, can largely influence the heterogeneous growth of Au^47–50^. Besides, hybrid nanocrystals composed of Pt and Au exhibit enhanced catalytic performance and multifunction owing to synergistic effects^51–54^, and thus we employed Pt as the lattice modifier. Different amount of Pt is coated on Au seeds surfaces, which generates adjustable lattice mismatch for subsequent growth of various metals (Au, Ag, and Pd) and consequently allows the formation of hybrid nanocrystals with fine-tuned spatial configuration. This approach is independent of the structure, shape and size of Au seeds. In addition, since lattice mismatch is universal in all crystal systems^39^, this strategy may pave a way toward the advancement of various hybrid nanocrystals with deterministically targeted spatial configuration.

## Results

### Density function theory (DFT) study

**Fig. 1:**
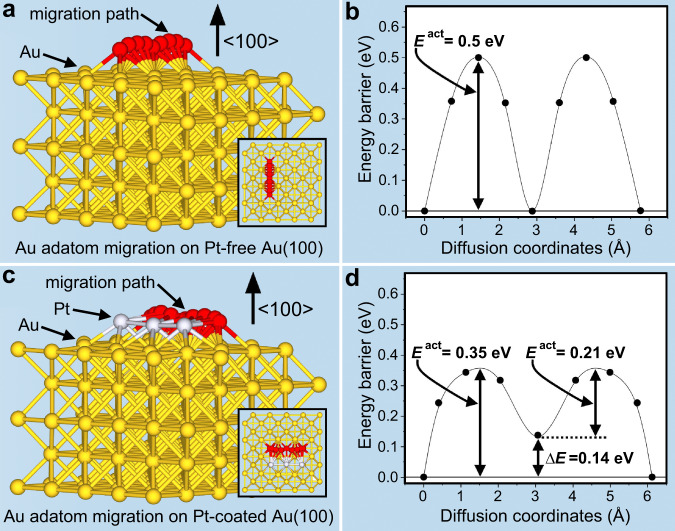
DFT study and schematic adatom migration. **a**, **b** Au adatom migration on Pt-free Au(100) surface, **c**, **d** Au adatom migration on Pt-coated Au(100). The insets in **a** and **c** are top view of adsorbate structure.

To evaluate the effect of Pt coating on subsequent growth of other metals, Au was taken as an example to perform DFT calculation. Similar to a common seed-mediated process, Au growth includes three stages: (1) formation of nucleation sites, (2) absorption of Au atoms on seed surface (the formation of Au adatoms), and (3) migration of adatoms to nucleation site to deposit^36^. The last stage essentially determines final spatial configuration of product. The Au nanocrystals (AuND, AuNR, AuNB, AuTNO) used in this work are bounded by {111} and {100} facets or stepped {100} facets, which are energetically inactive surfaces with the energy of {100} facet slightly higher than that of {111} facet^55^. Considering that Pt deposition preferentially occurs on high-energy positions, the effect of Pt on the migration of Au adatom on {100} was investigated. Result shows that the activation energy (*E*^*ac*t^) of Au-on-Au(100) migration is 0.5 eV (Fig. 1a, b). When neighboring Pt atoms are present, the *E*^*ac*t^ significantly decreases to 0.35 eV (Fig. 1c, d). Pt can increase Au diffusion coefficient by three orders of magnitude from 5.92 × 10^−15^ m^2^ s^−1^ to 3.0 × 10^−12^ m^2^ s^−1^, implying that the presence of Pt atoms leads to a high migration rate of Au adatoms. Wolf et al. draw a similar conclusion that negative strain (compressive) and positive strain (tensile) can speed up and slow down atomic diffusion, respectively^56^. Au (4.078 Å) has larger cell parameter than Pt (3.924 Å)^57^, so the migration of Au adatom on Pt-doped or Pt-coated {100} should follow the negative strain pattern. Therefore, it is theoretically feasible to tune adatom migration and control growth pattern via surface lattice engineering.

### Surface lattice engineering on AuNDs via Pt deposition

**Fig. 2:**
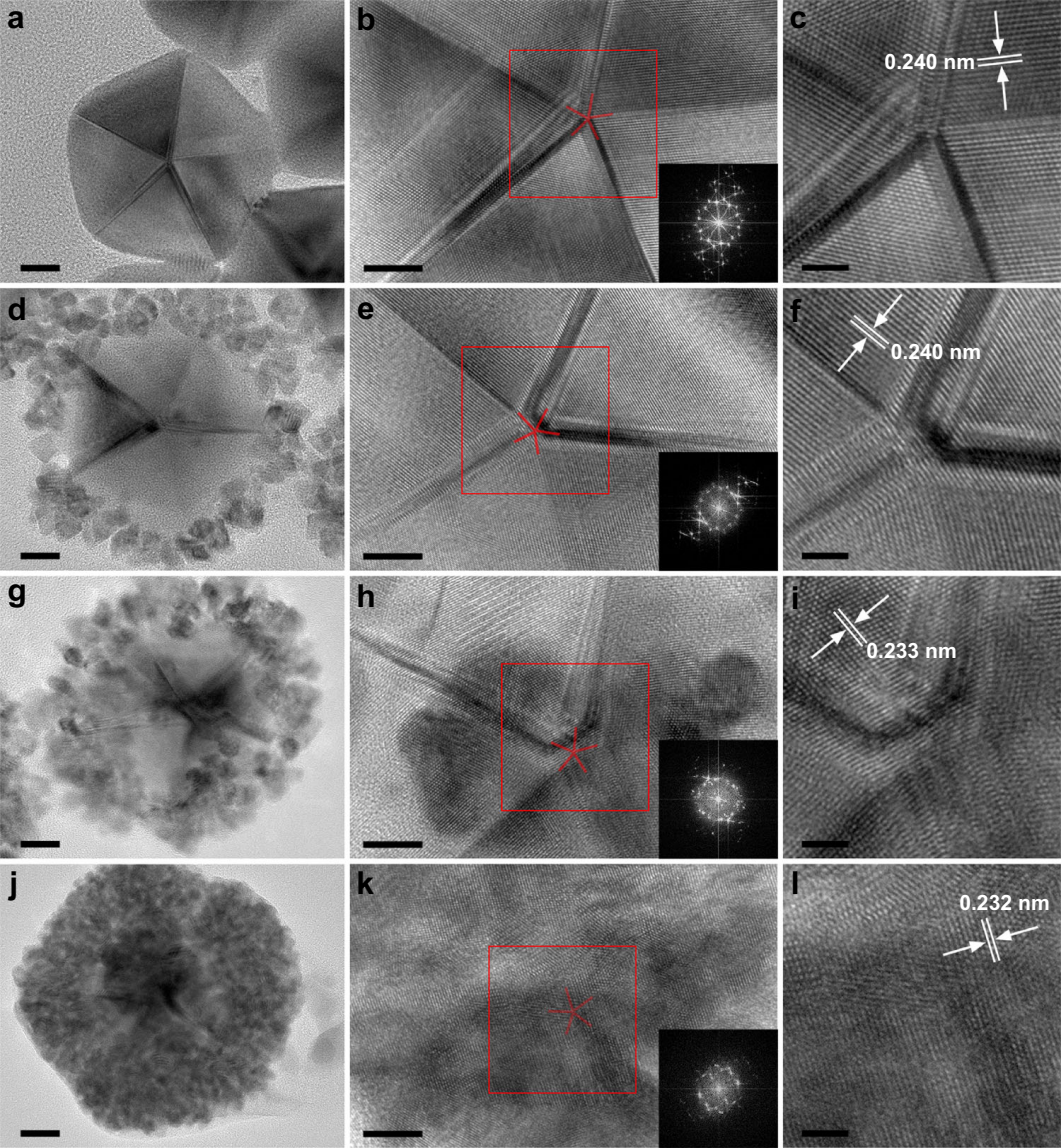
Surface lattice engineering on AuNDs surface. HRTEM images of AuND@PtNC nanocrystals with different molar ratios of Pt and Au: **a**, **b**, **c** 0; **d**, **e**, **f** 0.5; **g**, **h**, **i** 1; **j**, **k**, **l** 2. The images in **c**, **f**, **i**, **l** are the areas marked by the red boxes in **b**, **e**, **h**, **k** and the insets are corresponding FFT patterns. Scale bars in **a**, **d**, **g**, **j** are 10 nm. Scale bars in **b**, **e**, **h**, **k** are 5 nm. Scale bars in **c**, **f**, **i**, **l** are 2 nm.

Inspired by the DFT results, surface lattice engineering was first applied to PFT AuNDs. High resolution transmitted electron microscopy (HRTEM) was used to investigate how surface lattice feature changes with Pt content (Fig. 2). For Pt-free AuNDs, PFT structure was clearly observed (Fig. 2a–c and Supplementary Fig. 2). HRTEM image shows a 0.240 nm inter-fringe distance which is attributed to the lattice spacing of Au(111) plane^58^. After introducing small amount of Pt (Pt/Au = 0.5:1 mol/mol), Pt atoms were selectively deposited on the {100} facets and adjacent {111} facets of AuNDs to form Pt nanocircles (PtNCs) (Fig. 2d–f and Supplementary Figs. 1b, 3–5), producing AuND@PtNC nanocrystals^59^. PFT feature on most of {111} facets was not strongly influenced. The lattice spacing of Au(111) plane near penta-fold axis has no changes, indicating that no Pt atoms were deposited here. However, the areas near five edges became rough because of preferential island-like deposition of Pt atoms. When the molar ratio of Pt and Au was increased to 1, surface crystalline feature significantly changed (Fig. 2g–i and Supplementary Figs. 1c, 6–8). Although the Pt growth on {100} facet was still dominant, a considerable amount of Pt atoms were also deposited on {111} facets, especially on the tip, as demonstrated by scanning transmitted electron microscopy (STEM) high angle annular dark field (HAADF) images and STEM energy dispersive X-ray spectroscopy (EDS) elemental maps. The lattice spacing of (111) plane, as a result of Pt doping, decreases to 0.233 nm. The Pt deposition near the tip is closely related to reaction kinetics. When more Pt precursors are added into the reaction system, Pt atoms in solution increases, placing a high demand for nucleation sites. The surface atoms on the {100} facets of AuNDs and adjacent positions were unable to provide enough nucleation sites, so nucleation occurred on other positions. The atoms on sharp tip are always active, which thus promotes Pt growth in these positions^52^. When the concentration of Pt precursor was further increased (Pt/Au = 2:1 mol/mol) (Fig. 2j–l and Supplementary Figs. 1d, 9), the fast growth kinetics of Pt forced almost all surface Au atoms of {111} facets to act as nucleation sites and AuNDs were fully coated by Pt. Therefore, it is difficult to recognize the PFT structure using HRTEM. The lattice spacing of (111) plane is 0.232 nm, which is smaller and closer to that of Pt(111) compared with former case. The fast Fourier transform patterns (FFT) also gave the same conclusion that Pt deposition makes the surface crystalline feature deviate from PFT structure. Clearly, surface crystalline feature of AuND can be engineered in two respects. (1) Controlled deviation from PFT structure. The crystalline feature change is attributed to the island-like deposition of small Pt particles, which formed many twinned boundaries and dislocations through ripening (Supplementary Fig. 7); therefore, the surface crystalline feature of AuND is deviated from PFT structure. (2) Fine tuning of lattice spacing. The lattice spacing of Pt(111) plane and Au(111) plane are about 0.230 nm and 0.240 nm, respectively. The inter-diffusion of Pt coating and Au substrate results in formation Pt/Au alloy^36^, especially on the AuND surface. Therefore, lattice spacing reduces with Pt content.

### AuND-derived nanocrystals with controlled geometrical symmetry

**Fig. 3:**
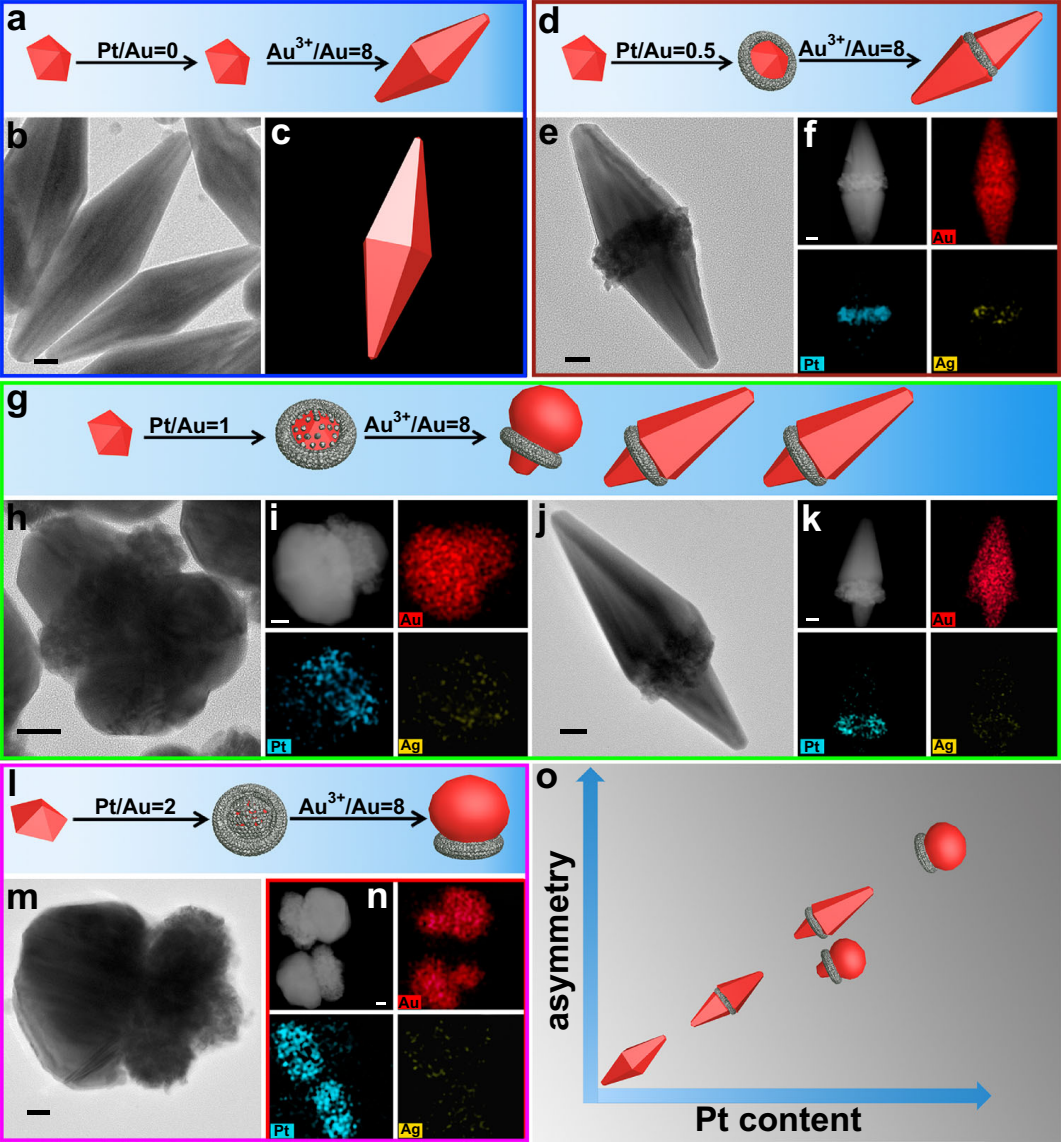
Au growths on AuND-based nanocrystals. TEM images, STEM-HAADF images, STEM-EDS elemental maps, and schematic illustrations of products prepared through growing Au on AuND@PtNC nanocrystals with different molar ratios of Pt/Au: **a**, **b**, **c** 0; **d**, **e**, **f** 0.5; **g**, **h**, **i**, **j**, **k** 1; **l**, **m**, **n** 2. **o** Schematic evolution of spatial configuration with Pt. All scale bars are 20 nm. In all schematics, the red and gray colors represent Au and Pt, respectively.

Above results show that surface lattice engineering provides access to the fine tuning of crystalline structure and lattice spacing on AuND surface. For subsequent Au growths on these AuND@PtNC nanocrystals, their patterns should be related with Pt content because Pt-dependent lattice spacing brings adjustable lattice mismatch, which was confirmed by the following results. Pt-free AuNDs induced the formation of AuNBs which are almost symmetrical along penta-fold axis (Fig. 3a–c and Supplementary Fig. 10a, b)^58^. When Pt-coated AuNDs (the Pt/Au molar ratio was 0.5) served as seeds, Au growth produced an interesting structure, in which each AuNB is coated by a PtNC (AuND@PtNC) (Fig. 3d–f and Supplementary Figs. 10e, f). The PtNCs are very useful markers for identifying the growth preference of Au. The average lengths from the PtNC to one end are 150 nm and 90 nm to another end (Supplementary Fig. 11), suggesting that the growths along two directions are not equal; that is, the AuNB sections of AuND@PtNC nanocrystals are asymmetric along longitudinal direction. HRTEM images and FFT pattern show that the AuNB section in AuNB@PtNC has the same PFT structure as common AuNB (Supplementary Fig. 12h–m), suggesting that small amount of Pt has no effect on the crystalline feature of products. Once the molar ratio of Pt and Au in seeds was increased to 1, almost half of products are still AuNB@PtNC nanocrystals, but Au growths created irregular Au domains in the rest (Fig. 3g–k and Supplementary Fig. 10i, j). Both types of nanocrystals have lower longitudinal symmetry than the AuNB@PtNC shown in Fig. 3e; for example in AuNB@PtNC nanocrystals, the average length from the PtNC to one end increased to 160 nm and to another end decreased to 70 nm (Supplementary Fig. 13). Distinctly, high Pt content can further increase the difference of growth rate along two directions; high Pt content also caused large deviation of crystalline feature from PFT on seeds surfaces (Supplementary Figs. 7, 14), and thereby AuNDs lost their guiding effect, producing irregular Au domains. If the molar ratio of Pt and Au in seeds was 2, unidirectional growth occurred and yielded Janus-like (AuND@PtNC)-Au nanocrystals (Fig. 3l–n and Supplementary Figs. 10m, n, 15, 16), indicating that growth symmetry was further decreased. Meanwhile, almost all seeds surfaces lost PFT crystalline feature due to thick and dense Pt layer, which made each formed Au domain become irregular. The above results clearly demonstrate that nanocrystals spatial configuration can be controlled via this surface lattice engineering (Fig. 3o).

### Growth mechanism

As discussed previously, the seeded growth process generally includes the formation of nucleation sites on seed surface, atom adsorption, and adatom migration to nucleation site and growth^36^. In our experiments, the formation of asymmetrical AuNB@PtNC nanocrystals demonstrates that preferential migration to one side occurred. Currently, it remains a grand challenge to in situ observe how Pt influences the product’s asymmetry. Here, a mechanism is provided to explain the Pt-dependent growth by using the AuNB@PtNC nanocrystal as an example.

**Fig. 4:**
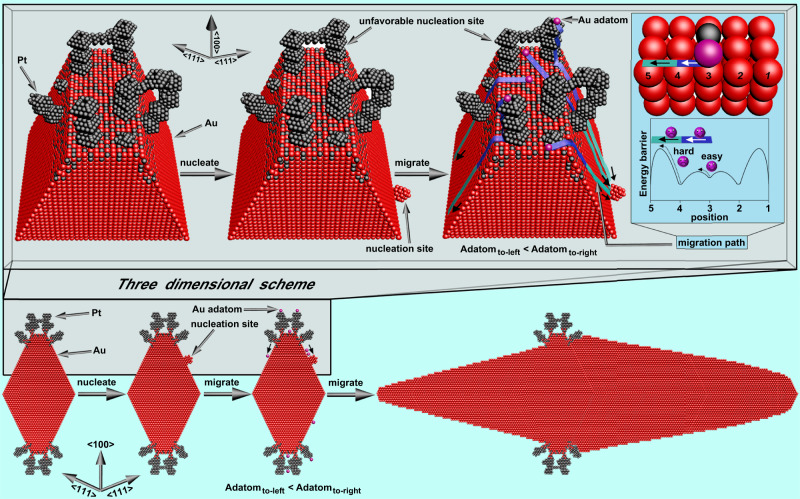
Pt-dependent asymmetry. Schematic of growing asymmetrical AuNB@PtNC.

First, one could reasonably hypothesize that the first nucleation site appearing on the surface of Au seeds may be randomly distributed on either side of the crystal but not in the Pt-coated edge region since the higher degree of lattice mismatch in the Pt coated region can prevent any Au nucleation^60^. As shown in Fig. 4, when a higher concentration of Au atoms create the firstly emerging nucleation site on one side of the AuND (supposed to be the right side as illustrated in the schematic), other Au adatoms would prefer to migrate to this active position to supply the growth of Au on this side because the nucleation site rich in atomic steps and defects is very active^61^. With the increase of synthesis time, the initially symmetric morphology of the Au seeds is gradually broken along longitudinal axis.

Second, it is certain that Pt coating can further increase the degree of asymmetry by facilitating the migration of Au adatoms from these Pt-coated surfaces to the nucleation sites to boost the growth of Au. For the used AuND nanocrystals, their five identical edges, in practice, are small-area {100} facets but not lines in theory (Supplementary Fig. 2), indicating the selective coating of Pt on {100} facets. As demonstrated by the DFT calculations (Fig. 1), the energy barrier for Au adatom diffusion on Au-(100) facets decreases when Pt is present, that is, Pt can speed up Au adatom diffusion by lowering the energy barrier of migration pathway. A faster migration rate allows more Au adatoms to migrate to the initially nucleated sites at a given time, which thus increases the degree of morphological asymmetry. This theory is further corroborated by the observation that a higher Pt content on AuNDs leads to an even higher degree of asymmetry as shown in Fig. 3. For the situation that Au nanocrystalline seeds are fully coated by thick Pt layer, Au growth can be directly considered to be on Pt surface; that is, the whole surfaces of seeds are highly lattice-mismatched to Au adatoms, indicating that Au adatoms on the surface should have a stronger trend (higher rate) to migrate to thermodynamically favorable position. Therefore, once a nucleation site is formed on either side of the seed, all Au adatoms from the other positions quickly migrate to the nucleated position due to its higher activity and smaller lattice mismatch, further causing selective growth only on that specific side^56^.

### AuNR- and AuNB-based nanocrystals with controlled geometrical symmetry

**Fig. 5:**
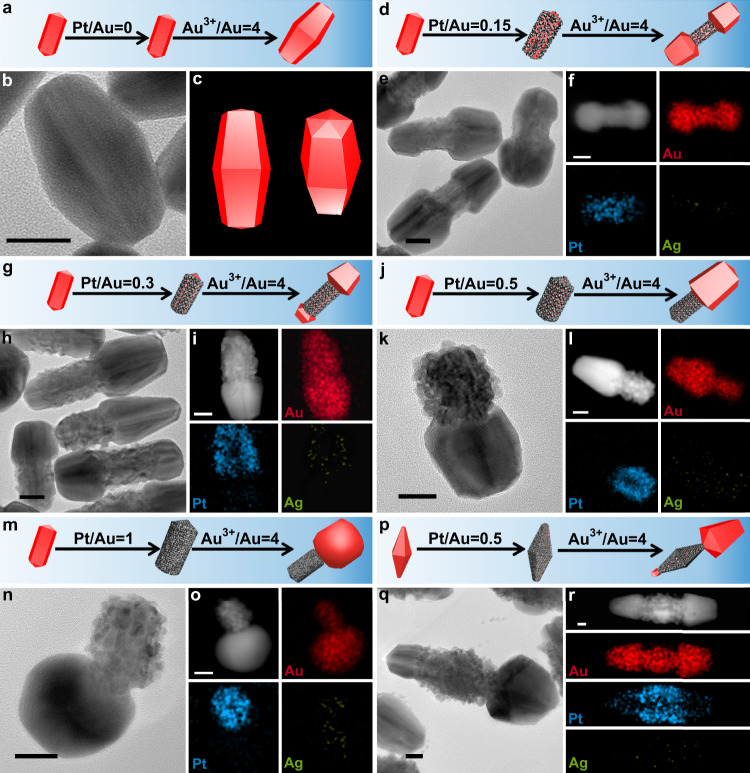
Au growths on AuNR@Pt and AuNB@Pt nanocrystals. TEM images, STEM-HAADF images, STEM-EDS elemental maps, and schematic illustrations of products prepared through overgrowing Au on AuNR@Pt nanocrystals with different Pt/Au molar ratios: **a**, **b**, **c** 0; **d**, **e**, **f** 0.15; **g**, **h**, **i** 0.3; **j**, **k**, **l** 0.5; **m**, **n**, **o** 1. **p**, **q**, **r** TEM images, STEM-HAADF images, STEM-EDS elemental maps, and schematic illustrations of products prepared through overgrowing Au on AuNB@Pt nanocrystals. All scale bars are 20 nm. In all schematics, the red and gray colors represent Au and Pt, respectively.

General applicability of an approach is significant in nanocrystal synthesis. Here, one-dimensional PFT AuNRs coated by different amount of Pt were also used as seeds to verify the universality of surface lattice engineering; similar to the growth on AuND@PtNCs, product spatial symmetry is very sensitive to Pt content. Pt-free AuNRs induced the formation of round-tip AuNBs (Fig. 5a–c, and Supplementary Figs. 18, 19), and their lengths along transverse and longitudinal axis are larger than that of AuNR seeds. Therefore, it can be known that the growth occurred towards both directions, but the growth rate along the latter was faster; furthermore, they are geometrically symmetrical in each direction. When AuNR@Pt nanocrystals with 0.15 or 0.3 molar ratio of Pt/Au were used as seeds (Fig. 5d–i and Supplementary Figs. 21–26), Au growth along transverse direction was completely blocked because Pt coating led to large lattice mismatch in these positions. Relatively, longitudinal growth along <110> was promoted and produced dumbbell-like AuNR-(AuNR@Pt)-AuNR nanocrystals, meaning that Pt enhances the migration of Au adatoms from side surfaces to two ends during growth process. Meanwhile, two newly formed Au domains have no equal size, and this difference grows with increase of Pt. Further rising Pt content on AuNRs (the Pt/Au molar ratio was 0.5) brought about lower-symmetry unidirectional growth, yielding Janus-like (AuNR@Pt)-AuNR nanocrystals (Fig. 5j–l and Supplementary Figs. 27–34). If the molar ratio of Pt and Au reached 1 (Fig. 5m–o and Supplementary Figs. 35–44), the growth pattern had no change. However, the majority of formed Au domains are single crystalline instead of PFT (Supplementary Fig. 39), indicating that the PFT AuNRs almost completely lost the ability to guide Au growth because of thick Pt coating. It is worth noting that the symmetry breaking has no obvious dependence on Au deposition rate. For example, when AuNRs fully coated by thick Pt layer were used as seeds, enhancing Au deposition rate (by increasing the molar ratio of HAuCl_4_/Au from 4 to 7 or 12) did not change the growth mode (Supplementary Figs. 31, 34, 38, 44); that is, growths occurred only on one side in all cases with different Au deposition rates. Therefore, the symmetry breaking relies on directional Au adatom diffusion but not on Au deposition rate.

Above shape evolution with Pt is consistent with cases of Au growths on AuND@PtNC seeds in the following two respects: (1) Pt content determines the growth symmetry, and (2) formed Au domains are unable to inherit the PFT crystalline structure of AuNDs and AuNRs when Pt coating is dense. However, the minimum Pt content on AuNRs (Pt/Au = 0.5:1 molar ratio) for achieving unidirectional growth is significantly lower than that on AuNDs (Pt/Au = 2:1 molar ratio), which might be caused by the differences in the growth selectivity of Pt on their surfaces. The growth selectivity of Pt on AuND is drastically higher than that on AuNR. For this reason, full coating of AuND surface, especially these positions far from {100} facets, requires large amount of Pt to realize unidirectional growth.

AuNBs are another type of common one-dimensional PFT nanocrystals which also served as seeds to test the effectiveness of surface lattice engineering. The growths of other metals on Pt-free AuNB have been reported extensively, and only symmetrical growth was observed so far^42,62^, but Pt-coated AuNBs seeds (Pt/Au = 0.5:1 mol/mol), similar to AuND@PtNC and AuNR@Pt, could induce an asymmetrical growth in our experiment (Fig. 5p–r and Supplementary Figs. 49, 50).

### AuTNO-based nanocrystals with controlled geometrical symmetry

**Fig. 6:**
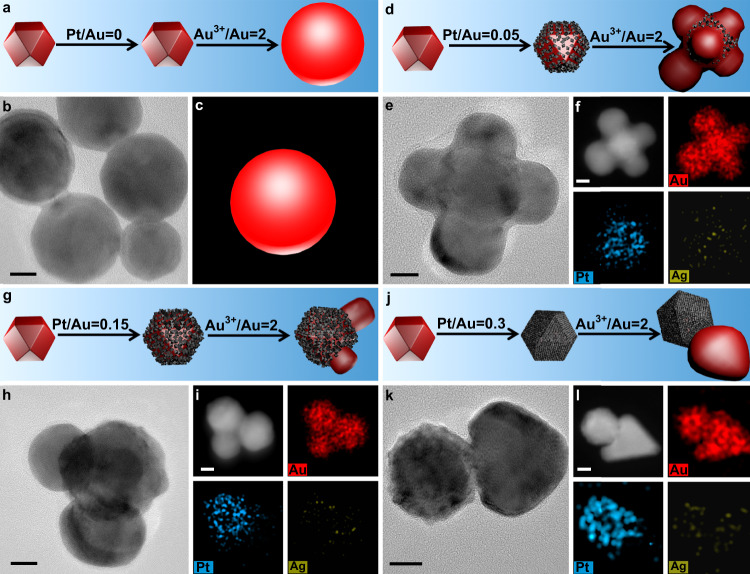
Au growths on AuTNO@Pt nanocrystals. TEM images, STEM-HAADF images, STEM-EDS elemental maps, and schematic illustrations of products prepared through overgrowing Au on AuTNO@Pt nanocrystals with different molar ratios of Pt and Au: **a**, **b**, **c** 0; **d**, **e**, **f** 0.05; **g**, **h**, **i** 0.15; **j**, **k**, **l** 0.3. All scale bars are 10 nm. In all schematics, the red and gray colors represent Au and Pt, respectively.

Above used AuND, AuNR, and AuNB are typically multi-twinned nanocrystals. To further explore whether this approach has a broader applicability, single crystalline AuTNOs seeds were also tested. Results show that this strategy has no dependence on the shape and crystalline feature of seeds, and it can be used as a general approach for controlling growth pattern and tuning spatial symmetry. For example, when Pt-free AuNTOs served as seeds (Supplementary Fig. 52), spherical particles were formed (Fig. 6a–c and Supplementary Fig. 52). Well known, sphere has highest symmetry, and therefore, the growth surrounding seed is homogeneous in this case. However, when AuTNOs coated by small amount of Pt were used as seeds (Pt/Au = 0.05:1 mol/mol), multi-lump nanocrystals were generated (Fig. 6d–f and Supplementary Figs. 53–57), implying that the growth symmetry is low relative to the growth on Pt-free AuNTOs. HRTEM images and FFT patterns show that these newly formed Au lumps grew along <100 > (Supplementary Fig. 56), which means Au adatoms selectively migrated to {100} facet and deposited there in growth process. Moreover, the size of each lump even in same particle is significantly different, so it is known the growths are not equal on various {100} facets. If the molar ratio of Pt and Au in AuNTO@Pt seeds was increased to 0.15 (Fig. 6g–i and Supplementary Figs. 58–60), the number of lumps decreased and their size difference increased, which means that the growth symmetry further reduced. When the molar ratio of Pt and Au was 0.3 or higher (Fig. 6j–l and Supplementary Figs. 61–72), Au atoms only grew on one {100} facets, and this extremely asymmetrical growth led to the formation of Janus-like (AuTNO@Pt)-Au nanocrystals. The above observations are well consistent with the results presented in Figs. 4, 5. Apparently, tuning growth symmetry via the surface lattice engineering approach is independent of the shape and crystalline structure of Au seeds.

The universality of this strategy can be further confirmed by Au growth on Pt-coated single crystalline AuNP. A few AuNPs were also formed during the preparation of AuNDs (Supplementary Fig. 77), and subsequent Pt deposition happened selectively on the {110} facets of AuNPs, yielding AuNP@PtNC nanocrystals (Supplementary Figs. 78–80). The high growth preference of Pt on AuNP is due to the bounding {110} facets which are more active than {111} facet^55^; this issue is not the main point in this article and is not discussed further. Our main concern is whether the PtNCs could play a role in determining growth symmetry. Results show that the Au growth on AuNP@PtNC followed a pattern similar to that on AuND@PtNC, and two pyramid-like Au domains with different sizes were formed on the top and bottom sides of AuNP (Supplementary Figs. 81–83).

### Spatial composition control of complex nanocrystals and predictable synthesis

**Fig. 7:**
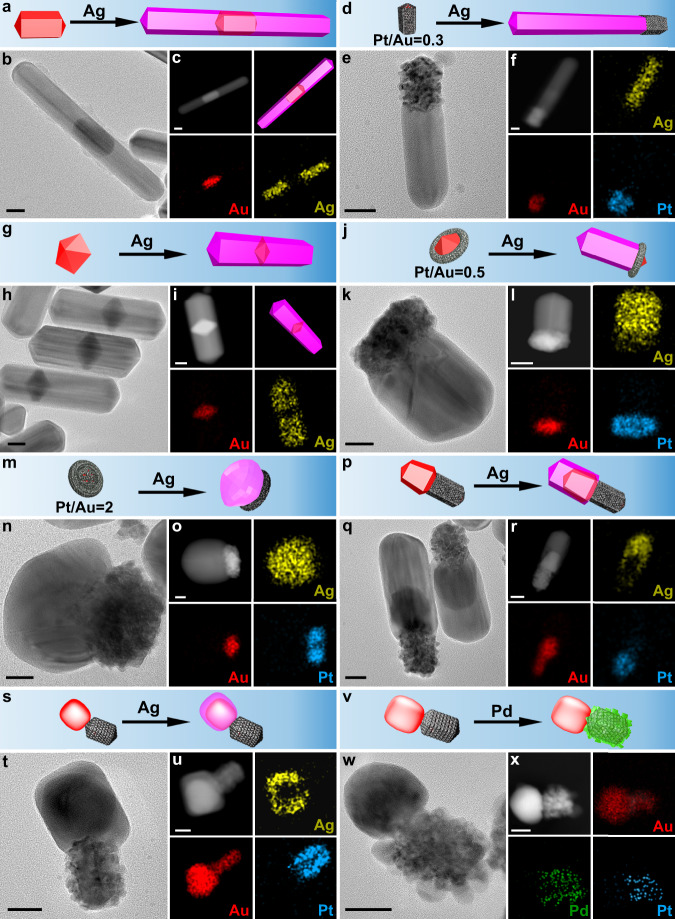
Nanocrystals syntheses with tuned spatial composition. TEM images, STEM-HAADF images, STEM-EDS elemental maps, and schematic illustrations of products prepared through growing Ag on various seeds: **a**, **b**, **c** AuNR; **d**, **e**, **f** AuNR@Pt (the molar ratio of Pt and Au was 0.3); **g**, **h**, **i** AuND; **j**, **k**, **l** AuND@PtNC (the molar ratio of Pt and Au was 0.5); **m**, **n**, **o** AuND@PtNC (the molar ratio of Pt and Au was 2); **p**, **q**, **r** nanocrystals shown in **5k**; **s**, **t**, **u** nanocrystals shown in **5n**. **v**, **w**, **x** TEM images, STEM-HAADF images, STEM-EDS elemental maps, and schematic illustrations of products prepared through growing Pd on nanocrystals shown in **5n**. All scale bars are 20 nm. In all schematics, the red, gray, mauve, and green colors represent Au, Pt, Ag, and Pd, respectively.

Lattice-mismatch-driven adatom migration is universal in crystal growth^39,56,63^, so this surface lattice engineering strategy can also offer practical guidance for the rational design and engineering of nanocrystals composed of other metals. Ag, Au, Pt, and Pd are four common noble metals, and their cell parameters are 4.086 Å, 4.078 Å, 3.924 Å and 3.891 Å, respectively^57^. Ag and Pt have a larger lattice mismatch than Au and Pt, so Pt-Ag interface should have a higher energy relative to Pt-Au interface, which is confirmed by DFT calculations (Supplementary Fig. 84). In other words, Pt-Au interface is more thermodynamically stable than Pt-Ag interface; as a result, Ag adatoms should have a stronger trend to migrate away from Pt-coated surface. The energy barrier (activation energy) for Ag adatom diffusion (migration) was also calculated under the same condition as the case of Au adatom (Fig. 1c, d), and result reveals that the energy barrier for Ag adatom migration is lower than that for Au adatom (Supplementary Fig. 85). Therefore, Ag adatom migration should be faster theoretically than Au adatom on Pt-covered Au surface. In fact, the Ag growths on AuNDs, AuND@PtNC nanocrystals, AuNRs, and AuNR@Pt nanocrystals (shown in Fig. 7) well agree with DFT studies. Pt-free AuNRs induced the formation of segmental AgNR-AuNR-AgNR nanocrystals in which almost all AuNRs are in the middle (Fig. 7a–c and Supplementary Figs. 86–88). As expected, AuNR@Pt seeds (Pt/Au = 0.3:1 mol/mol) facilitated unidirectional growth, creating (AuNR@Pt)-AgNR nanocrystals (Fig. 7d–f and Supplementary Figs. 89–92). It has been demonstrated that Au growth on the same AuNR@Pt seeds was bidirectional along <110 > (Fig. 5g–i and Supplementary Fig. 26), indicating that Ag adatom indeed migrates faster than Au adatom on Pt-coated seed surface. This is further confirmed by the similar growth of Ag on AuND-based seeds. Pt-free AuNDs led to the formation of AgNR-AuND-AgNR nanocrystals (Fig. 7g–i and Supplementary Figs. 93–95). However, for the case of AuND@PtNC seeds, only unidirectional growth occurred, and Janus-like (AuND@PtNC)-AgNR nanocrystals were generated (Fig. 7j–l and Supplementary Figs. 96, 97). AuND@PtNC seeds with a high Pt content resulted in irregular Ag domains instead of PFT AgNRs (Fig. 7m–o and Supplementary Figs. 98–101), because AuNDs were densely covered by Pt and hence incapable of effectively guiding the growth of Ag; this is similar to Au growth (Fig. 3m).

Furthermore, high-order hybrid nanocrystals with more complex structures can be obtained via rational design and predictable synthesis. For example, if the Janus-like (AuNR@Pt)-AuNR nanocrystals shown in Fig. 5k acted as seeds for Ag growth, it is predictable that Ag adatoms should selectively migrate to Pt-free Au domain and deposit there, because Ag and Pt have a larger lattice mismatch than Ag and Au^47,57^. The formation of (AuNR@Pt)–AuNR–AgNR nanocrystals confirms this (Fig. 7p–r and Supplementary Figs. 102, 103). The (AuNR@Pt)-Au seeds with single crystalline Au domain presented in Fig. 5n guided a similar growth, yielding (AuNR@Pt)-(Au@Ag) nanocrystals (Fig. 7s–u and Supplementary Figs. 104–107). The cell parameter of Pd is close to that of Pt. If the former grows on the same (AuNR@Pt)-Au seeds, there is a high probability that Pd adatoms migrate to Pt-rich positions because Pt–Pd interface has a lower energy than Pd–Au interface. HRTEM and elemental maps provide solid evidences that Pd atoms indeed deposited only on Pt-coated surface rather than Au exposed surface (Fig. 7v–x and Supplementary Figs. 108, 109).

### Dimension control of nanocrystals

**Fig. 8:**
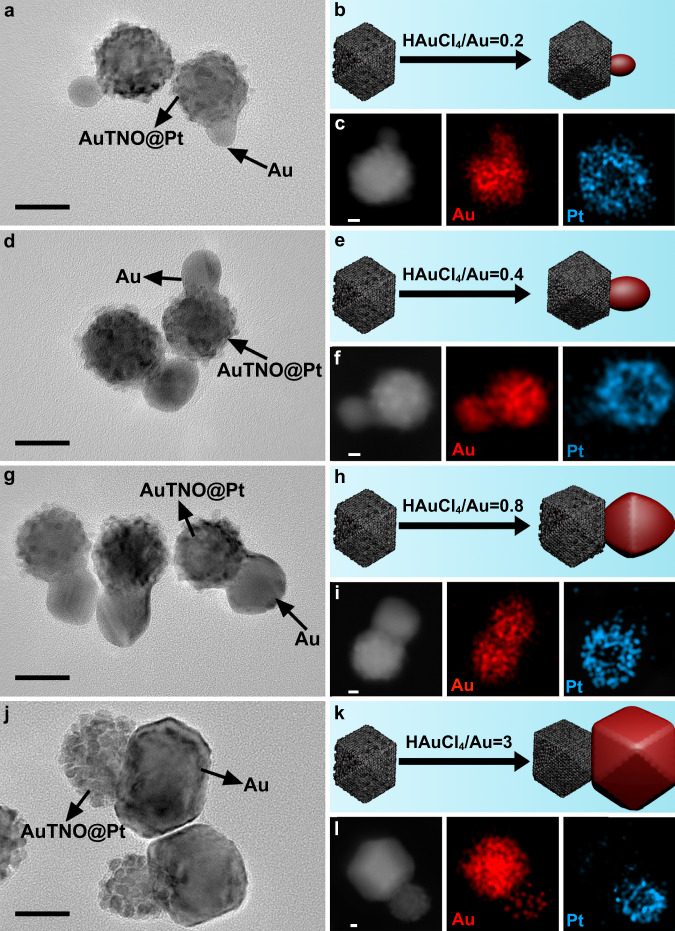
Dimension control of nanocrystals. TEM images, schematic illustrations, and elemental maps of products prepared through tuning the molar ratio of HAuCl_4_ and Au when using AuTNO@Pt nanocrystals as seeds: **a**, **b**, **c** 0.2; **d**, **e**, **f** 0.4; **g**, **h**, **i** 0.8; **j**, **k**, **l** 3. Scale bars in **a**, **d**, **g**, **j** are 20 nm. Scale bars in **c**, **f**, **i**, **l** are 5 nm. In all schematics, the red and gray colors represent Au and Pt, respectively.

The above-mentioned results distinctly demonstrate that the geometrical shape and spatial composition distribution of nanocrystals can be well controlled via the surface lattice engineering strategy reported here. Moreover, this approach also allows tuning the size dimension of each section in nanocrystals. For example, when depositing Au on AuTNO@Pt seeds, adjusting the amount of HAuCl_4_ created various sized Au domains. For low-concentration precursor (HAuCl_4_/Au = 0.2:1 molar ratio), most of formed Au domains are spheres with 13 nm diameter (Fig. 8a–c and Supplementary Fig. 73d–f). When the molar ratio of HAuCl_4_ and Au was increased to 0.4, Au domain had no significant shape change, but their average diameter increased to 18 nm (Fig. 8d–f and Supplementary Fig. 73g–i). If the molar ratio of HAuCl_4_ and Au reached 0.8 (Fig. 8g–i and Supplementary Fig. 73j–l), Au domains clearly became larger (>22 nm). Meanwhile, their shape is not uniform compared with the above two cases, and most are highly truncated octahedra. When the molar ratio of HAuCl_4_ and Au was 3, large sized polyhedral Au domains were formed (Fig. 8j–l and Supplementary Fig. 73m–o). The shape evolution of Au domains with Au precursor might be related with size effect^64^. For small particles, their surface atoms, as a consequence of low coordination number, are often very active, so energy minimization requires them to adopt spherical morphology with small surface area to compensate high energy. With the increase of Au precursor, the formation of large sized nonspherical particles is thermodynamically allowed because surface energy reduces with size. For all cases, only dimeric nanocrystals were formed even in case of high-concentration metal precursor, which implies that asymmetrical growth is caused by lattice mismatch instead of kinetics. Besides, the growths of Au domain were very fast and almost completed within 5 min in all syntheses, which does not match the typical characteristics of slow growth rate required to prepare dimeric nanocrystals using kinetic-controlled process^65^.

As well as the formed Au domains, AuTNO seeds can be tuned in size. That is, this strategy also has no dependence on the size of Au seeds. For example, the AuTNO seeds for synthesizing nanocrystals shown in Fig. 8 have 24 nm edge length. When Pt-coated large AuTNOs with 54 nm edge length were used as seeds, a similar growth mode was observed and also created Janus-like (AuTNO@Pt)-Au nanocrystals (Supplementary Figs. 74–76). Such control is also suited to the case of PFT Au seeds (Supplementary Figs. 22, 23, 30, 31, 34, 37, 38, 44, 45e, 46), in which the length of AuNRs seeds was tunable and the size of formed Au domain could be easily controlled through introducing different amount of HAuCl_4_.

### Multifunctional nanocrystals integrating surface enhanced Raman scattering (SERS) and catalytic activity

**Fig. 9:**
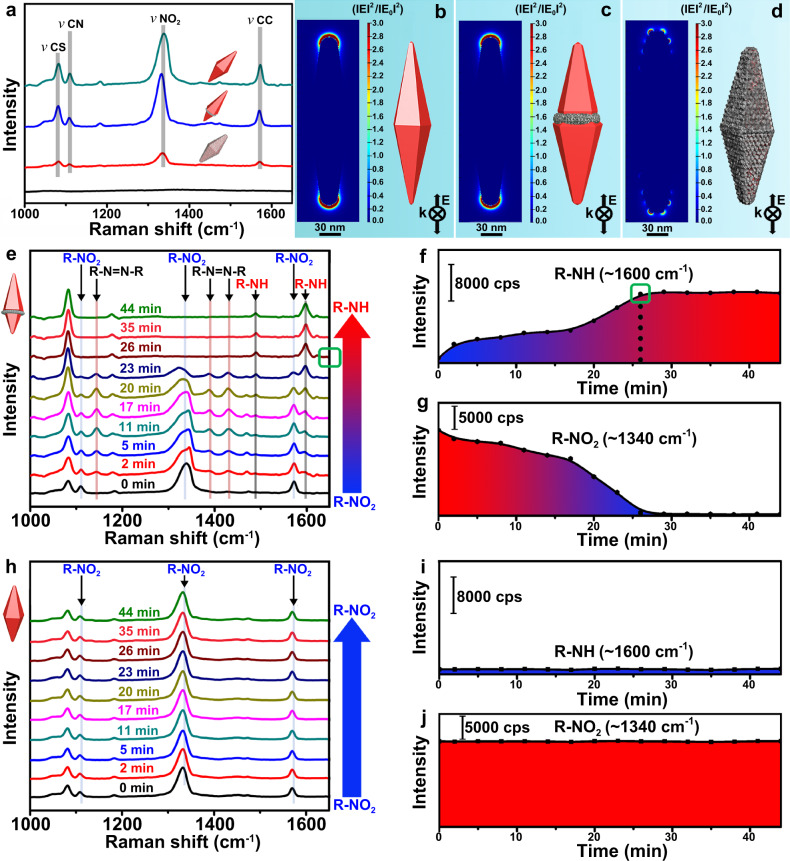
Optical properties and in situ monitoring organic reaction with SERS. **a** Raman spectra recorded on AuNB@PtNC (longitudinal length: 236 nm. Transverse length: 72 nm), AuNB (longitudinal length: 230 nm. Transverse length: 70 nm) and (AuNB-core)@(Pt-shell) nanocrystals. **b**, **c**, **d** Electrical field enhancement on the surface of various nanocrystals. Raman spectra, peak intensity of R-NO_2_ at 1340 cm^−1^ and peak intensity at 1600 cm^−1^ with time when using **e**, **f**, **g** AuNB@PtNC nanocrystals and **h**, **i**, **j** AuNBs as catalysts. In all schematics, the red and gray colors represent Au and Pt, respectively. Source data are provided as a Source Data file.

In addition to the fine control over spatial configuration, multi-functionalization of nanocrystals also can be achieved using this surface lattice engineering strategy. For example, AuNBs have excellent SERS performance because their sharp tips (hot spots) leads to a larger enhancement of local electric field relative to other Au nanocrystals^42,66–68^, but they are often inactive in chemical reactions^14^. On the contrary, Pt nanocrystals have high catalytic activity but very poor SERS performance^54,69,70^. Combination of both advantages into a single hybrid nanosystem is evidently attractive, which however cannot be realized with conventional approaches. In conventional synthesis, AuNBs are pre-synthesized, followed by Pt deposition; despite improved catalytic performance, SERS activity of AuNBs greatly reduces because of Pt coating on their sharp tips^71^. Interestingly, such combination is easily implemented in the synthesis of AuNB@PtNC nanocrystals. Surface lattice engineering requires that Pt deposition is ahead of the growth of AuNBs. This reverse arrangement completely eliminates the effect of Pt on SERS activity, as demonstrated by the following SERS measurements. 4-nitrothiophenol (4-NTP) probe molecules on AuNB@PtNCs exhibit a strong Raman signal comparable to that on AuNBs (Fig. 9a). However, the Raman signal recorded on (AuNB-core)@(Pt-shell) nanocrystals prepared with conventional method is five times weaker. Electric field enhancement simulations further confirm above result (Fig. 9b–d). In situ monitoring the catalytic reduction of 4-NTP to 4-aminothiolphenol (4-ATP) with SERS demonstrates that AuNB@PtNC nanocrystals also have enhanced catalytic activity thanks to the presence of PtNCs. For AuNB@PtNC catalyst, the spectra band corresponding to 4-NTP gradually decreased with time (Fig. 9e–g). Three new Raman bands of intermediate (4,4′-dimercapto-azobenzene) at 1143, 1388 and 1430 cm^−1^ were observed after 2 min;^14,20,54^ these bands gradually increased, then progressively decreased, and entirely disappeared after 26 min. In addition, two new bands (1490 and 1600 cm^−1^) attributable to 4-ATP appeared. Their intensity continued to increase and then became stable after 26 min. Apparently, the reaction between 4-NTP and sodium borohydride (reducing agent) was very fast, and was completed within 26 minutes. However, for the AuNB catalyst, almost no change was observed due to its low catalytic activity even after 44 min (Fig. 9h–j). The improved catalytic performance of the AuNB@PtNC nanocrystals mainly stems from the introduction of active Pt component, and furthermore, the synergistic effect of two metals also might make contributions. Pt-on-Au structure can result in electron donation from Pt to Au, which increases the 5d vacancies in Pt and hence facilitates the absorption of reactant with electron donor group^72^. Thus, the reaction is promoted. In addition, electron transfer increases the electron density on Au, and 4-NTP obtains electrons more easily; therefore, the catalytic activity of Au is also improved^52^. Deeply understanding this needs a great deal of research and will be targeted to other topics. This example was mainly used to demonstrate that advantages of various materials can be integrated together via surface lattice engineering.

## Discussion

In summary, we developed a surface lattice engineering strategy for precisely tuning the growth pattern of seeded growth and constructing hybrid nanocrystals with fine-tuned spatial configuration in shape, composition, and dimension. A variety of hybrid nanocrystals unaccessible to conventional synthetic technologies can be prepared in a controlled manner. Furthermore, the results presented in this work also may provide a potential approach for rationally designing and predictably synthesizing hybrid nanocrystals with high complexity and optimized structure. Two critical factors of this approach, namely lattice mismatch and adatom diffusion, are universal phenomena in hybrid crystalline composites, including metals, oxide metals, semiconductors, and even organic crystals^25,38,73–75^. Presumably, this strategy can be generalized and extended to various other crystalline material systems to gain broader applications.

## Methods

### Preparation of AuNDs

A total of 12.5 μL aqueous chloroauric acid (HAuCl_4_, 0.48 M) was transferred into a 50-mL beaker which was then heated in 125 °C oven. After water was removed completely, 10 mL diethylene glycol (DEG) containing 0.25 mL poly (diallyldimethylammonium chloride) (PDDA, Mw  = 400000–500000, 20 wt% in H_2_O) was added, and then the solution was stirred vigorously until a yellow homogeneous solution was formed. 3 mL DEG containing 6 mg silver nitrate (AgNO_3_) was introduced into above yellow solution. The mixture was stirred for another 3 minutes and then transferred into 25-mL round-bottom flask which was then heated in 200 °C oil bath. After 30 min, the red solution was cooled down to room temperature, and then 12 mL water was added to form seed solution for preparing AuND@PtNCs.

### Preparations of AuND@PtNCs

In total 10 mL above seed solution was heated to 80 °C, and then aqueous chloroplatinic acid (H_2_PtCl_6_, 19.3 mM) and ascorbic acid (AA, 56.8 mM) solution were introduced (AA/H_2_PtCl_6_ = 2:1 mol/mol; Pt/Au = 1:2 mol/mol). After 10 h reaction, a gray solution containing AuND@PtNCs was cooled to room temperature and could be used as seed solution for preparing AuNB@PtNC nanocrystals. To control the thickness of Pt coating, various concentrations of H_2_PtCl_6_ and AA were introduced, while the molar ratio of AA/H_2_PtCl_6_ was kept unchanged.

### Preparations of AuNB@PtNC nanocrystals

A total of 10 μL ammonia water (20% volume ratio; freshly prepared ammonia water is highly recommended) and an aqueous HAuCl_4_ solution (0.048 M) were added into 1 mL AuND@PtNC seed solution (Au^3+^/Au = 10 mol/mol). After vigorous shaking for 30 s, the mixture was sealed in 50 mL glass pressure tube (19 cm in length, 2.6 cm in outer diameter, and 4 mm wall thickness), and then it was quickly moved into a 120 °C oil bath. After 20 min, AuNB@PtNC nanocrystals could be formed.

More details of other syntheses and characterizations can be seen in **Supplementary methods**.

## Supplementary information

Supplementary Information

## Data Availability

All data generated in this study are provided within the manuscript and the Supplementary Information. Source data are provided with this paper Source data are provided with this paper.

## Source data

Source data
