# Supplementary material for: Surface lattice engineering for fine-tuned spatial configuration of nanocrystals
Source: Nat Commun. 2021 Sep 27;12:5661. doi: 10.1038/s41467-021-25969-7 (PMC8476615; doi:10.1038/s41467-021-25969-7)
Supplement: Supplementary file 1 — Supplementary Information [file 41467_2021_25969_MOESM1_ESM.pdf]

# Supplementary Information for

# Surface Lattice Engineering for Fine-Tuned Spatial Configuration of Nanocrystals

Bo Jiang[1§], Yifei Yuan[1,2§], Wei Wang[3], Kun He[1], Chao Zou[1], Wei Chen[1], Yun Yang[1*], Shun Wang[1*], Vitaliy Yurkiv[4*], Jun Lu[2*].

[1]Nanomaterials and Chemistry Key Laboratory, Wenzhou University, Wenzhou, China.

[2]Chemical Sciences and Engineering Division, Argonne National Laboratory, Argonne, IL 60439, USA.

[3]Department of chemistry and Center for pharmacy, university of Bergen, 5020 Bergen, Norway.

[4]Department of Mechanical and Industrial Engineering, University of Illinois at Chicago, Chicago, Illinois 60607, United States.

*To whom correspondence should be addressed. E-mail: bachier@163.com; shunwang@wzu.edu.cn; vyurkiv@uic.edu; junlu@anl.gov.

[§]B. Jiang and Y. Yuan contributed equally to this work

**Table of contents:**

**Supplementary methods**.........................................................................................2

**Supplementary Note 1**. Characterizations of AuND-based nanocrystals........................10

**Supplementary Note 2**. Supplementary discussion on growth mechanism.....................25

**Supplementary Note 3**. Characterizations of AuNR and AuNB-based nanocrystals..…26

**Supplementary Note 4**. Characterizations of AuTNO-based nanocrystals......................50

**Supplementary Note 5**. Characterizations of high-order complex nanocrystals…..…....77

**Supplementary Note 6**. SERS measurements..................................................................96

**Supplementary References**............................................................................................100

## Supplementary Methods

Detailed procedure of nanocrystals syntheses, structure characterizations, finite-difference time-domain (FDTD) calculations, density function theory (DFT) calculations, and surface enhanced Raman scattering (SERS) measurements are presented in this section.

### Chemicals

4-nitrophenol (4-NTP), sodium borohydride ($NaBH_4$), silver nitrate ($AgNO_3$), chloroauric acid ($HAuCl_4$), chloroplatinic acid ($H_2PtCl_6$) and poly (diallyldimethylammonium chloride) (PDDA, $M_w$ =400000-500000, 20 wt. % in $H_2O$) were purchased from Sigma-Aldrich. Aqueous ammonia, ascorbic acid (AA), cetyltrimethylammonium bromide (CTAB), and diethylene glycol (DEG) were provided by Aladdin reagent. For all chemicals, no any purification was needed before use. Deionized water (18.2 MΩ·cm) was produced with a Milli-Q Academic water purification system (Millipore Corp., Billerica, MA, USA) and used in all experiments.

### Preparation of Au nanodecahedron (AuND) particles

AuNDs were prepared following a reported method.[1] Briefly, 12.5 μL aqueous $HAuCl_4$ (0.48 M) was transferred into a 50-mL beaker which was then heated in 125°C oven. After water was removed completely, 10 mL DEG containing 0.25 mL PDDA was added, and then the solution was stirred vigorously until a yellow homogeneous solution was formed. 3 mL DEG containing 6 mg $AgNO_3$ was introduced into above yellow solution. The mixture was stirred for another 3 minutes and then transferred into 25 mL round-bottom flask, following by heating in 200°C oil bath to induce the formation of AuNDs. After 30 minutes, the red solution was cooled down to room temperature, and then 12 mL water was added to dilute the colloid. The resulting colloid served as seed solution for the synthesis of other nanostructures.

### Preparation of AuND coated by Pt nanocircle (AuND@PtNC) particles

A method reported by our group was used to prepare AuND@PtNC nanocrystals.[2] Briefly, 10 mL above seed solution was heated to 80°C, and then a calculated amount of $H_2PtCl_6$ (19.3 mM) and AA (56.8 mM) aqueous solution were introduced (AA/$H_2PtCl_6$=2:1 mol/mol). A color change from red to gray was observed with the reaction due to the deposition of Pt on AuNDs. After 10 hours reaction, the gray solution containing AuND@PtNCs was cooled to room temperature and could be used as seed solution for

growing other nanostructures directly. To control the thickness of Pt coating, various concentrations of $H_2PtCl_6$ and AA were introduced, while the molar ratio of AA/$H_2PtCl_6$ was kept unchanged.

**Preparations of Au nanorods (AuNRs)**

The preparation of AuNBs is similar to that of AuNDs, except that 50 μL DEG containing 0.1 mg $AgNO_3$ was introduced. The reaction temperature for preparing AuNRs has no change.

**Preparations of Au nanobipyramids (AuNBs)**

10 μL ammonia water (20% volume ratio) and a calculated amount of $HAuCl_4$ (0.048 M) were added into 0.5 mL as-prepared AuND solution, and then 0.5 mL water was added. After vigorous shaking for 30 seconds, the mixture was sealed in 50 mL glass pressure tube (19 cm in length, 2.6 cm in outer diameter, and 4 mm in wall thickness), and then it was quickly moved into a 120°C oil bath. After 20 minutes, the solution was cooled to room temperature.

**Preparations of AuNR@Pt and AuNB@Pt nanocrystals**

The preparations of two nanostructures are identical to that of AuND@PtNC particles, except that AuNRs and AuNBs were used as seeds.

**Preparations of nanocrystals with asymmetrical structure using AuND, AuND@PtNC, AuNR, AuNR@Pt and AuNB@Pt nanocrystals as seeds**

10 μL ammonia water (20% volume ratio) and a calculated amount of $HAuCl_4$ (0.048 M) were added into 1 mL as-prepared seed solution (which needs no purifications). After vigorous shaking for 30 seconds, the mixture was sealed in 50 mL glass pressure tube (19 cm in length, 2.6 cm in outer diameter, and 4 mm in wall thickness), and then it was quickly moved into a 120°C oil bath. After 20 minutes, the solution was cooled to room temperature.

**Note:** Freshly prepared ammonia water is highly recommended.

**Preparation of (AuNB-core)@(Pt-shell) nanocrystals in aqueous solution**

**Note:** To distinguish the products from the AuNB@Pt nanocrystals shown in **Supplementary Fig. 44c**, the nanocrystals prepared in CTAB system are named as (AuNB-core)@(Pt-shell).

The used AuNBs were prepared through overgrowing Au on AuNDs (See Preparations of Au nanobipyramids). Purified AuNBs (0.012 mmol) are dispersed into 20 mL 0.05 M

CTAB aqueous solution. $H_2PtCl_6$ solutions (19.3 mM) was introduced into above solution and the Pt/Au molar ratio (Here Au is referred to that in AuNDs; Pt/Au=0.5 mol/mol) was 0.5. A calculated amount of AA (56.8 mM) was added to serve as reducing agents (The molar ratio of AA and $H_2PtCl_6$ was 2). The resulting solution was mixed homogeneously and then put into 80$^{o}$C oven for 3 hours.

**Preparation of segmented AgNR-AuNR-AgNR nanocrystals**

The as-prepared AuNRs was diluted using equal volume of water containing 0.2 M CTAB, and then 1 mL was used as seeds to grow segmented AgNR-AuNR-AgNR nanocrystals. 44 μL AA (56.8 mM) and 44 μL $AgNO_3$ (28.4 mM) aqueous solutions were added into above solution under magnetic stirring. After 2 minutes, the solution was moved to 60$^{o}$C oil bath for 6 hours.

**Preparation of segmented AgNR-AuND-AgNR nanocrystals**

This procedure is similar to the preparation of segmented AgNR-AuND-AgNR NRs, except that AuNDs acted as seeds.

**Preparation of (AuNR@Pt)-Ag Janus-like nanocrystals**

This procedure is similar to the preparation of segmented AgNR-AuND-AgNR nanocrystals, except that AuNR@Pt nanocrystals acted as seeds (Pt/Au=1:2 mol/mol).

**Preparation of (AuNR@Pt)-AgNR nanocrystals**

This procedure is similar to the preparation of segmented AgNR-AuND-AgNR nanocrystals, except that AuNR@Pt nanocrystals acted as seeds (Pt/Au=0.3:1 mol/mol).

**Preparation of segmented (AuNR@Pt)-AuNR-AgNR nanocrystals**

This procedure is similar to the preparation of segmented AgNR-AuND-AgNR nanocrystals, except that (AuNR@Pt)-AuNR nanocrystals, which were prepared by depositing Au (the molar ratio of $HAuCl_4$/Au is 4:1) on AuNR@Pt (in which the molar ratio of Pt/Au in AuNR@Pt is 0.5:1) nanocrystals, acted as seeds.

**Preparation of (AuNR@Pt)-(Au@Ag) nanocrystals**

This procedure is similar to the preparation of (AuNR@Pt)-AuNR-AgNR nanocrystals, except that (AuNR@Pt)-Au nanocrystals, which were prepared by depositing Au (the molar ratio of $HAuCl_4$/Au is 4:1) on AuNR@Pt (in which the molar ratio of Pt/Au in AuNR@Pt is 1:1) nanocrystals, acted as seeds.

**Preparation of Au truncated nanooctahedron (AuTNO) particles**

The preparation of truncated Au octahedron nanocrystals is similar to that of AuNDs[1,2],

except that 17 μL DEG containing 0.068 mg $AgNO_3$ was introduced. The reaction temperature was 220°C.

**Preparation of truncated AuTNO@Pt nanocrystals**

The preparations of truncated AuTNO@Pt nanocrystals are identical to that of AuND@PtNC nanocrystals, except that AuTNO nanocrystals were used as seeds. Moreover, lower molar ratios of Pt/Au were used (Pt/Au=0.05:1 mol/mol, 0.15:1 mol/mol, 0.3:1 mol/mol, 0.5:1 mol/mol), compared with the syntheses of AuND@PtNC nanocrystals.

**Preparation of complex nanocrystals by using truncated AuTNO@Pt nanocrystals as seeds**

The preparation is similar to the synthetic procedure of AuNB@PtNC nanocrystals except that various molar ratios of $HAuCl_4$ and Au were used.

**Preparation of (AuNR@Pt@Pd)-Au nanocrystals**

Asymmetrical (AuNR@Pt)-Au nanocrystals, which were prepared by depositing Au (the molar ratio of $HAuCl_4$/Au is 4:1) on AuNR@Pt (in which the molar ratio of Pt/Au in AuNR@Pt is 0.5:1) nanocrystals, acted as seeds to prepare (AuNR@Pt@Pd)-Au nanocrystals. In 2.5 mL CTAB solution (50 mM), 200 μL prepared (AuNR@Pt)-Au nanocrystals solution (5 mM, in terms of the mass of AuNR) were introduced, and then 200 μL $H_2PdCl_4$ (10 mM) and 35.2 μL AA (56.8 mM) were added. The solution was stirred for 5 minutes, and then heated in 90°C water bath for 30 minutes to finish the growth.

**Purification of products**

All products were purified using repeated centrifugal precipitation and re-dispersion (Three cycles were needed). For products with different sizes, centrifugal speed and time should be adjusted (Details can be seen in **Supplementary Table 1**).

**Supplementary Table 1.** Centrifugal speed and time for products shown in different Figs.

| Products | Centrifugal speed (rpm) | Centrifugal time (Minute) | Product | Centrifugal speed (rpm) | Centrifugal time (minute) |
|---|---|---|---|---|---|
| **Supplementary Fig. 1a, Fig. 1b** | 9000 | 20 | **Supplementary Fig. 51** | 8000 | 20 |
| **Supplementary Fig. 1c** | 8000 | 20 | **Supplementary Fig. 52** | 7000 | 20 |
| **Supplementary Fig. 1d** | 6000 | 20 | **Supplementary Fig. 53** | 8000 | 20 |
| **Supplementary Fig. 10a, 10e, 10i,** | 4000 | 20 | **Supplementary Fig. 54** | 5000 | 20 |
| **Supplementary Fig. 10m** | 3000 | 20 | **Supplementary Fig. 58** | 8000 | 20 |
| **Supplementary Fig. 19** | 7000 | 20 | **Supplementary Fig. 59** | 5000 | 20 |
| **Supplementary Fig. 20d** | 5500 | 10 | **Supplementary Fig. 61** | 7500 | 20 |
| **Supplementary Fig. 21** | 7000 | 20 | **Supplementary Fig. 62** | 5000 | 20 |
| **Supplementary Fig. 22** | 5500 | 10 | **Supplementary Fig. 66** | 7000 | 20 |
| **Supplementary Fig. 23** | 4500 | 10 | **Supplementary Fig. 69** | 6000 | 20 |
| **Supplementary Fig. 25** | 7000 | 15 | **Supplementary Fig. 73a** | 7500 | 20 |
| **Supplementary Fig. 26** | 4500 | 10 | **Supplementary Fig. 73d** | 7500 | 20 |
| **Supplementary Fig. 27** | 6500 | 20 | **Supplementary Fig. 73g** | 7000 | 20 |
| **Supplementary Fig. 30** | 6000 | 15 | **Supplementary Fig. 73j** | 7000 | 15 |
| **Supplementary Fig. 31** | 5000 | 15 | **Supplementary Fig. 73m** | 5000 | 15 |
| **Supplementary Fig. 35** | 6000 | 20 | **Supplementary Fig. 74** | 6000 | 20 |
| **Supplementary Fig. 37** | 5000 | 20 | **Supplementary Fig. 75** | 5000 | 10 |
| **Supplementary Fig. 38** | 4000 | 15 | **Supplementary Fig. 76** | 3000 | 10 |
| **Supplementary Fig. 44** | 3000 | 20 | **Supplementary Fig. 86** | 3000 | 10 |
| **Supplementary Fig. 45a** | 5000 | 20 | **Supplementary Fig. 89, 93** | 3000 | 10 |
| **Supplementary Fig. 45c** | 4000 | 15 | **Supplementary Fig. 96** | 6500 | 15 |
| **Supplementary Fig. 45e** | 2000 | 10 | **Supplementary Fig. 100** | 6000 | 20 |
| **Supplementary Fig. 46** | 1000 | 10 | **Supplementary Fig. 102** | 4000 | 20 |
| **Supplementary Fig. 49a** | 5000 | 20 | **Supplementary Fig. 104** | 4000 | 20 |
| **Supplementary Fig. 49c** | 4000 | 15 | **Supplementary Fig. 108** | 4000 | 20 |
| **Supplementary Fig. 49e** | 2000 | 10 | **Supplementary Fig. 113** | 3000 | 20 |

**Instrumentation and characterizations**

For high resolution transmission electron microscopy (HRTEM), transmission electron microscopy (TEM), high angle annular field (HAADF), scanning transmission electron microscopy energy dispersive spectroscopy (STEM-EDS), and scanning electron microscopy (SEM) analyses, 10 μL purified colloid was deposited on copper-grid-supported thin carbon film and dried at 80°C. A JEOL 2100F microscope with 200 KV accelerating voltage was used to observe the dried samples. HAADF images were recorded using a JEOL 2100F microscope with an attached STEM-EDS device. The SEM images were obtained using a Nova NanoSEM 200 (10 kV). Shimadzu 2450 UV-Vis spectrophotometer was employed to record the UV-Vis absorption spectra.

**SERS measurements**

The purified nanostructures (3.73 μmol Au in each case) were each functionalized with 1 mL ethanol solution of 4-nitrophenol (4-NTP) ($10^{-4}$ M, $10^{-5}$ M, $10^{-6}$ M) for 1 hour. After washing with deionized water twice, these 4-NTP-functionalized nanocrystals were redispersed in 1.5 mL water for SERS test. The Raman spectra were recorded using a RENISHAW Rama-scope micro-Raman system coupled with a Leica microscope using a100×objective. The excitation wavelength was 785 nm, in conjunction with a grating of 1200 lines/mm, at a power of 100% of the laser output. Data was collected from the solution phase with a collection time of 30 seconds for all samples. A sample cell was fabricated from a quartz block by punching a small hole to hold 50 μL of liquid sample. After the quartz block was attached to a glass slide, we loaded the sample solution and then placed a glass cover slip on top of the quartz block to prevent solvent evaporation.

**In situ SERS monitoring of the reduction of 4-NTP by AuNB@PtNC and AuNB nanocatalysts**

The purified nanostructures (3.73 μmol Au in each case) were dispersed in 1 mL of deionized water containing 4-NTP ($10^{-5}$ M) and incubated at room temperature for 1 hour. The NTP-functionalized AuNB@PtNC nanocrystals were then washed with water twice and dissolved in 1.5 mL deionized water. Upon mixing 300 μL of the suspension with 10 μL of aqueous $NaBH_4$ (0.01 M) in a 2 mL glass bottle, AuNB@PtNC nanocrystals were allowed to catalyze the 4-NTP reduction reaction at room temperature. We withdrew 20 μL of sample from the reaction solution every several minutes and placed this sample in a quartz cell for monitoring the progress of the reaction by SERS (Details about quartz cell

can be seen in **Supplementary Note 6**). Successive SERS spectra were collected until the complete transformation from 4-NTP to 4-aminothiolphenol (4-ATP). All SERS spectra were recorded from in solution using a 50 × objective. The data was collected with a collection time of 10 seconds at a power of 100% of the output of 785 nm laser. In cases of AuNB catalysts, a similar procedure was used.

**FDTD simulations**

FDTD solution 8.16, developed by Lumerical Solutions Inc., was used to perform simulations. In order to satisfy the experimental dielectric data, the dielectric function of Au and Pt was represented by fitting the experimental data points of Johnson and Christy.[3] We used an electromagnetic pulse (wavelength ranging from 400 to 1000 nm) as a propagating plane wave source and introduced into a box containing nanoparticles for simulating their interaction. The nanoparticles and their surrounding medium inside the box were divided into 0.25 nm meshes. The propagation direction of plane wave was set perpendicular to the longitudinal axis of nanostructures. In all SERS tests, water was used, and therefore, its refractive index (1.333) was used. The shape, composition, and size were obtained according to TEM results.

**DFT Calculations**

DFT calculations were performed using the Vienna Ab Initio Simulations Package (VASP) code employing the generalized-gradient approximation (GGA) using the PBE (Perdew, Burke, and Ernzerhof) functional to account for the exchange-correlation effects.[4-7] For systems with an even number of electrons non spin-polarized calculations, and for systems with an odd number of electrons unrestricted spin-polarized calculations, are performed. For all calculations, a cutoff energy of 400 eV is used. All structural optimizations are carried out until the forces, acting on atoms, are below 0.01 eV/Å. The criterion for energy change is set to 0.1 meV. A vacuum layer of 15 Å is used to prevent the interactions between the periodic structures in the direction perpendicular to the surface. For all surface total energy calculations, we employed a 4 × 4 × 1 k-point mesh for the integration of the Brillouin zone (BZ). The Au and Pt migration barriers are calculated using the Nudged Elastic Band (NEB) method as implemented in VASP code.[8, 9] Depending upon the configuration, there are five to nine images (structures) describing ion migration coordinates in the NEB calculations. For each diffusion coordinate, the maximum energy difference among all images is considered as the migration barrier for diffusion. All

images are simultaneously optimized along the diffusion path until the forces acting on the atoms in each image converged to 0.1 eV/Å.

The model was built based on the following analysis. For an AuND particle, it is bounded by 10 {111} facets and 5 small {100} facets. When Pt atoms are deposited on AuNDs, they prefer to nucleate on {100} facets, and so the diffusion on Au-(100) was simulated to explore the effect of Pt.

**Supplementary Note 1. Characterizations of AuND-based nanocrystals**

This note mainly presents the structure characterization of AuND nanocrystals, AuND@PtNC nanocrystals, and their derivative products.

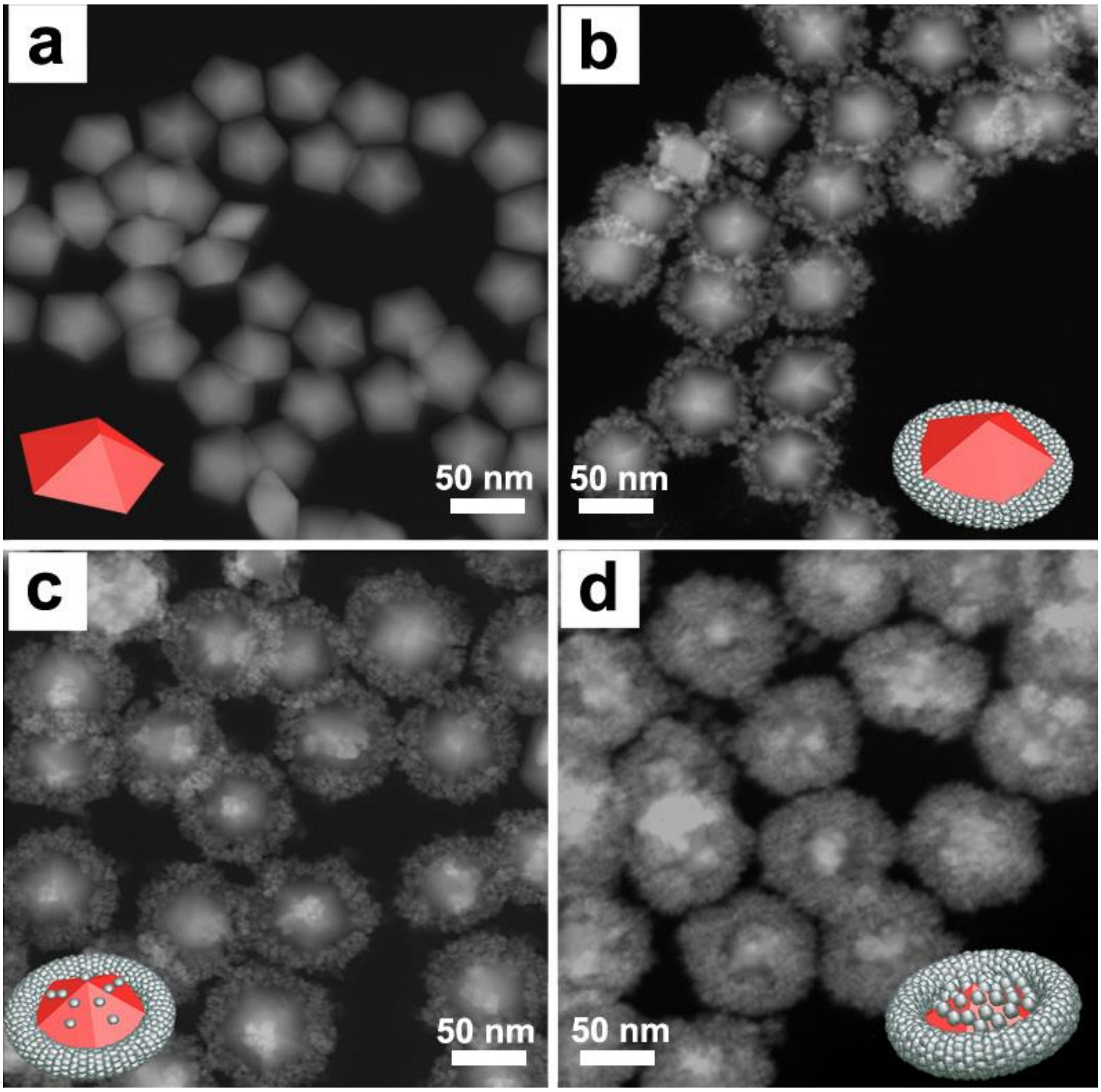


**Supplementary Fig. 1** HAADF images of AuND@PtNC nanocrystals with different Pt/Au molar ratios: **a** 0, **b** 0.5, **c** 1, **d** 2. When Pt/Au molar ratio was 2, AuND nanocrystals are almost coated completely by Pt, and therefore, it is difficult to observe decahedral feature, even on {111} facets with less Pt; this affects the further overgrowth of Au and resulting product structure. The insets in **a**, **b**, **c**, and **d** are corresponding schematic illustrations.

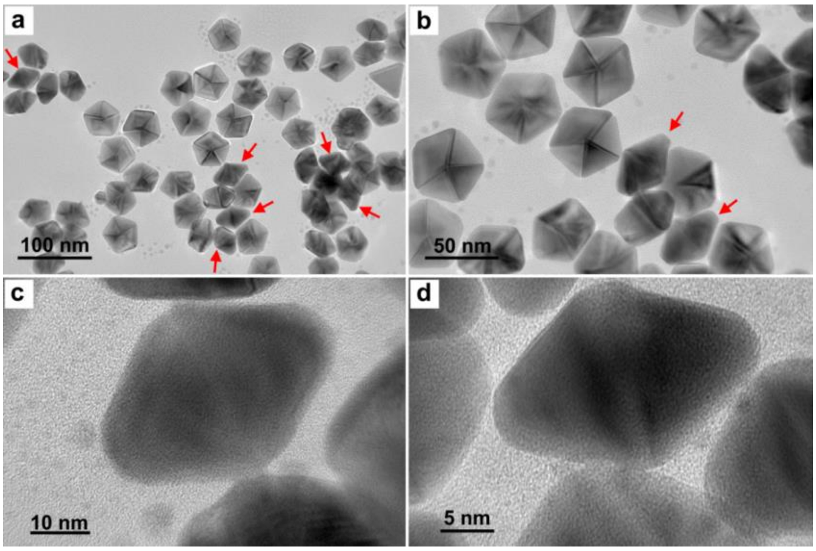


**Supplementary Fig. 2 a**, **b** TEM images and **c**, **d** HRTEM images of AuND nanocrystals. When AuND nanocrystals are appropriately positioned (as shown by the red arrows in **a** and **b**), it can be seen that the five equal edges of AuND nanocrystals are small-area {100} facets.

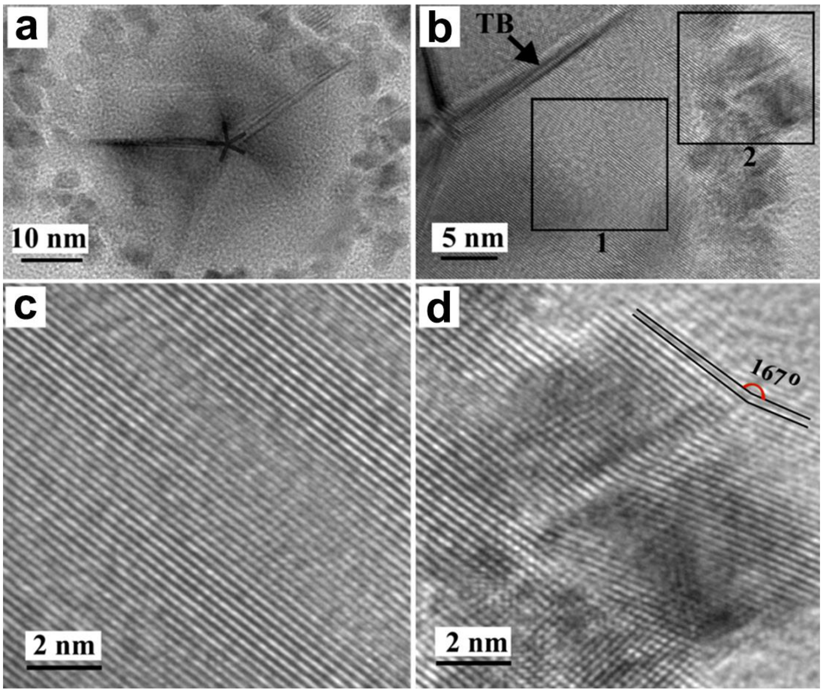


**Supplementary Fig. 3 a**, **b** HRTEM images of an individual AuND@PtNC nanocrystal from products shown in Supplementary Fig. 1b. **c** and **d** are the detailed structures of areas marked by the rectangles 1 and 2 in **b**, respectively. 167° included angle in **d** indicates the formation of twinned interface due to Pt deposition.

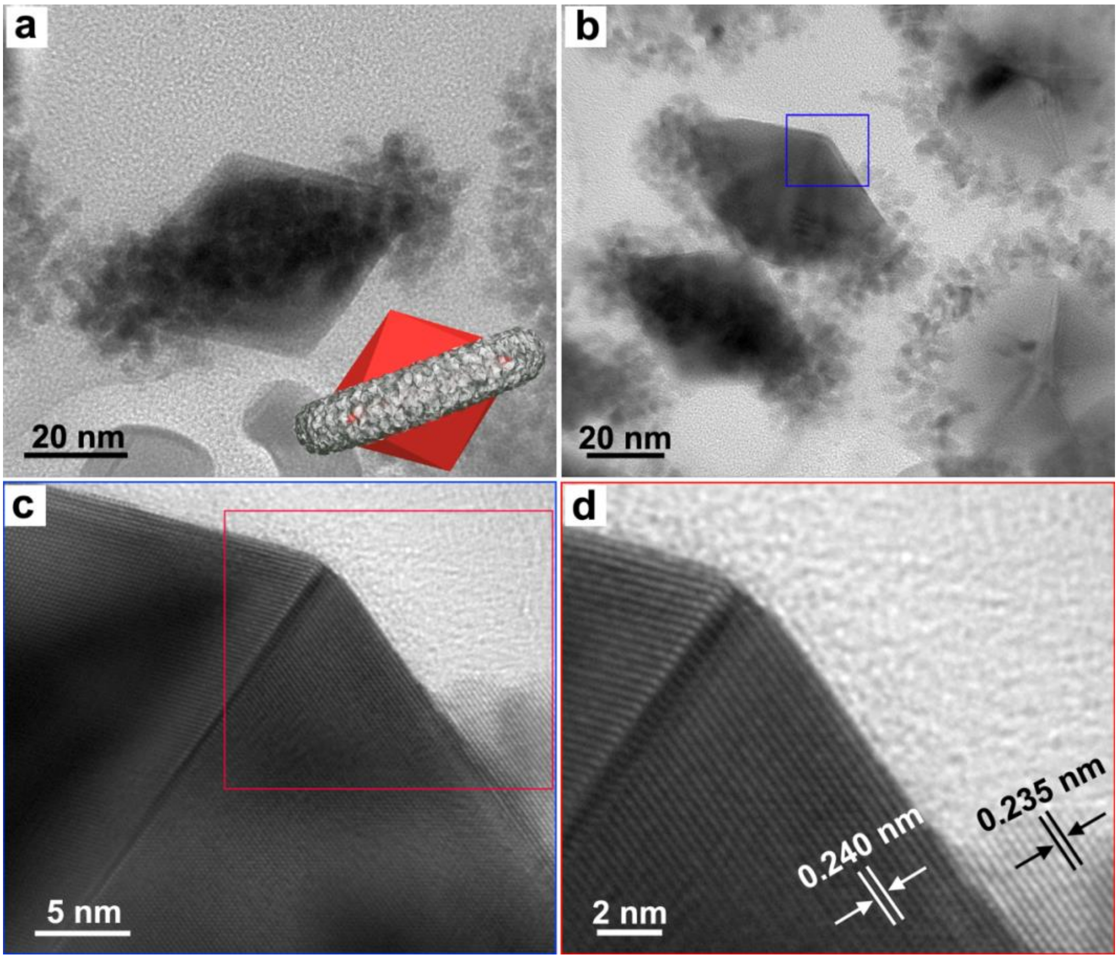


**Supplementary Fig. 4** **a**, **b** HRTEM images of AuND@PtNCs (Pt/Au=1:2 mol/mol). **c** Detailed structure of area marked by the blue box in **b**. **d** Detailed structure of area marked by the red box in **c**.

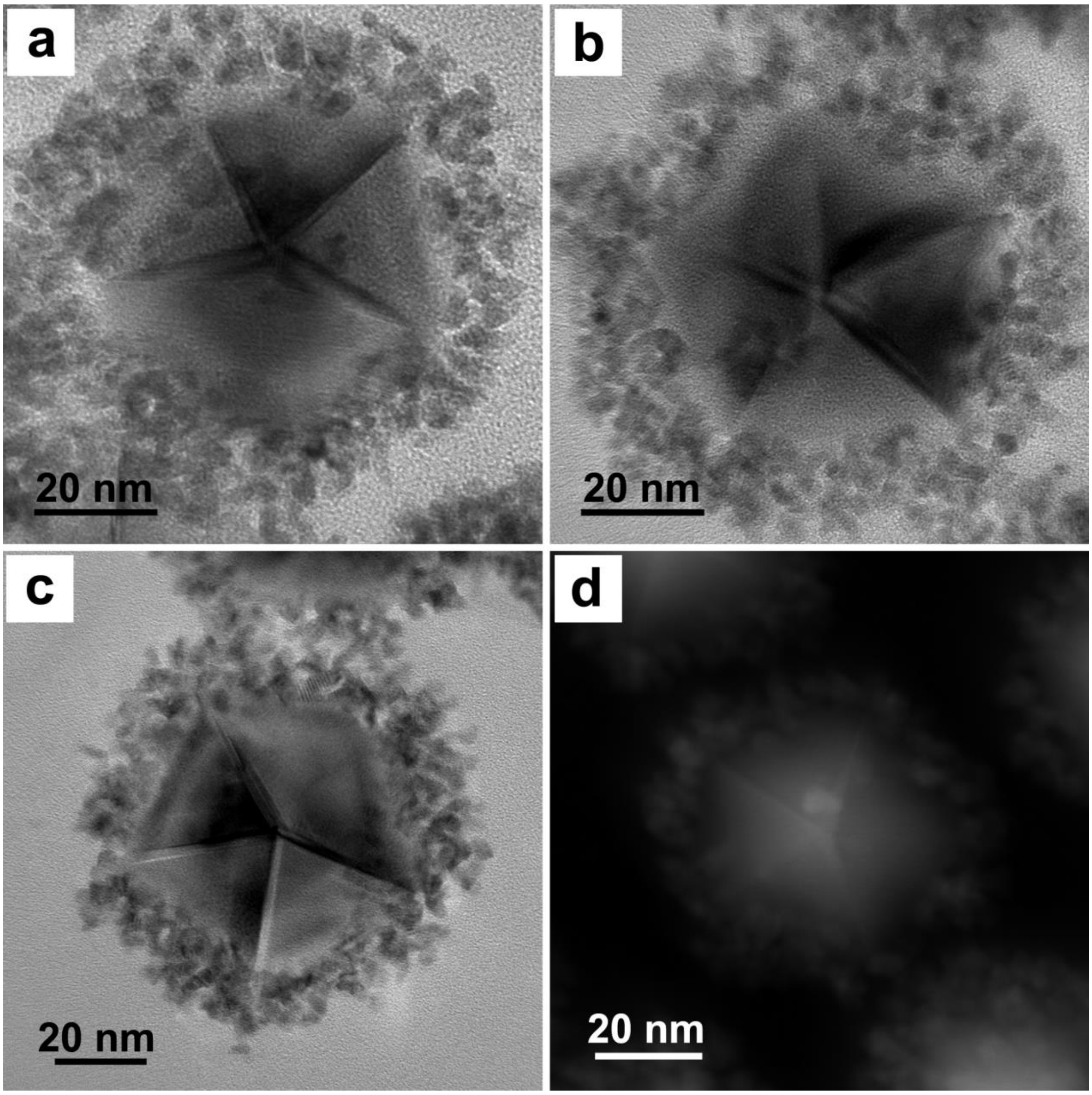


**Supplementary Fig. 5 a**, **b**, **c**, **d** HRTEM images of AuND@PtNCs (Pt/Au=1:2 mol/mol). Clearly, Pt layers consist of small Pt nanoparticles, that is, Pt deposition is island-like.

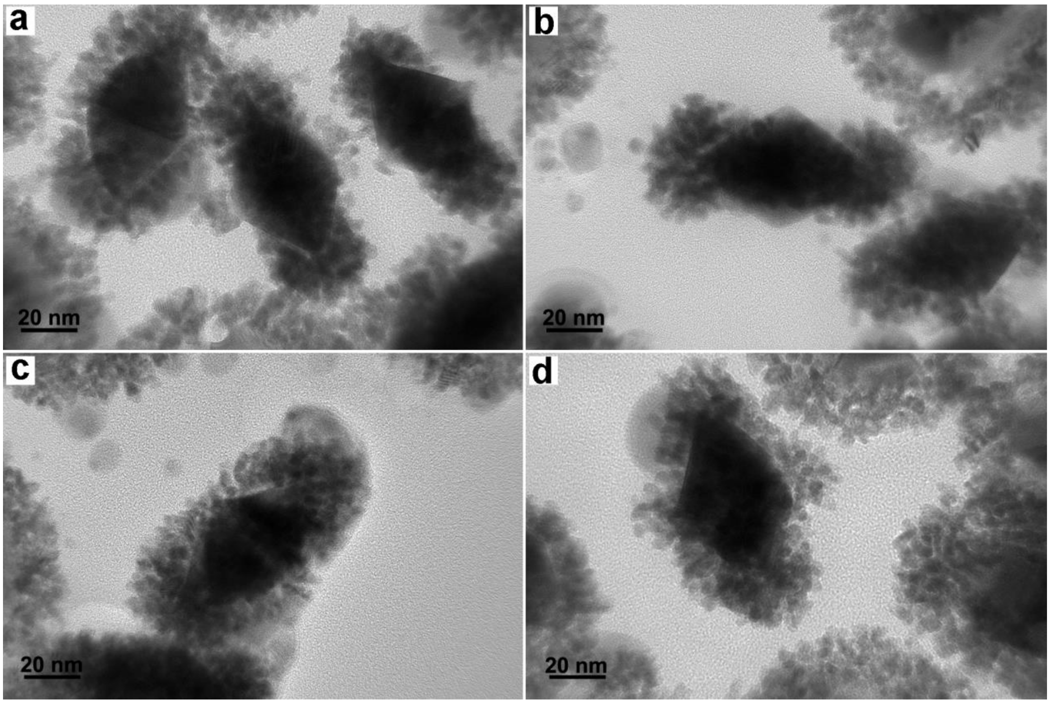


**Supplementary Fig. 6 a**, **b**, **c**, **d** HRTEM images of AuND@PtNCs (Pt/Au=1:1 mol/mol). Clearly, the AuND nanoparticles are not homogeneously coated by Pt; some of them have exposed Au tips, but others' Au tips are coated by Pt.

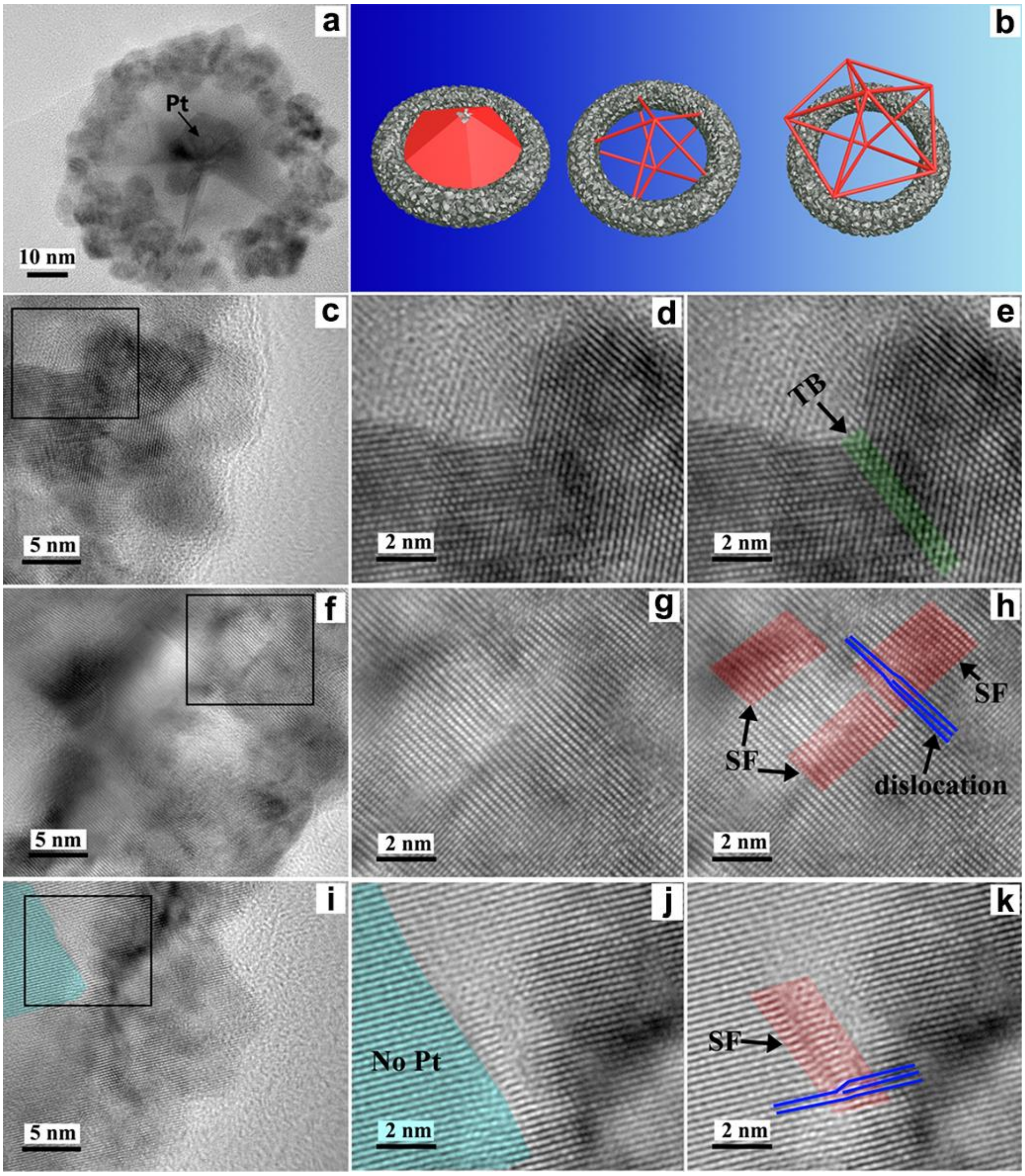


**Supplementary Fig. 7** **a** TEM image, **b** schematic illustration, and **c**, **d**, **e**, **f**, **g**, **h**, **i**, **j**, **k** HRTEM images of one AuND@PtNC nanocrystal from products shown in Supplementary Fig. 1c. **d**, **e** Detailed structure of area marked by the black box in **c**. **g**, **h** Detailed structure of area marked by the black box in **f**. **j**, **k** Detailed structure of area marked by the black box in **i**. Twinned boundary (TB), dislocation, and stacking fault (SF) are marked by green, blue, and red colors in **e**, **h**, and **k**, respectively. The TEM image in **a** clearly shows that the deposition of some Pt atoms occurred on {111}.

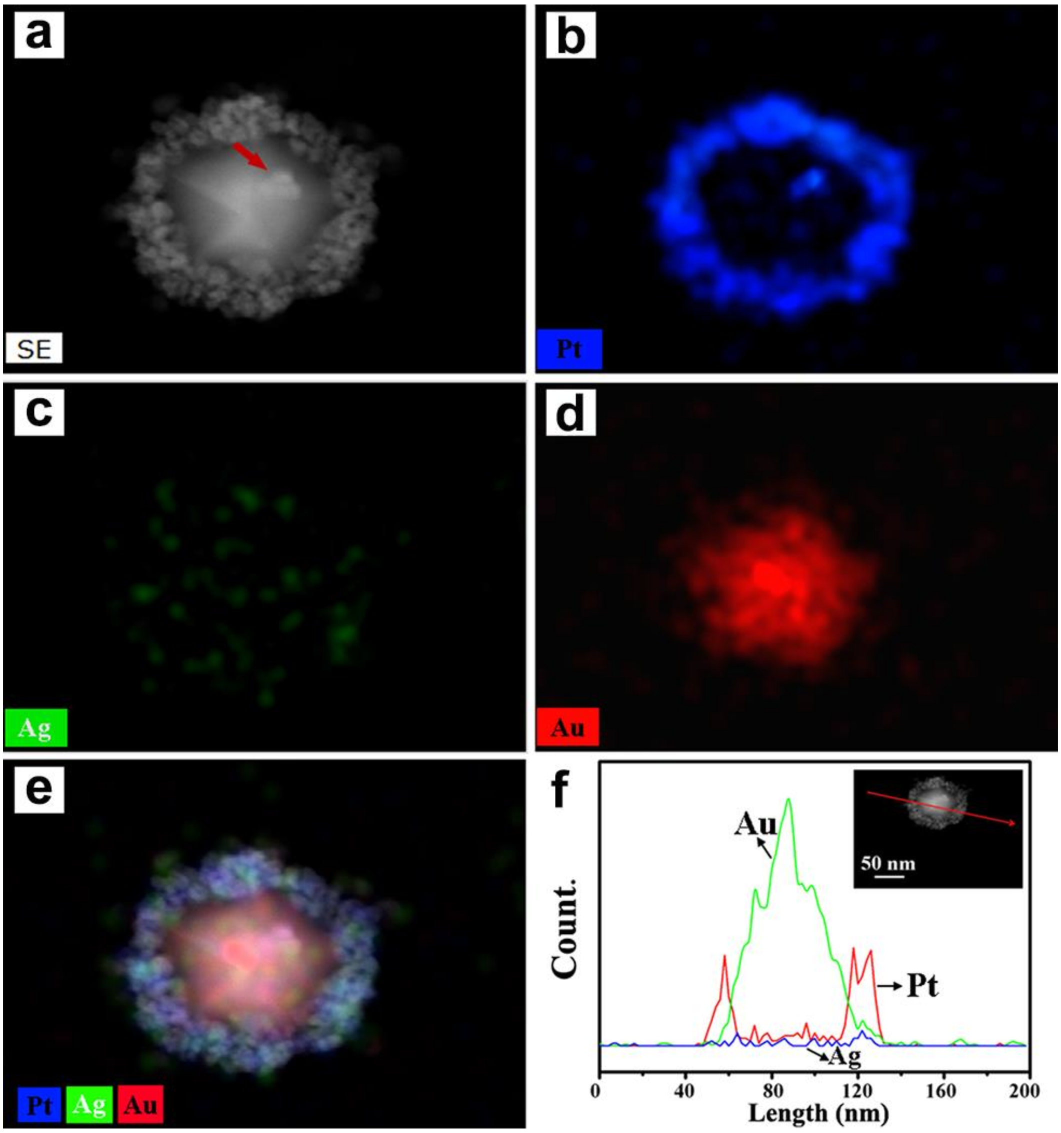


**Supplementary Fig. 8** **a** HAADF image, **b**, **c**, **d**, **e** element maps, and **f** composition line profiles of an individual AuND@PtNC nanocrystal from products shown in Supplementary Fig. 1c. The red arrow in **a** indicates that some Pt nanoparticles were formed on the {111} facet of AuND particle. The red arrow in **f** represents the direction of composition line scanning.

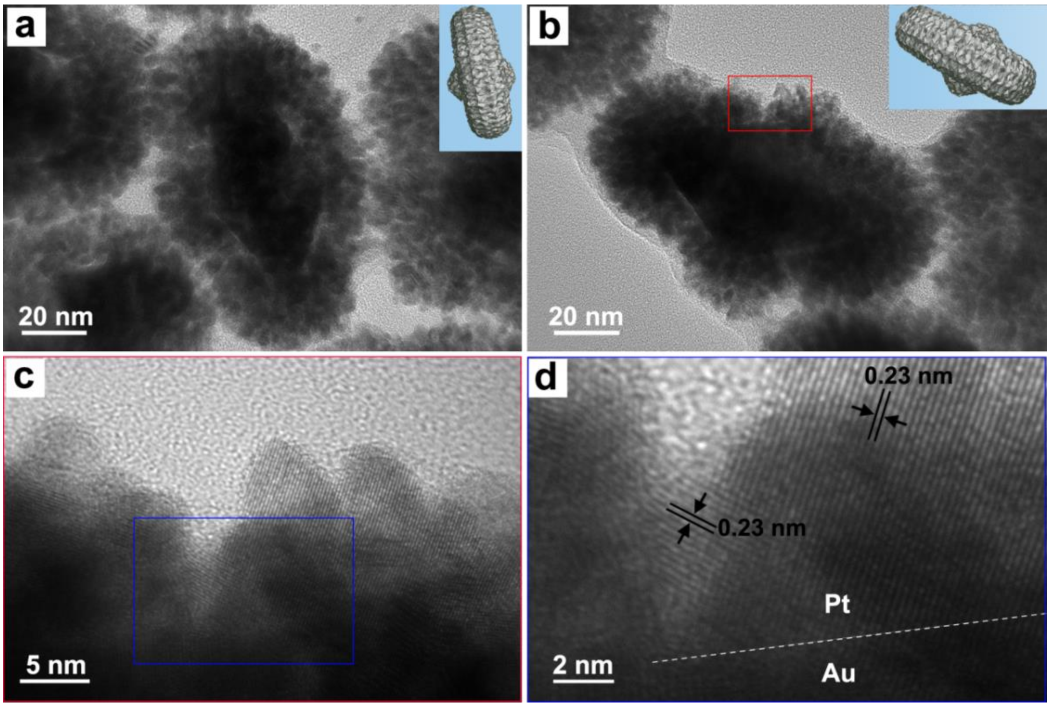


**Supplementary Fig. 9** **a**, **b** HRTEM images of AuND@PtNCs (Pt/Au=2:1 mol/mol). **c** Detailed structure of area marked by the red box in **b**. **d** Detailed structure of area marked by the blue box in **c**. Clearly, high-concentration Pt causes dense and full coating Pt on AuND@PtNCs. The insets in **a** and **b** are corresponding schematic illustrations.

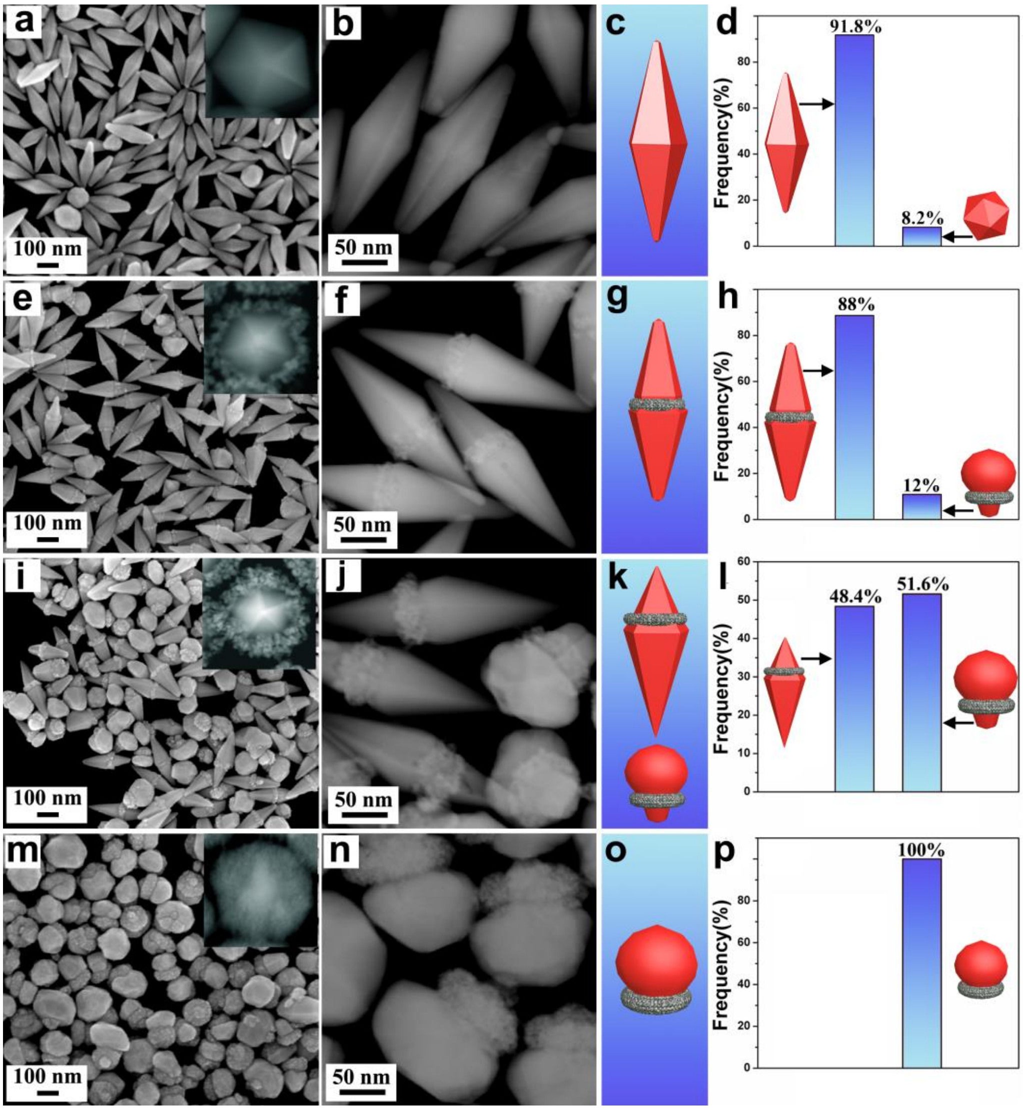


**Supplementary Fig. 10** SEM images, HAADF images, schematic models, and yield diagrams of products prepared through overgrowing Au on AuND@PtNC seeds with different Pt/Au molar ratios: **a**, **b**, **c**, **d** 0; **e**, **f**, **g**, **h** 0.5; **i**, **j**, **k**, **l** 1; **m**, **n**, **o**, **p** 2. The AuNBs prepared using Pt-free AuNDs as seeds are symmetrical along longitudinal direction. However, the AuNB domains resulting from Au growth on AuND@PtNC have Pt-dependent symmetry. For example, compared with AuNB@PtNC nanocrystals shown in **e**, the AuNB@PtNC nanocrystals shown in **i** have lower symmetry. The TEM image in **m** also shows that products with no NB-like shape were formed and increased with the increase of Pt. Similarly, newly-formed two Au domains are different dimensionally. Moreover, the dimension difference increases with Pt, indicating that the evolution of symmetry is mainly related to Pt, regardless of product shape change.

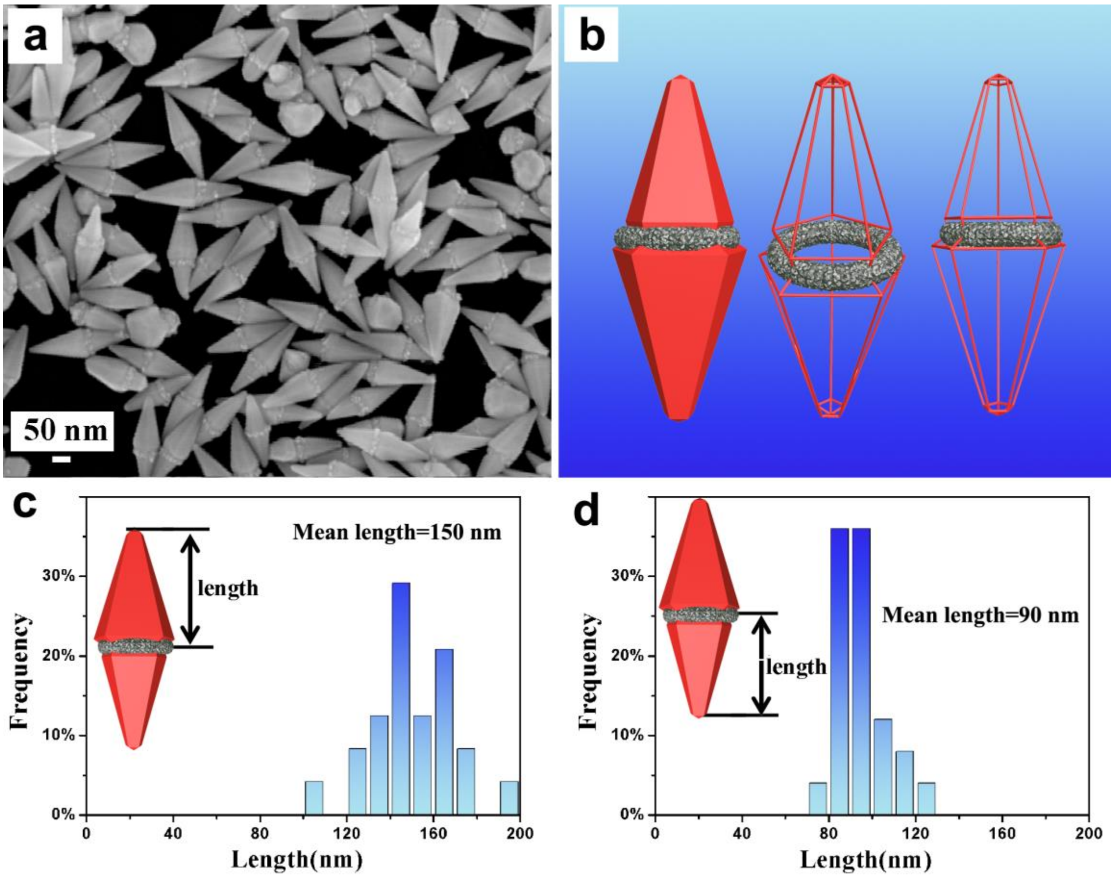


**Supplementary Fig. 11** **a** SEM image, **b** schematic illustration, and **c, d** the length histograms of AuNB@PtNC nanocrystals shown in Supplementary Fig. 10e on two sides. These results demonstrate that the structure is asymmetrical along longitudinal direction.

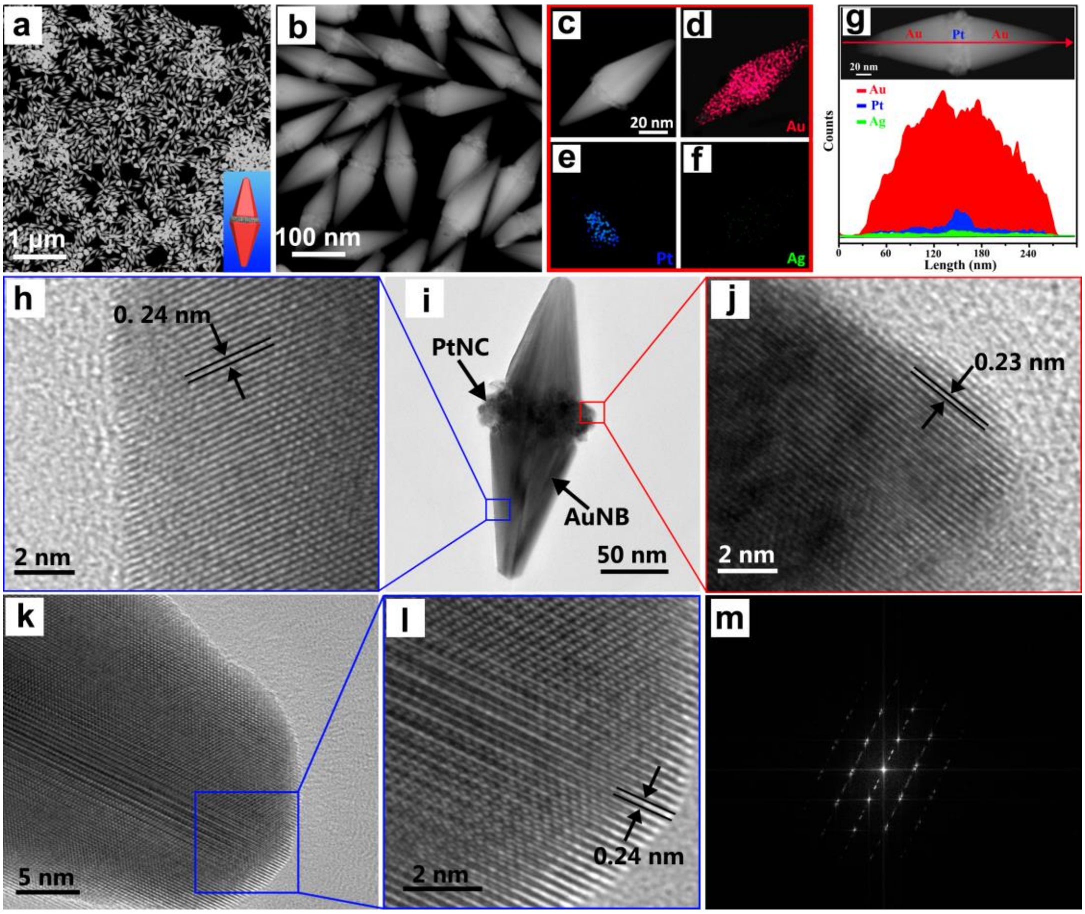


**Supplementary Fig. 12 a**, **b**, **c** HAADF images of AuNB@PtNC nanocrystals. The HAADF image in **a** demonstrates the high yield. **d**, **e**, **f** EDS elemental maps and **g** elemental line profiles of AuNB@PtNC particle. **h**, **i**, **j**, **k**, **l** HRTEM images of one AuNB@PtNC particle. **h** and **j** are the detailed structures of areas marked by the blue and red boxes in **i**, respectively. **l** Detailed structures of area marked by the blue box in **k**. **m** FFT pattern obtained from the HRTEM image in **k**. The HRTEM image in **k** and FFT pattern in **m** show that the crystalline structure of AuNB section in AuNB@PtNC particle is similar to that of AuNB.

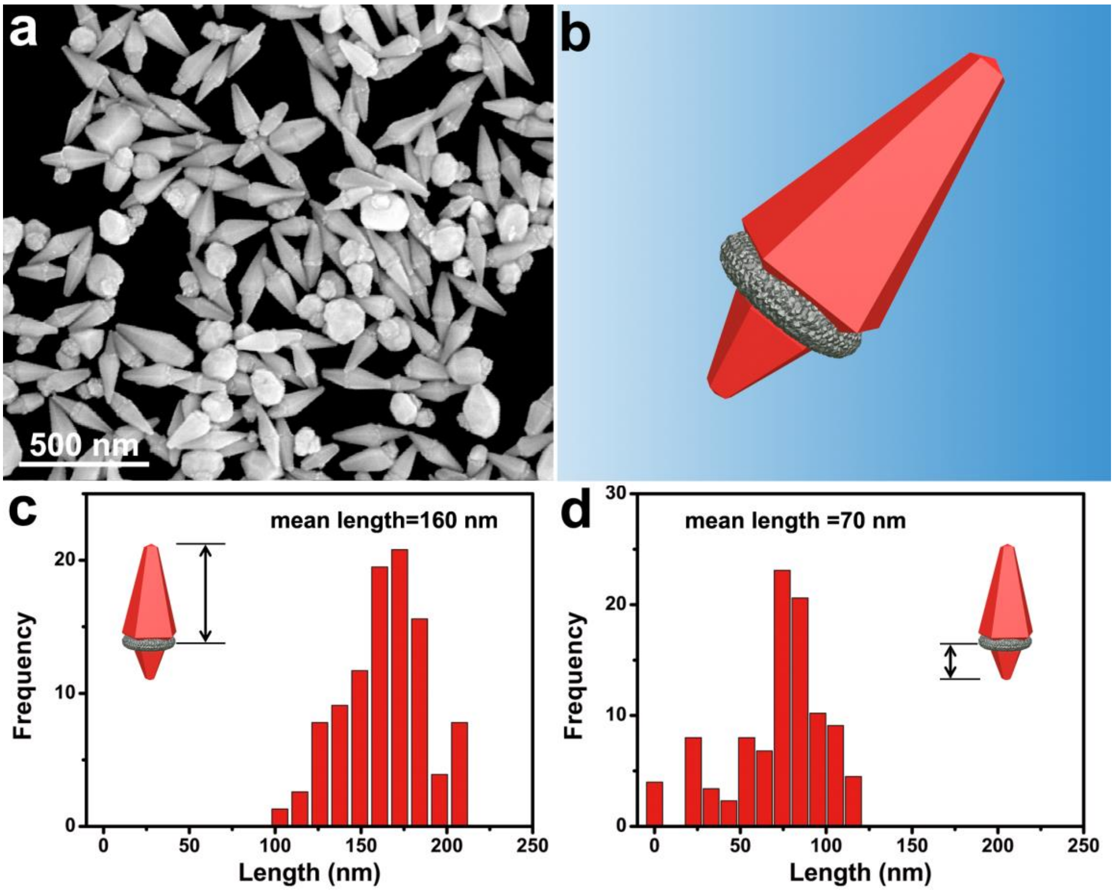


**Supplementary Fig. 13 a** SEM image, **b** schematic illustration, and **c**, **d** the length histograms of AuNB@PtNC nanocrystals shown in Supplementary Fig. 10i on two sides. These results demonstrate that the longitudinal symmetry further decreases with the increase of Pt, compared with the nanocrystals shown in Supplementary Fig. 10e.

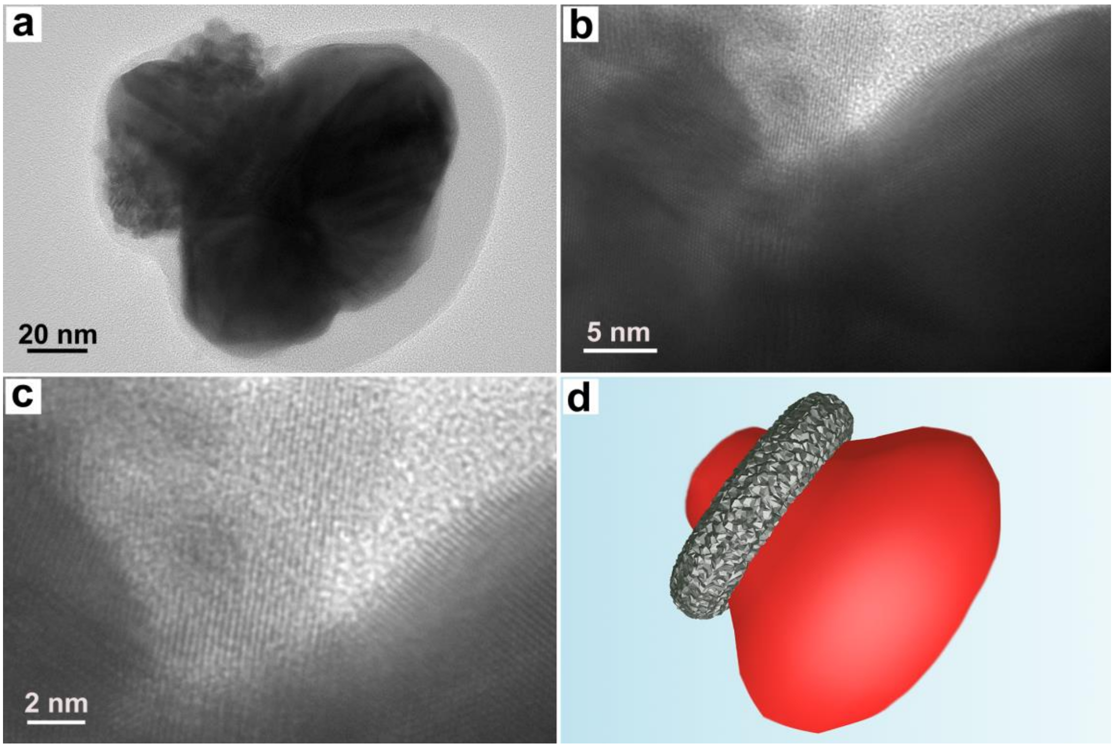


**Supplementary Fig. 14**. **a** TEM image, **b**, **c** HRTEM images, and **d** schematic illustration of one nanocrystal shown in Supplementary Fig. 10i. Clearly, Au domain on one side is much larger than that on another side, indicating that growth symmetry further decreased.

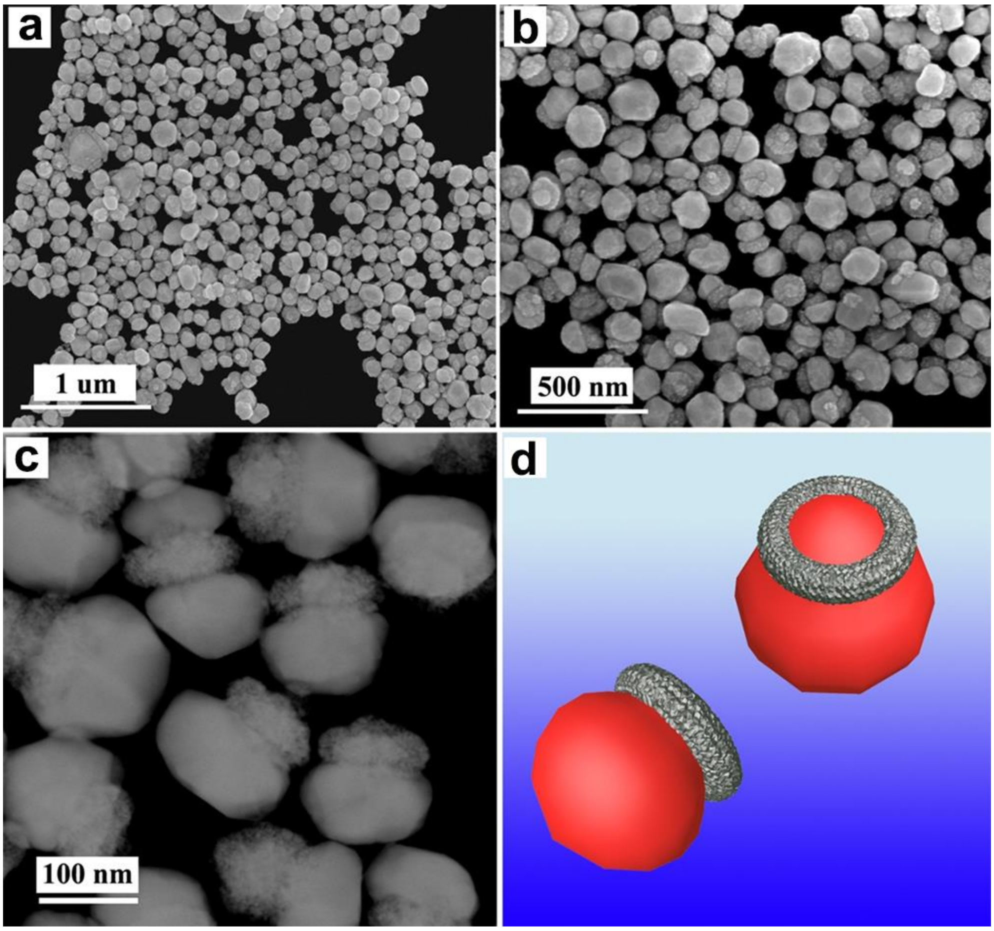


**Supplementary Fig. 15.** **a**, **b** SEM images, **c** HAADF image, and **d** schematic model of Janus-like nanocrystals. They are the same sample with the nanocrystals shown in Supplementary Fig. 10m. High yield is demonstrated by the SEM images in **a** and **b**.

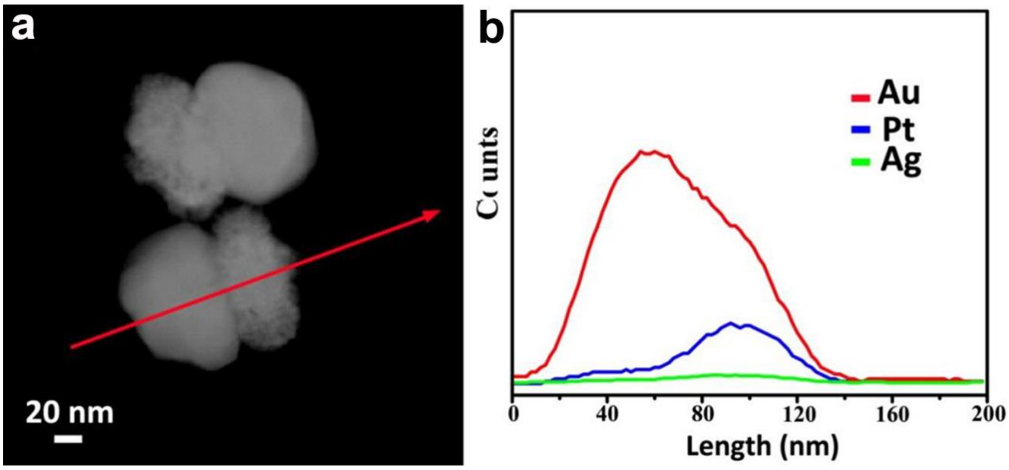


**Supplementary Fig. 16.** **a** HAADF image and of two Janus-like nanocrystals from samples shown in Supplementary Fig. 15. **b** Composition line profiles of a single Janus-like particle in **a** (The Red arrow in **a** indicates the scanning direction).

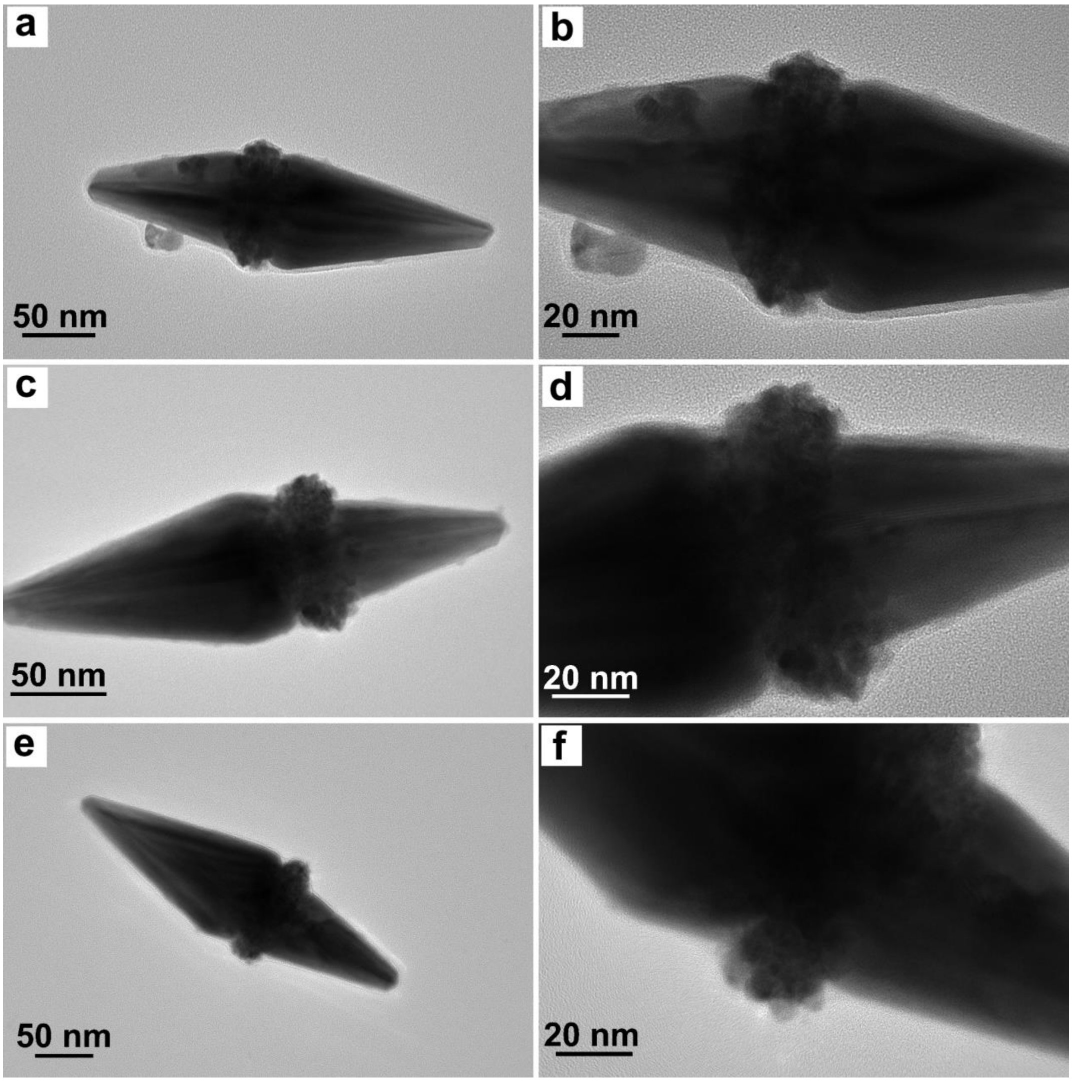


**Supplementary Fig. 17 a**, **b**, **c**, **d**, **e**, **f** HRTEM images of AuNB@PtNC nanocrystals prepared through growing Au on AuND@PtNC (the molar ratio of Pt and Au was 1:1). Clearly, Au growth tried to avoid Pt-rich surface, because large lattice mismatch can lead to high-energy Pt-Au interface.

**Supplementary Note 2. Supplementary discussion on growth mechanism**

This note mainly presents additional discussions on the growth of asymmetrical AuNB@PtNCs nanocrystals.

It is worth discussing how Au adatoms migrate from Pt-rich surface to nucleation sites. Pt deposition on AuND adopted an island-like model,[10, 11] and Pt particles are interconnected to form porous structure (Supplementary Fig. 3-6 and 9). Moreover, the change of surface lattice spacing demonstrates alloying occurred in Pt-Au interface (Fig. 2), so it can be known that Pt and Au atoms both exist on seed surface, as well as Pt islands. Therefore, after Au atoms adsorb on AuND@PtNC surface to form adatoms, they might migrate in various paths dependent on the position they initially adsorb (Fig. 4), for two examples, from {100} facet to {111} facets and then to nucleation site, and from Pt island surface to {100} facets or {111} facets and then to nucleation site.

**Supplementary Note 3. Characterizations of AuNR and AuNB-based nanocrystals**

This note mainly presents the structure characterization of AuNR nanocrystals, AuNR@Pt, AuNB@Pt nanocrystals, and their derivative products.

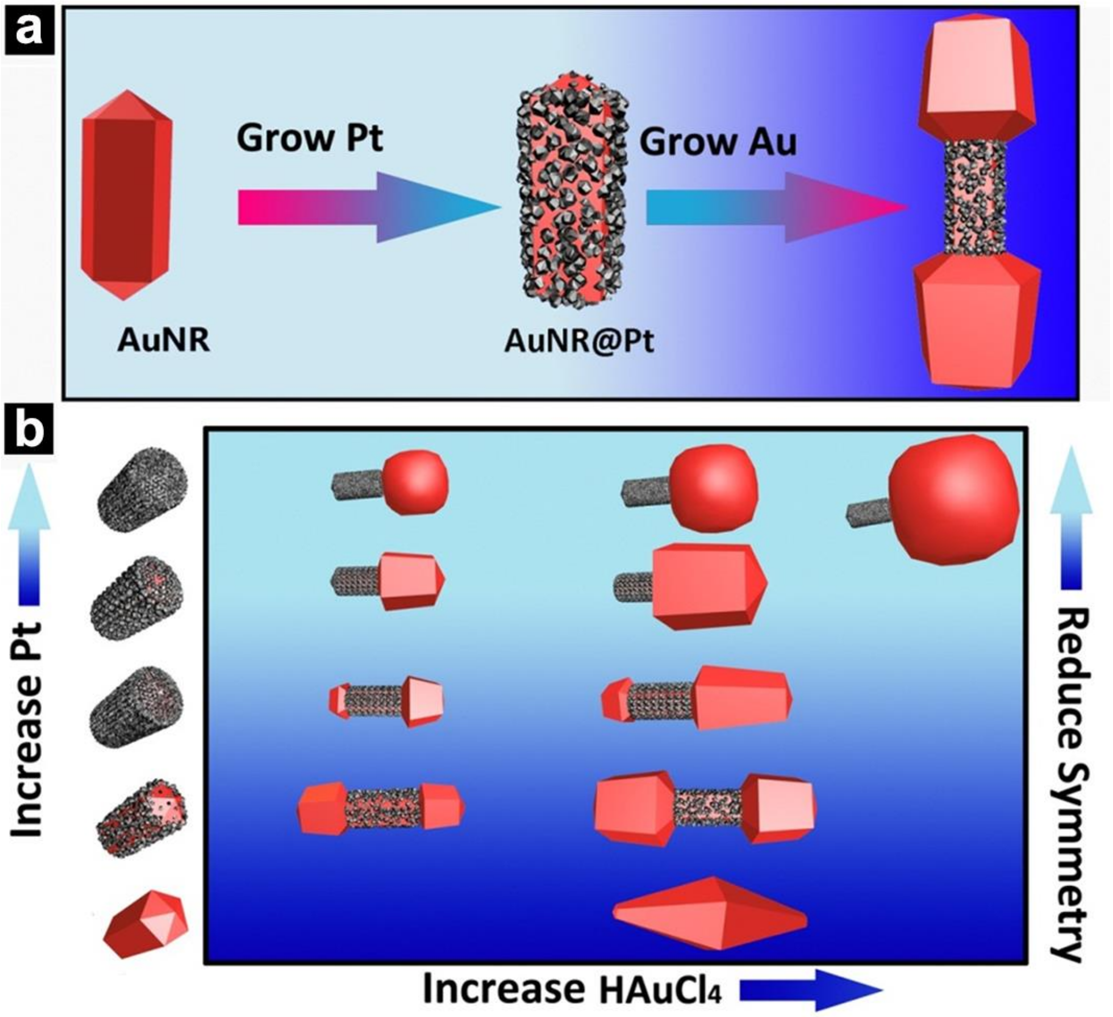


**Supplementary Fig. 18. a** Schematic of preparing AuNR-(AuNR@Pt)-AuNR nanocrystal. **b** Schematic illustration of the products' symmetry change with Pt.

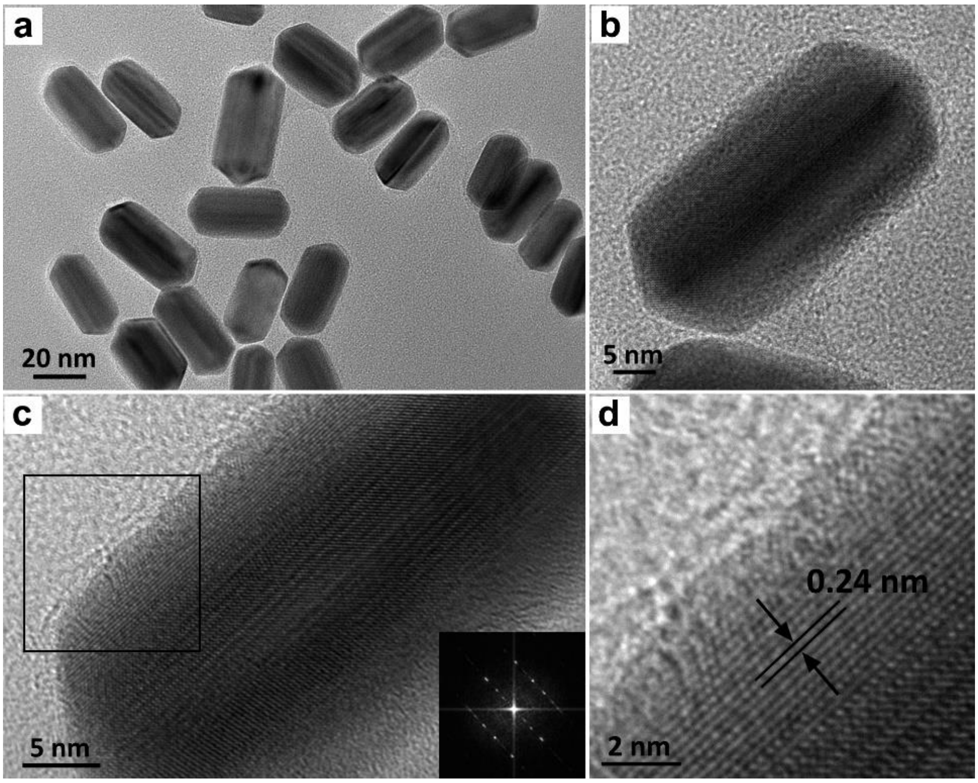


**Supplementary Fig. 19 a** TEM image and **b**, **c**, **d** HRTEM images of AuNRs. **d** Detailed structure of area marked by the black box in **c**. The inset in **c** is corresponding FFT pattern. 0.240 nm in **d** is attributable to the interplanar spacing of Au(111).

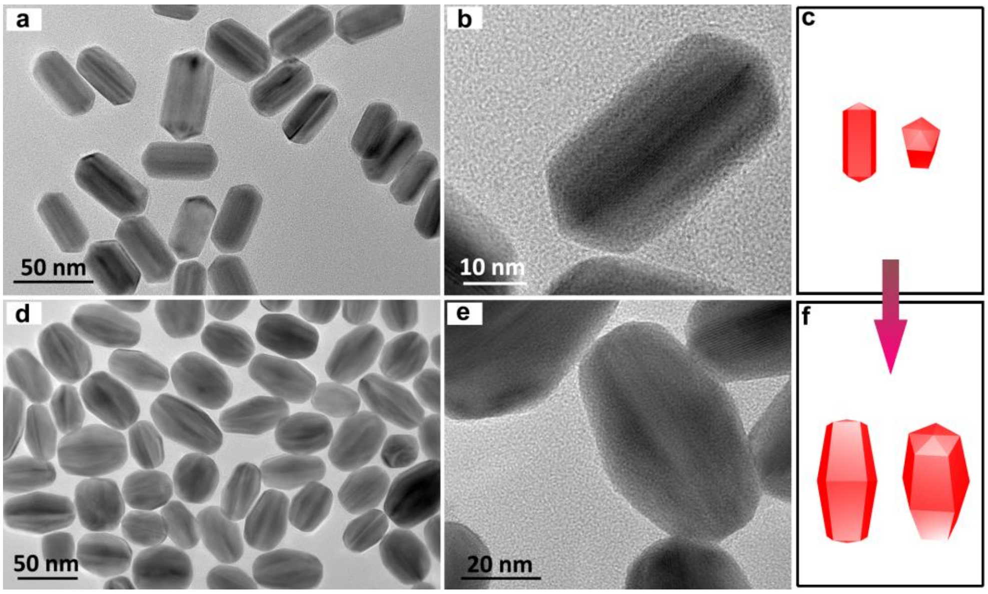


**Supplementary Fig. 20** TEM images, HRTEM images, and schematic sketches of **a**, **b**, **c** AuNR seeds and **d**, **e**, **f** corresponding products prepared through overgrowing Au on AuNR seeds ($HAuCl_4$/Au=4:1 mol/mol). These results indicate that the absence of Pt facilitates symmetrical growth rather than asymmetrical growth.

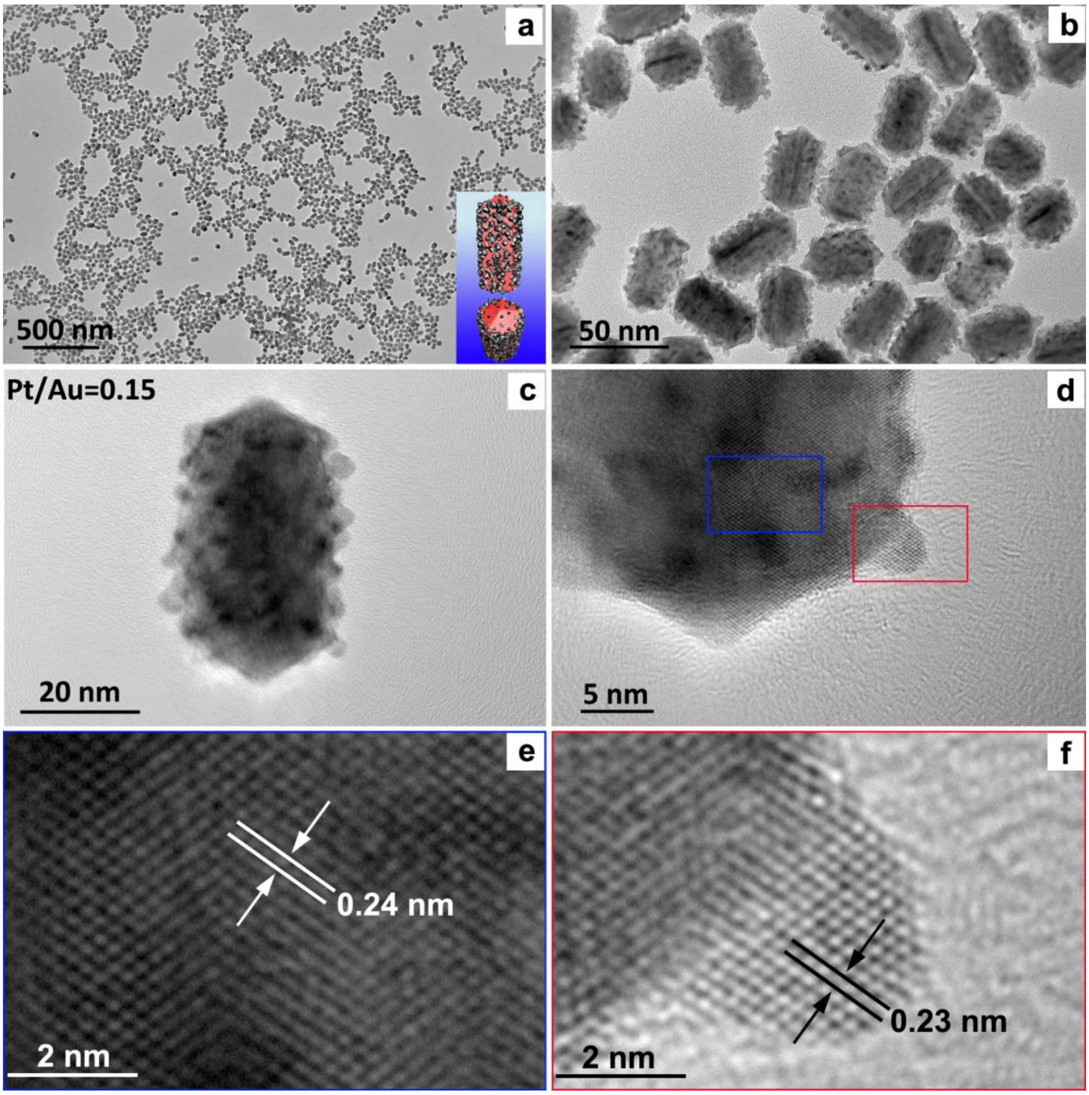


**Supplementary Fig. 21** **a**, **b** TEM images, **c**, **d** HRTEM images of AuNR@Pt nanocrystals (Pt/Au=0.15:1 mol/mol), **e** the detailed structure of area marked by the blue box in **d**, and **f** the detailed structure of area marked by the red box in **d**.

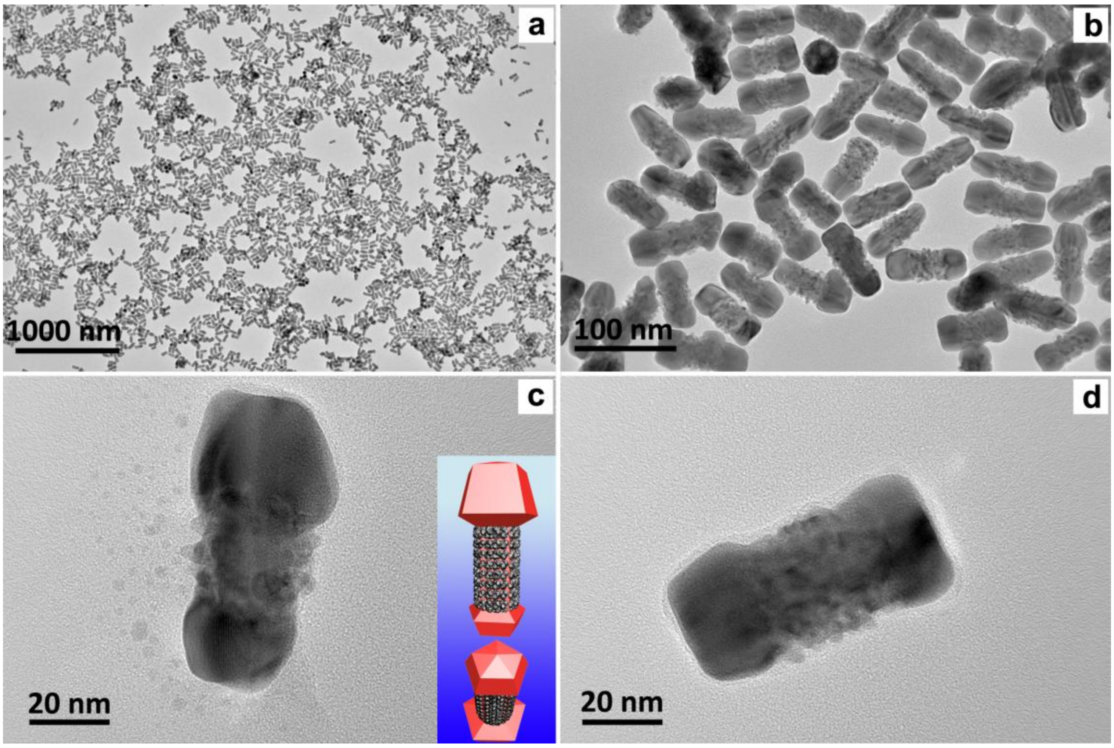


**Supplementary Fig. 22** **a**, **b** TEM images and **c**, **d** HRTEM images of products prepared through overgrowing Au on seeds shown in Supplementary Fig. 21 ($HAuCl_4$/Au=2:1 mol/mol). The inset in **c** is the schematic illustration.

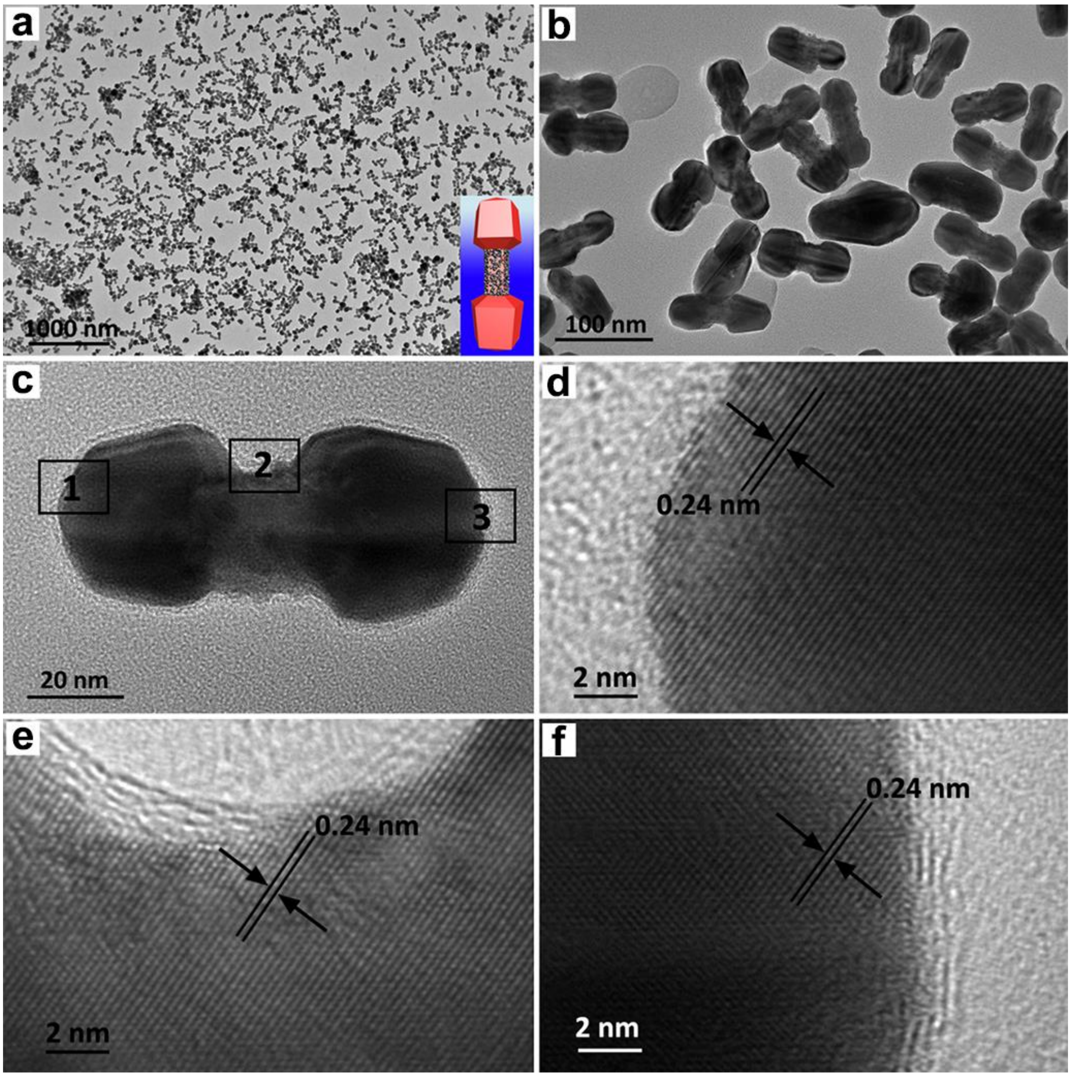


**Supplementary Fig. 23** **a**, **b** TEM images and **c**, **d**, **e**, **f** HRTEM images of products prepared through overgrowing Au on seeds shown in Supplementary Fig. 21 ($HAuCl_4$/Au=4:1 mol/mol). The inset in **a** is the schematic of nanocrystal. **d**, **e**, and **f** are the detailed structures of areas marked by the black boxes 1, 2, and 3 in **c**, respectively. 0.24 nm in **d**, **e**, and **f** are assigned to the interplanar spacing of Au(111). The TEM result in **e** indicates that small amount of Pt has no significant effects on the lattice spacing of Au. These TEM images (Supplementary Fig. 22 and 23) show that the dimension of newly formed Au domain can be tuned through controlling the amount of Au precursor.

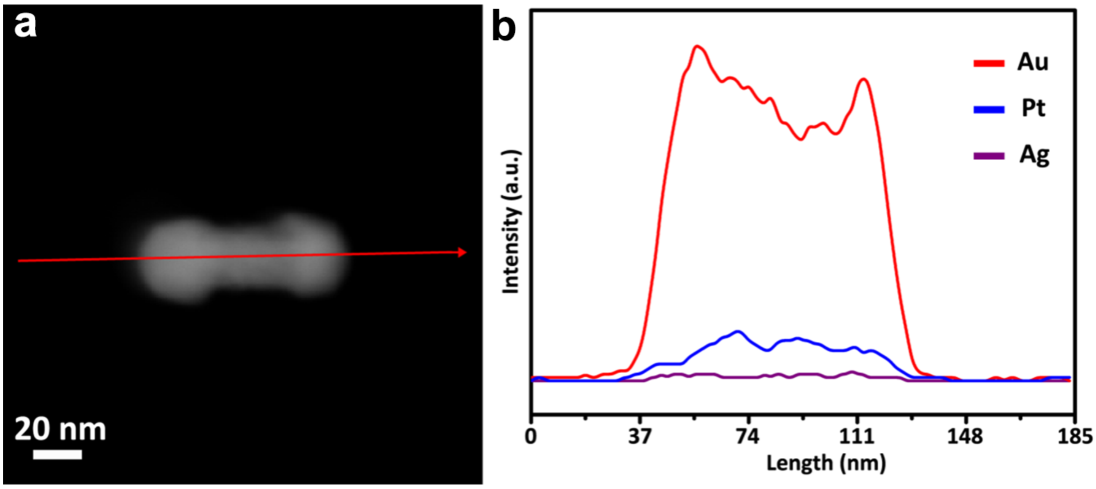


**Supplementary Fig. 24** **a** HAADF image and **b** composition line profiles of an individual nanocrystal from products shown in Supplementary Fig. 23a. The red arrow in **a** indicates the direction of composition line scanning.

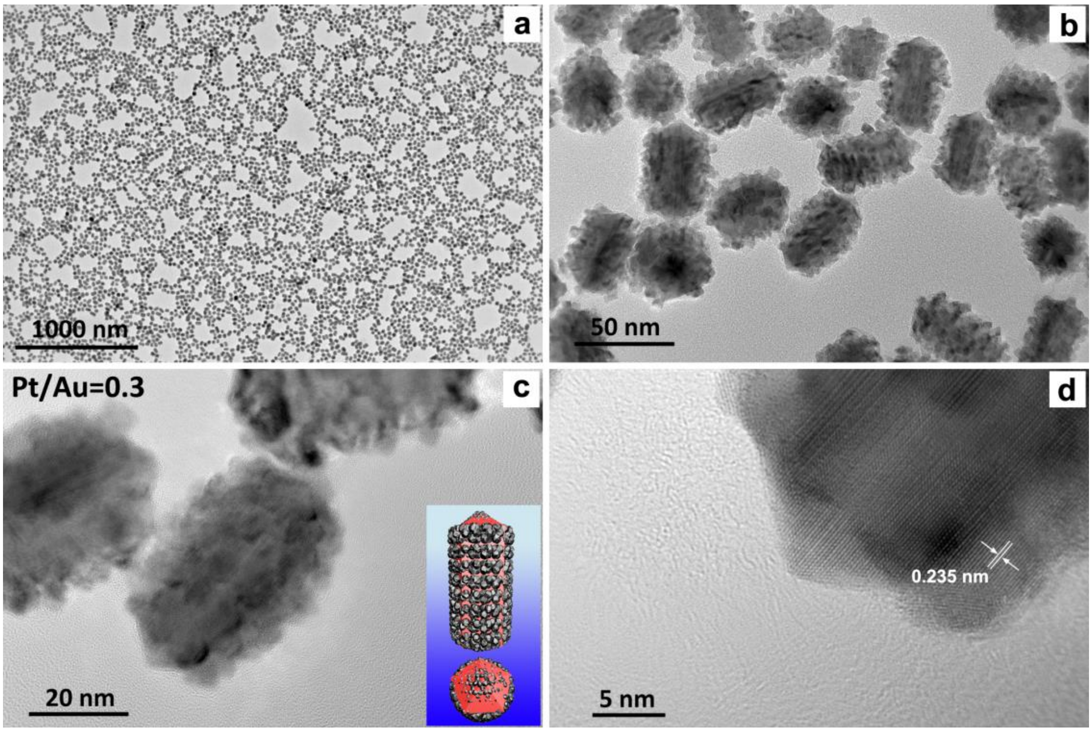


**Supplementary Fig. 25** **a**, **b** TEM images and **c**, **d** HRTEM images of AuNR@Pt nanocrystals (Pt/Au=0.3:1 mol/mol). These HRTEM images demonstrate the preferential growth of Pt on {100} facet of AuNRs. The inset in **c** is the schematic illustration. Because Pt deposition change the surface lattice feature, 0.235 nm lattice spacing in **d** is smaller than that of Au(111).

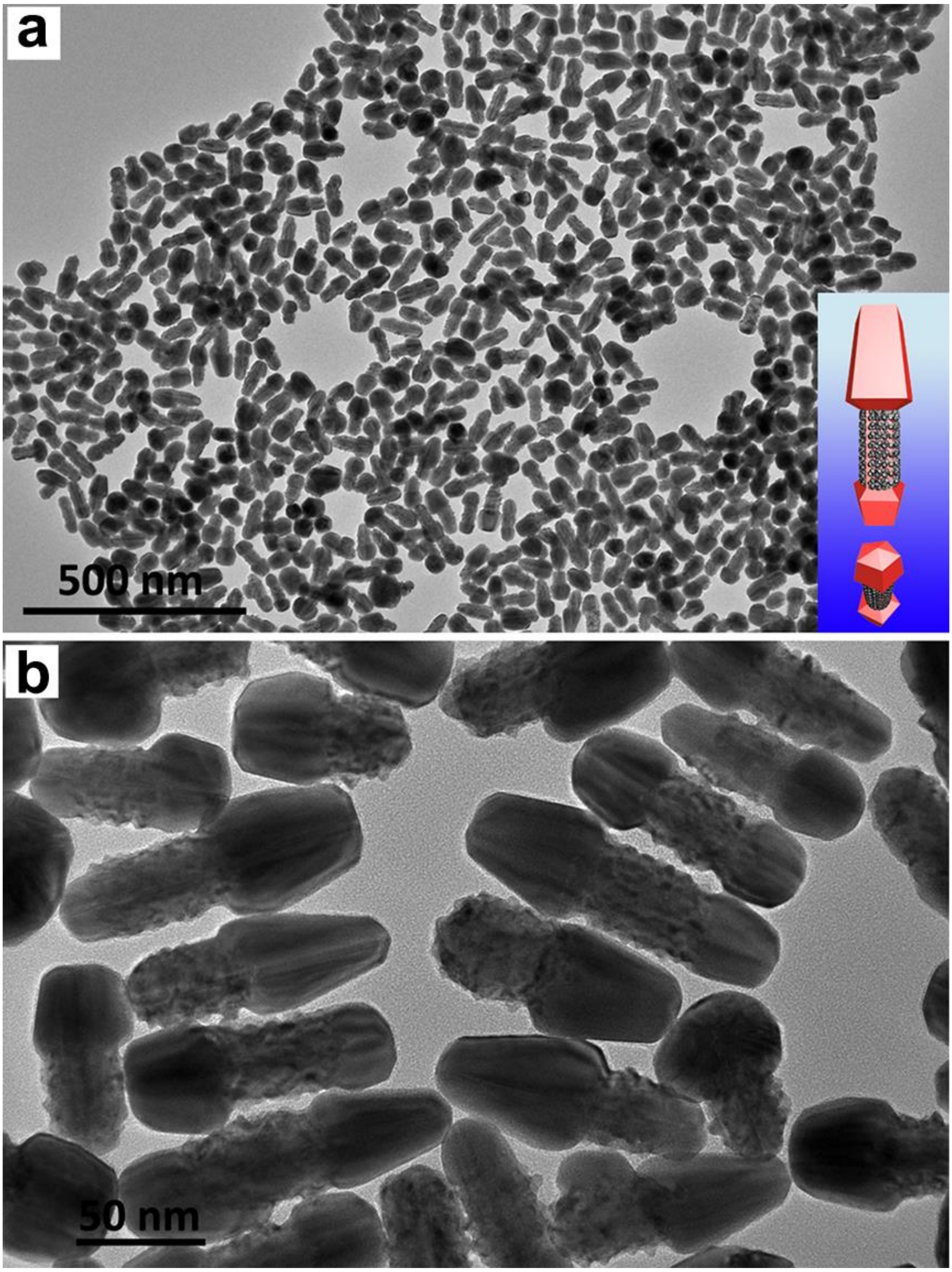


**Supplementary Fig. 26 a**, **b** TEM images of products prepared through overgrowing Au on seeds shown in Supplementary Fig. 25 ($HAuCl_4$/Au=4:1 mol/mol). The inset in **a** is the schematic illustration.

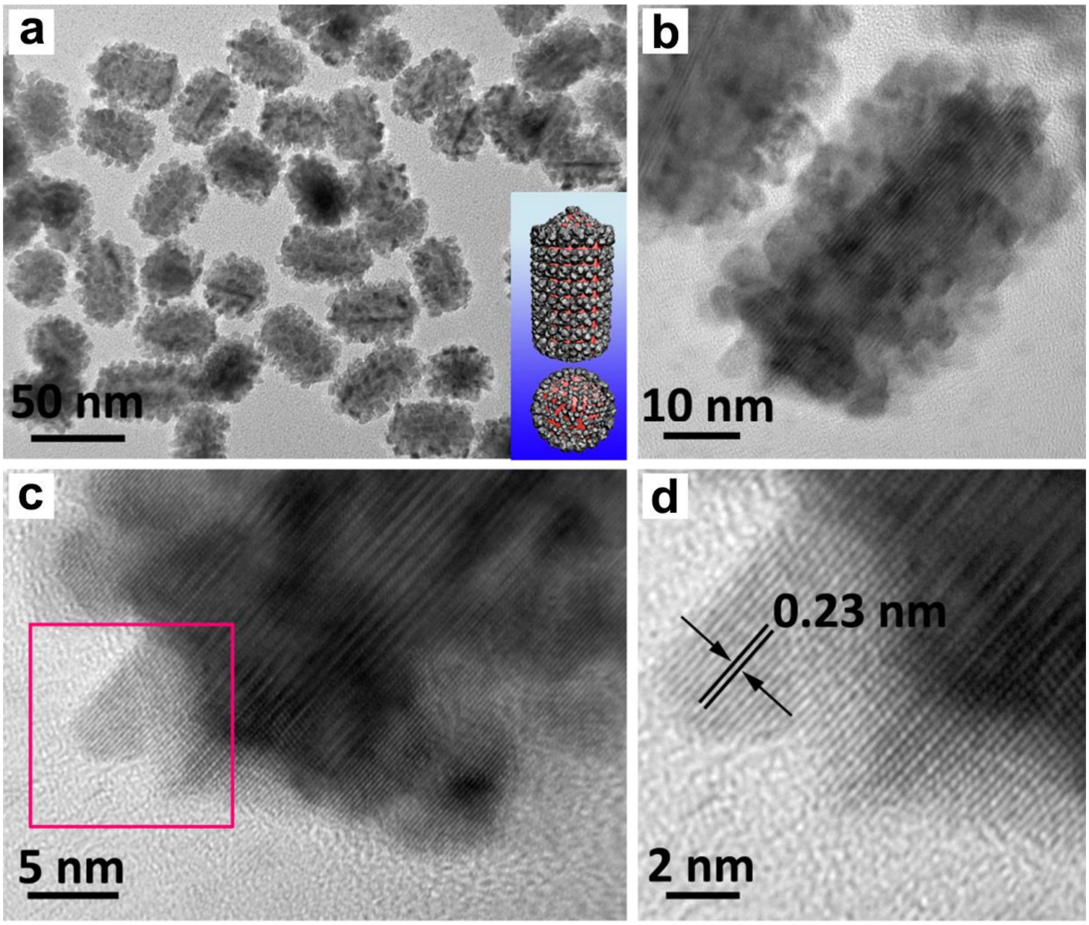


**Supplementary Fig. 27** **a**, **b** TEM images and **c**, **d** HRTEM images of AuNR@Pt nanocrystals (Pt/Au=0.5:1 mol/mol). **d** Detailed structure of area marked by the red box in **c**. The inset in **a** is the schematic illustration. 0.23 nm in **d** is assigned to the interplanar spacing of Pt(111), implying that Pt atoms are nucleated on the {111} facets of AuNR when feeding more Pt.

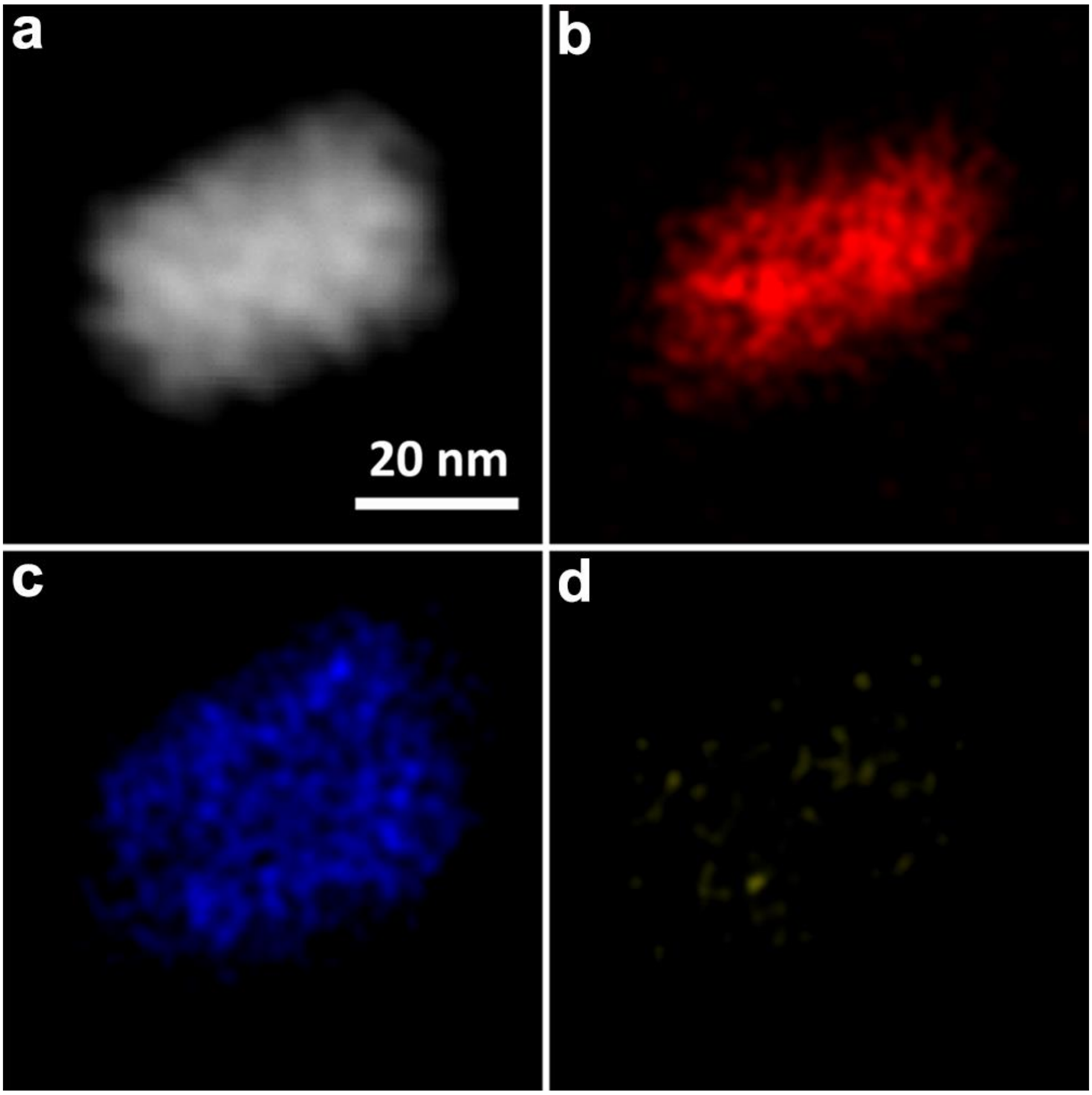


**Supplementary Fig. 28 a** HAADF image and **b**, **c**, **d** elemental maps of an individual AuNR@Pt nanocrystal from products shown in Supplementary Fig. 27.

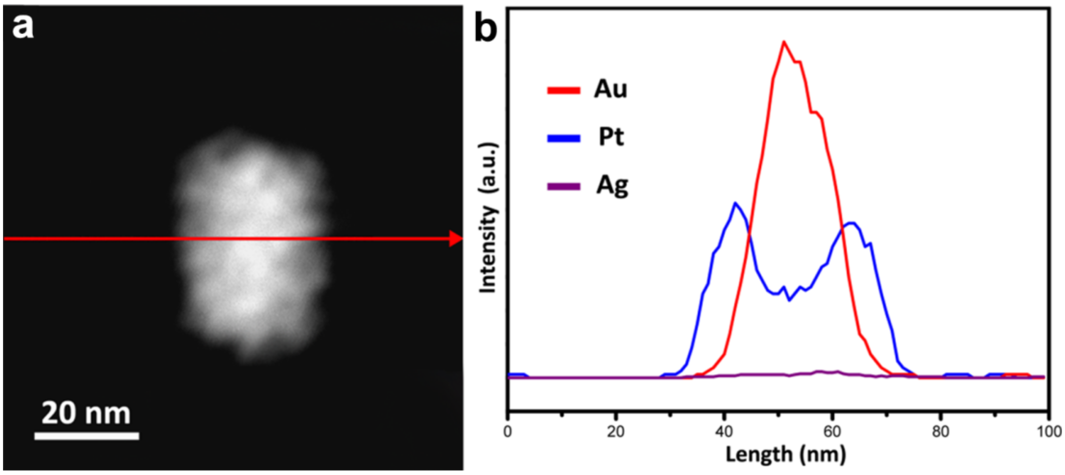


**Supplementary Fig. 29 a** HAADF image and **b** composition line profiles of an individual AuNR@Pt nanocrystal from samples shown in Supplementary Fig. 28. The red arrow in **a** indicates the direction of composition line scanning.

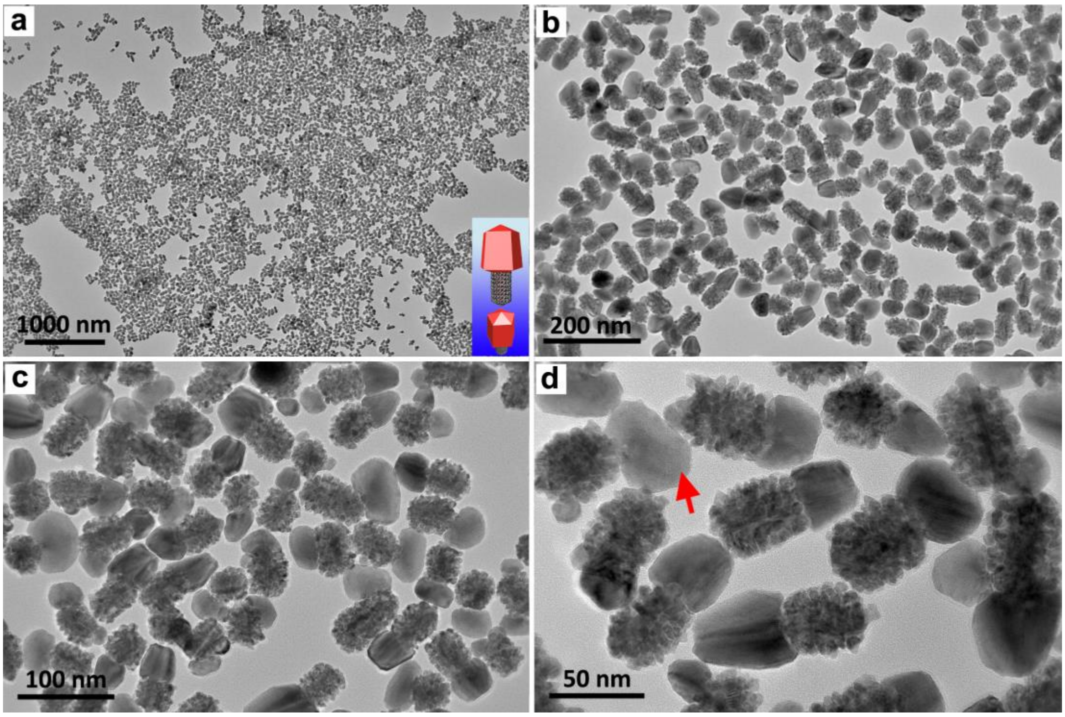


**Supplementary Fig. 30 a**, **b**, **c** TEM images and **d** HRTEM image of products prepared through overgrowing Au on seeds shown in Supplementary Fig. 27 ($HAuCl_4$/Au=2:1 mol/mol). Not all newly-formed Au domains are five-fold twinned and some of them are single crystalline, because dense Pt coating makes AuNRs seeds lost the ability to induce growth. The red arrow in **d** marks the single crystalline Au domain.

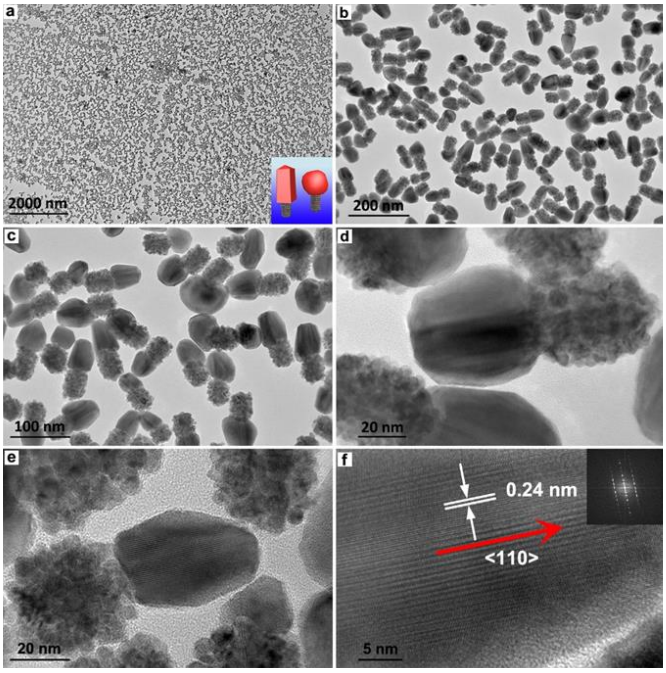


**Supplementary Fig. 31** **a**, **b**, **c** TEM images and **d**, **e**, **f** HRTEM images of products prepared through overgrowing Au on seeds shown in Supplementary Fig. 27 ($HAuCl_4$/Au=4:1 mol/mol). The red arrow in **f** indicates the penta-fold axis. The inset in **f** is the corresponding FFT pattern of the HRTEM image in **f**.

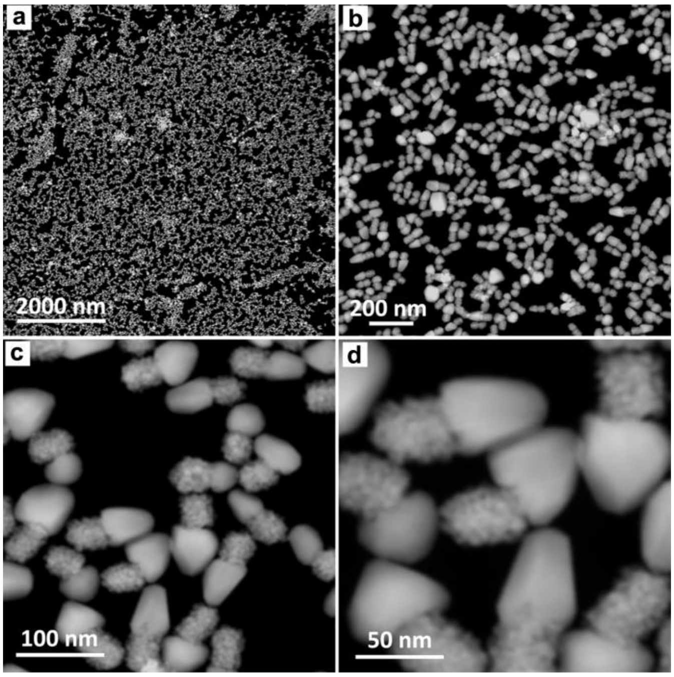


**Supplementary Fig. 32** **a**, **b**, **c**, **d** HAADF images of products shown in Supplementary Fig. 31.

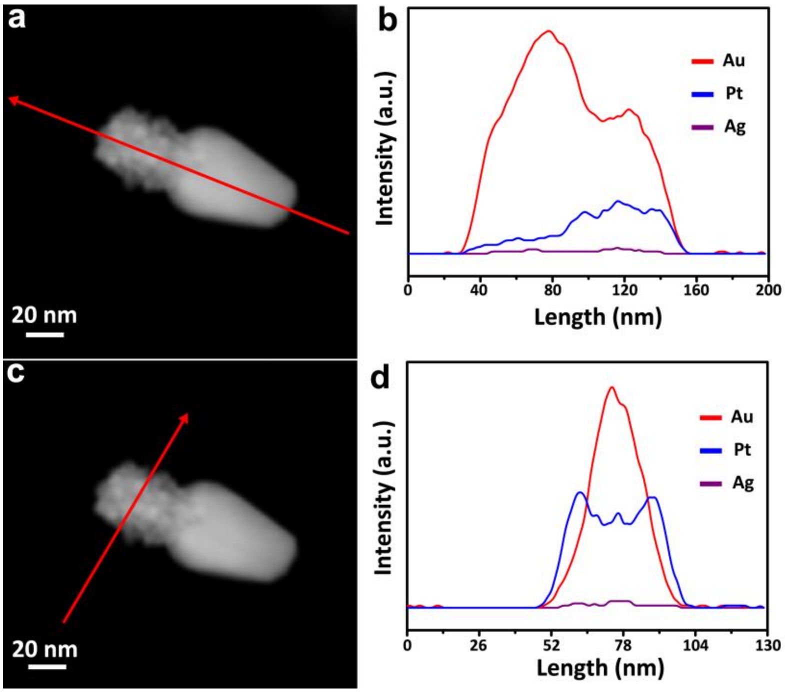


**Supplementary Fig. 33** **a** HAADF image of an individual nanocrystal from products shown in Supplementary Fig. 31 and **b** composition line profiles along the scanning direction marked by the red arrow in **a**. **c** HAADF image of the same nanocrystal and **d** composition line profiles along the scanning direction marked by the red arrow in **c**.

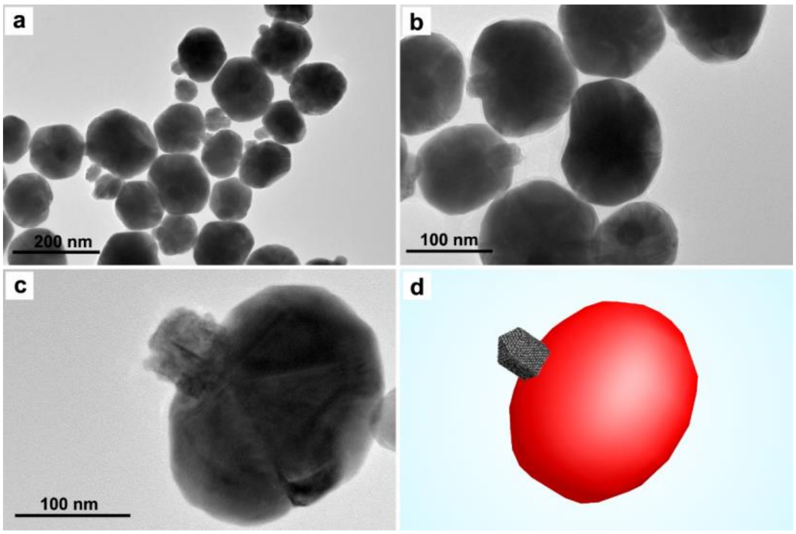


**Supplementary Fig. 34** **a**, **b**, **c** TEM images and **d** schematic illustration of nanocrystals prepared through overgrowing Au on seeds shown in Supplementary Fig. 27 ($HAuCl_4$/Au=12:1 mol/mol). High concentration of Au precursor causes inhomogeneous growth, creating a broad size distribution, but growth mode had no changes.

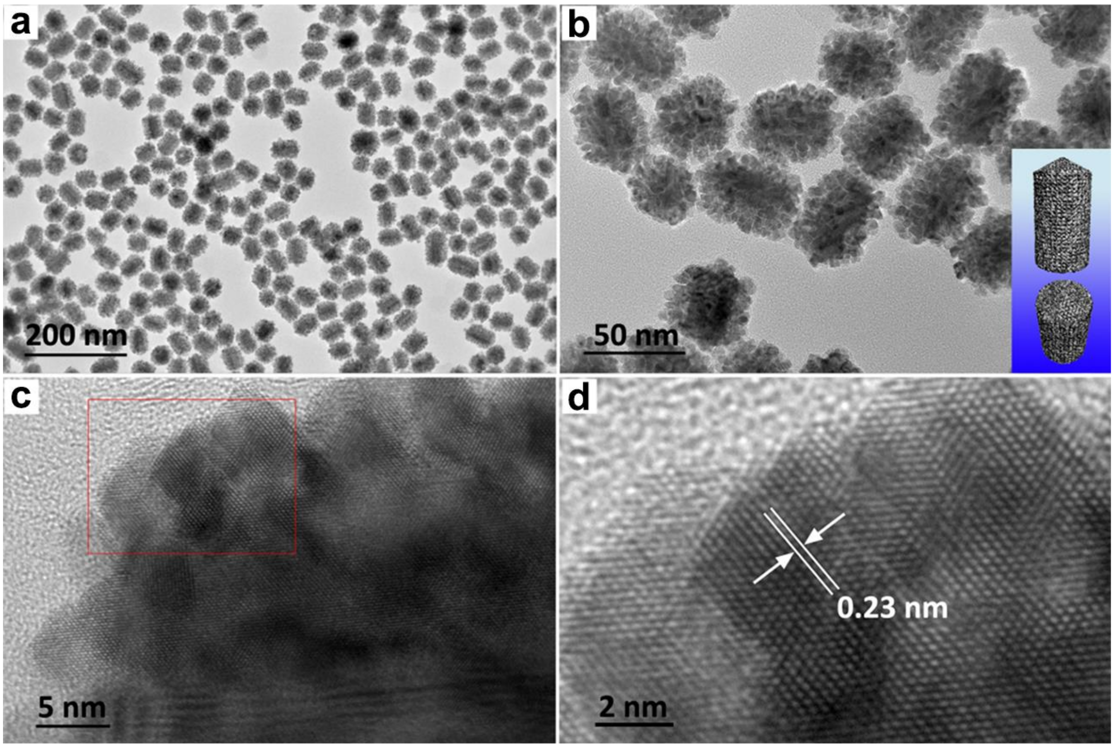


**Supplementary Fig. 35 a**, **b** TEM images and **c**, **d** HRTEM images AuNR@Pt (Pt/Au=1:1 mol/mol). **d** Detailed structure of area marked by the red box in **c**. The inset in **b** is the schematic illustration. 0.23 nm in **d** is attributed to the interplanar spacing of Pt(111).

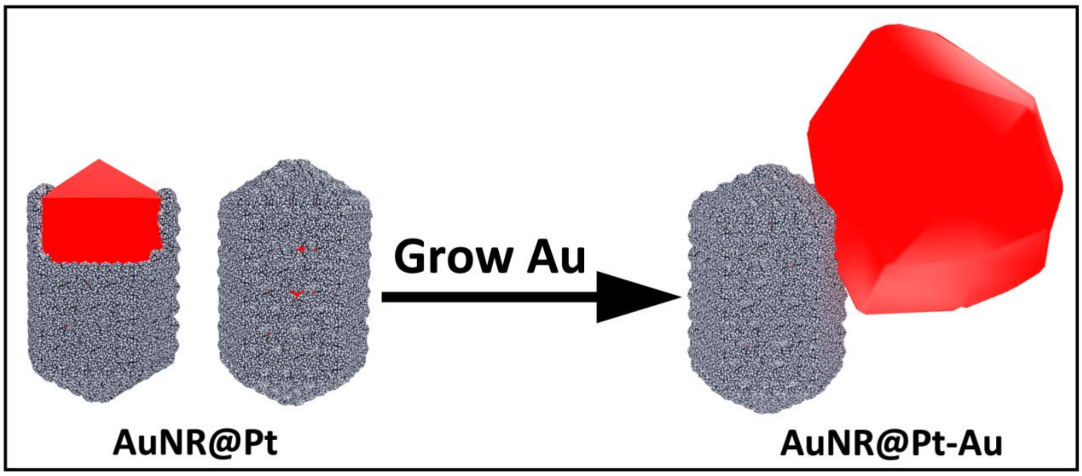


**Supplementary Fig. 36.** Schematic illustration of growth on AuNR@Pt nanocrystal.

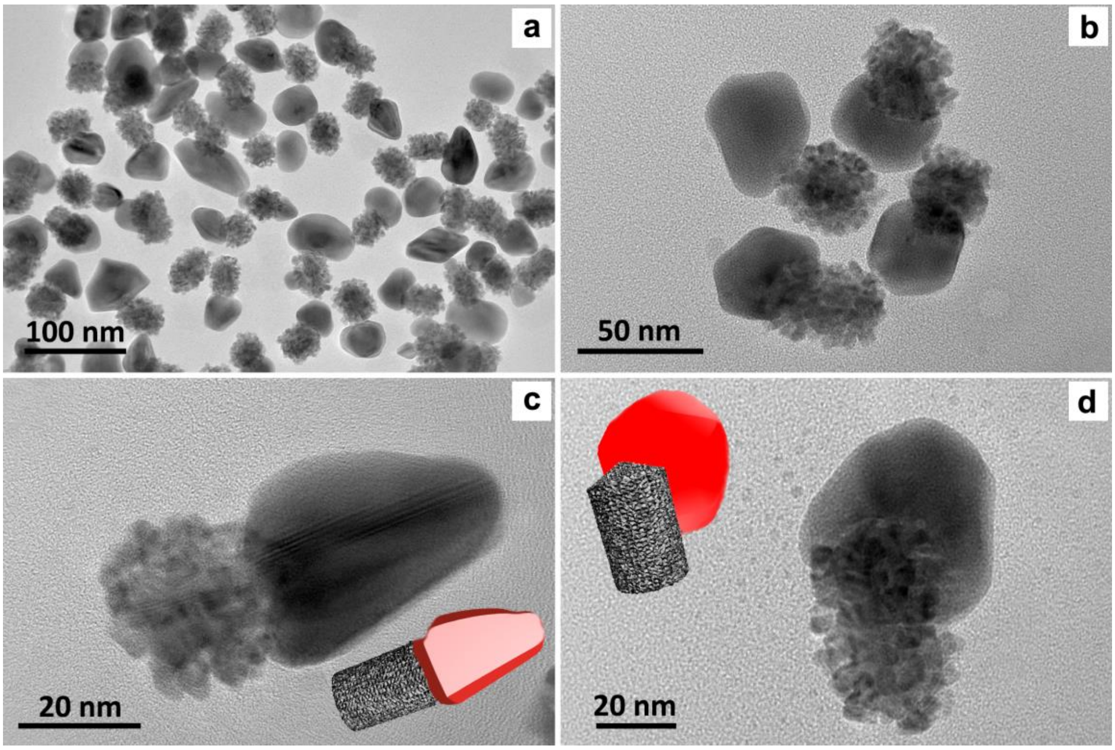


**Supplementary Fig. 37 a** TEM image and **b**, **c**, **d** HRTEM images of nanocrystals prepared through overgrowing Au on seeds shown in Supplementary Fig. 35 ($HAuCl_4$/Au=2:1 mol/mol). The insets in **c** and **d** are the schematic illustrations. The AuNRs in AuNR@Pt seeds are almost completely coated by Pt (Supplementary Fig. 35), and therefore, their five-fold twinned crystalline structures have no significant effect on the growth of Au. As a result, most of newly-formed Au nanocrystals are even single crystalline; meanwhile, Au growth occurred randomly surrounding AuNR@Pt seeds instead of only along <110>.

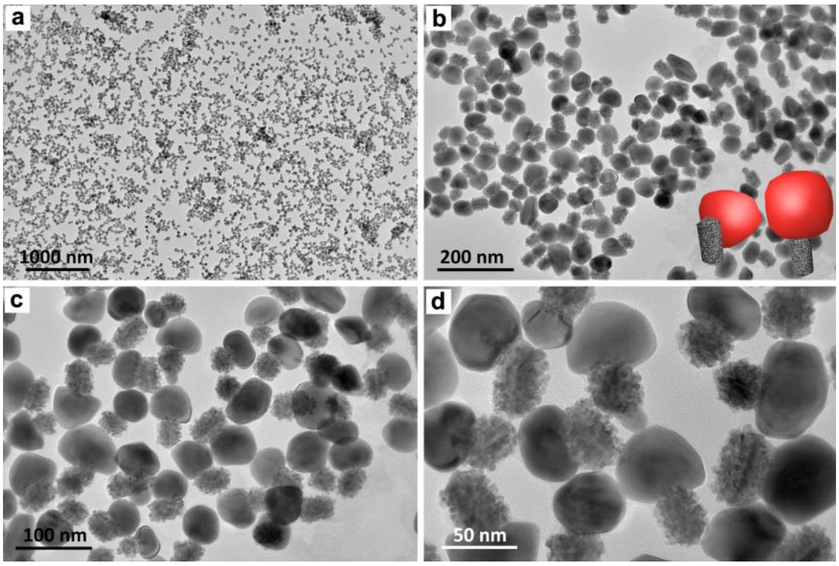


**Supplementary Fig. 38 a**, **b**, **c** TEM images and **d** HRTEM images of nanocrystals prepared through overgrowing Au on seeds shown in Supplementary Fig. 35 ($HAuCl_4$/Au=4:1 mol/mol). The inset in **b** is the schematic illustration.

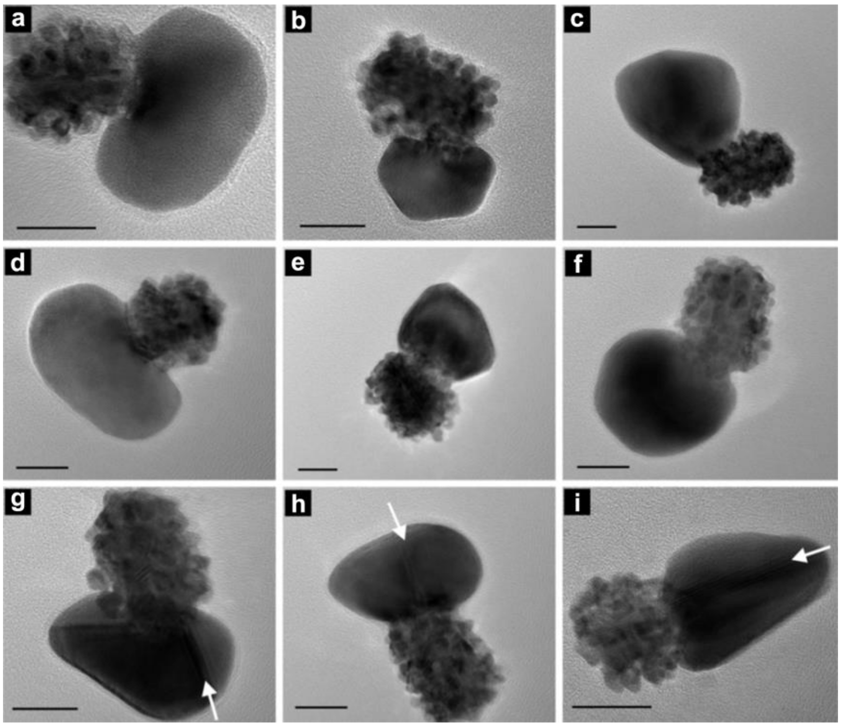


**Supplementary Fig. 39** HRTEM images of some nanocrystals from products shown in Supplementary Fig. 38: **a**, **b**, **c**, **d**, **e**, **f** single crystalline particles (It can be seen that Au growth occurred on the side of seeds in some cases); **g**, **h**, **i** multiple twinned particles (The arrows in **g**, **h**, and **i** mark the twinned boundaries). All scale bars are 20 nm.

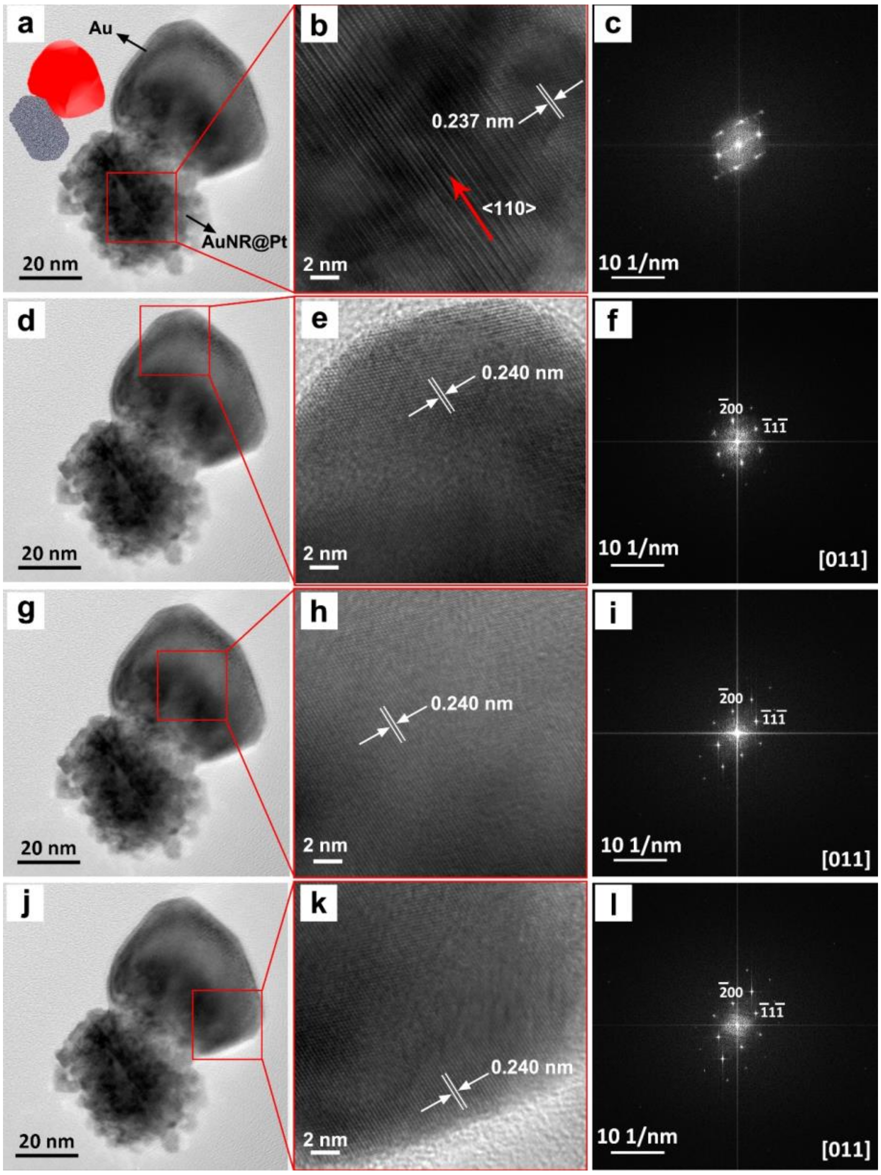


**Supplementary Fig. 40 a** TEM image of an individual nanocrystal (from products shown in Supplementary Fig. 38), **b** HRTEM image of the area marked by the red box in **a**, and **c** FFT pattern obtained on HRTEM result in **b**. **d** TEM image of the same nanocrystal, **e** HRTEM image of area marked by the red box in **d**, and **f** FFT pattern obtained on HRTEM result in **e**. **g** TEM image of the same nanocrystal, **h** HRTEM image of area marked by the red box in **g**, and **i** FFT pattern obtained on HRTEM result in **h**. **j** TEM image of the same nanocrystal, **k** HRTEM image of area marked by the red box in **j**, and **l** FFT pattern obtained on HRTEM result in **k**. These HRTEM images and corresponding FFT patterns in three areas of the newly-formed Au domain demonstrate that no twinned interface is present there and it is single crystalline. The inset in **a** is the corresponding schematic illustration.

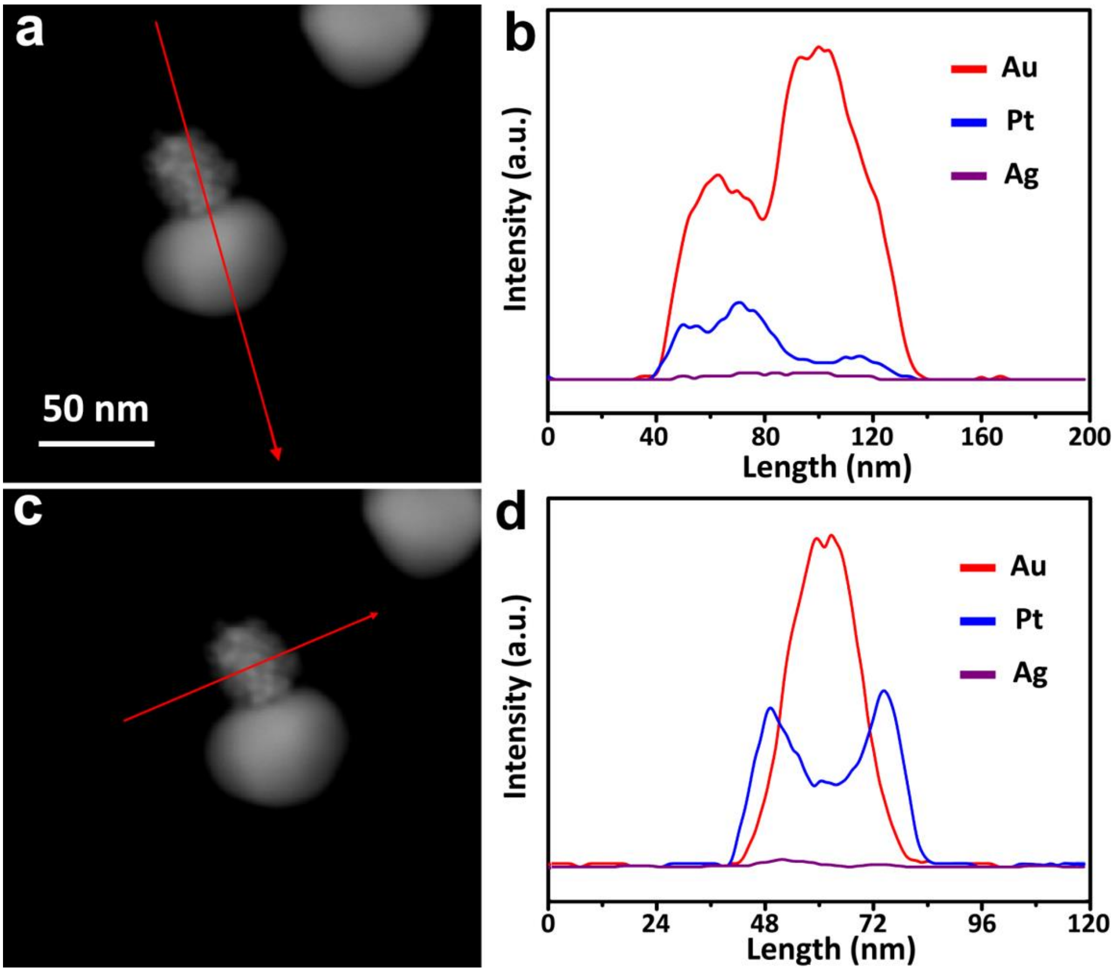


**Supplementary Fig. 41 a** HAADF image of an individual nanocrystal from products shown in Supplementary Fig. 38 and **b** composition line profiles along the scanning direction marked by the red arrow in **a**. **c** HAADF image of the same nanocrystal and **d** composition line profiles along the scanning direction marked by the red arrow in **c**.

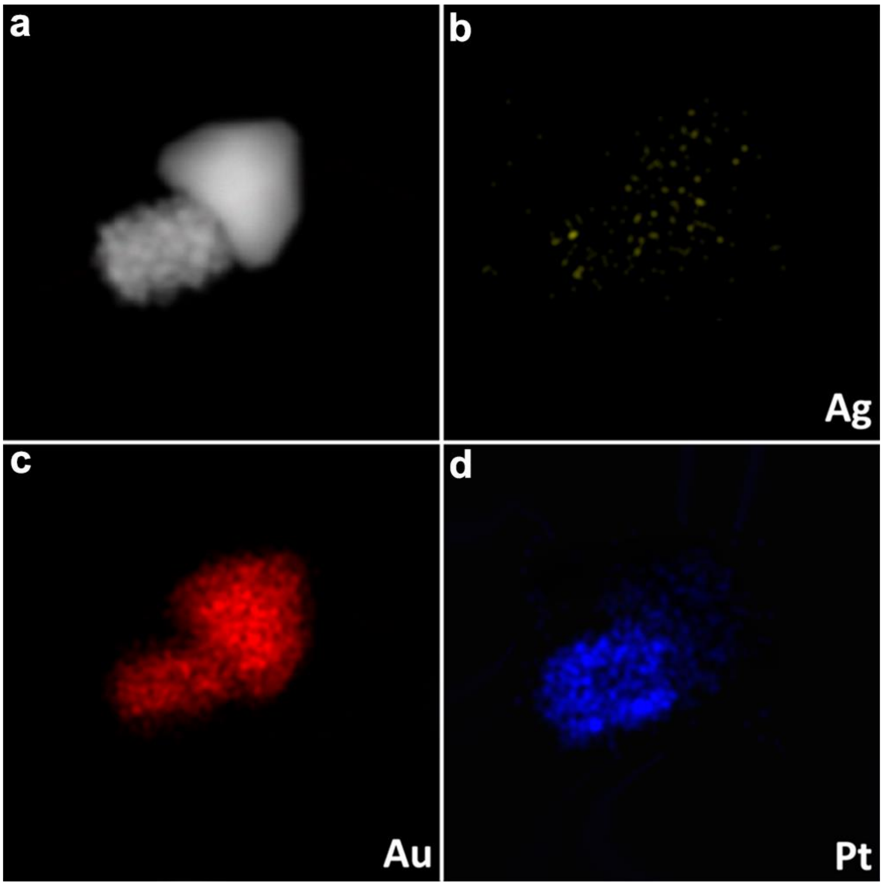


**Supplementary Fig. 42** **a** HAADF image and **b**, **c**, **d** elemental maps of an individual nanocrystal from products shown in Supplementary Fig. 38.

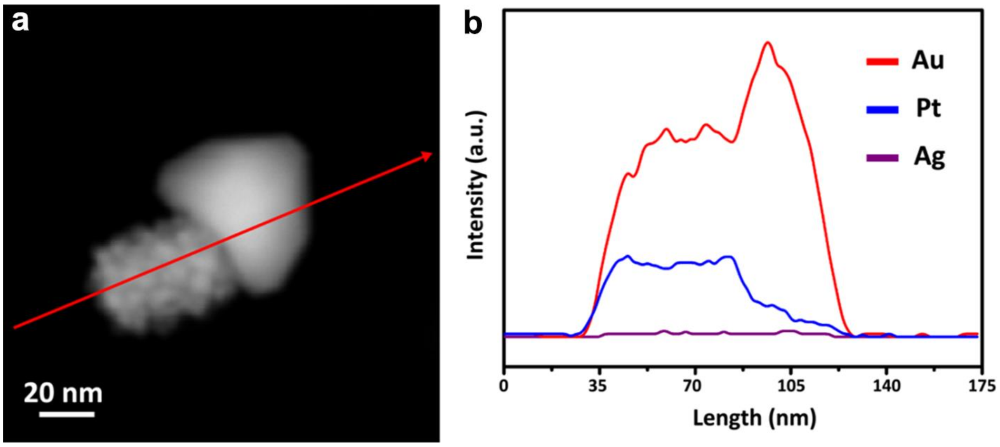


**Supplementary Fig. 43** **a** HAADF image and **b** composition line profiles of the nanocrystal shown in Supplementary Fig. 42. The red arrow in **a** indicates the direction of composition line scanning.

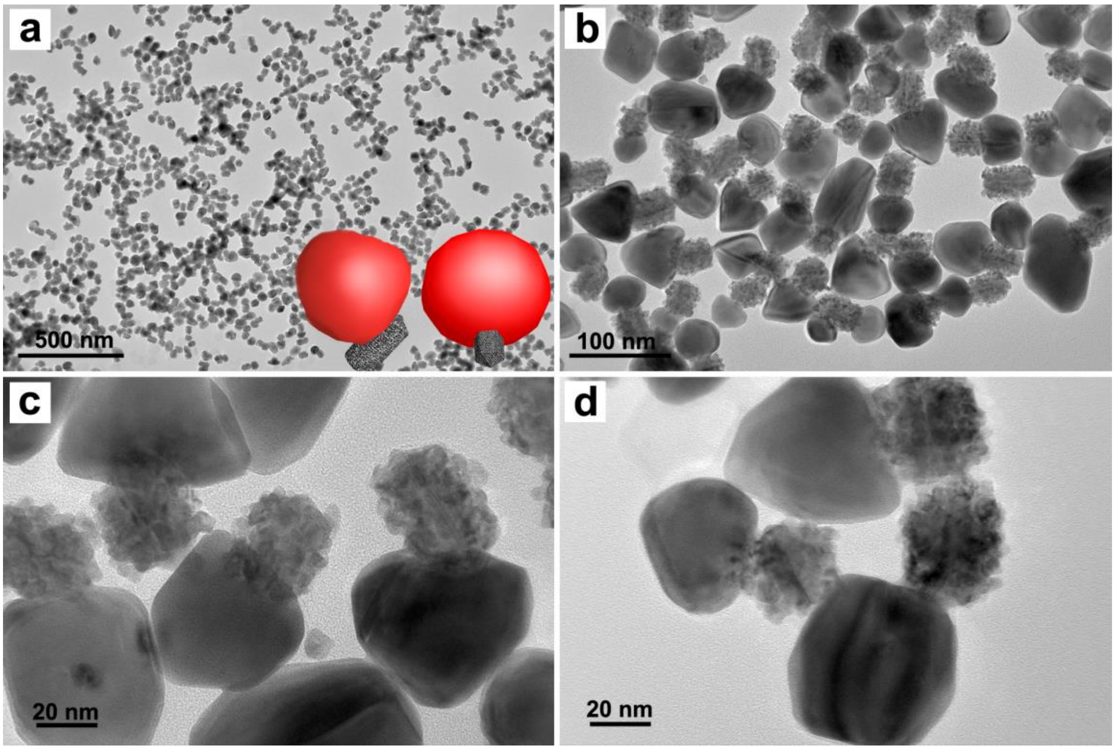


**Supplementary Fig. 44 a**, **b** TEM images and **c**, **d** HRTEM images of nanocrystals prepared through overgrowing Au on seeds shown in Supplementary Fig. 35 ($HAuCl_4$/Au=7:1 mol/mol). The inset in **a** is the schematic illustration.

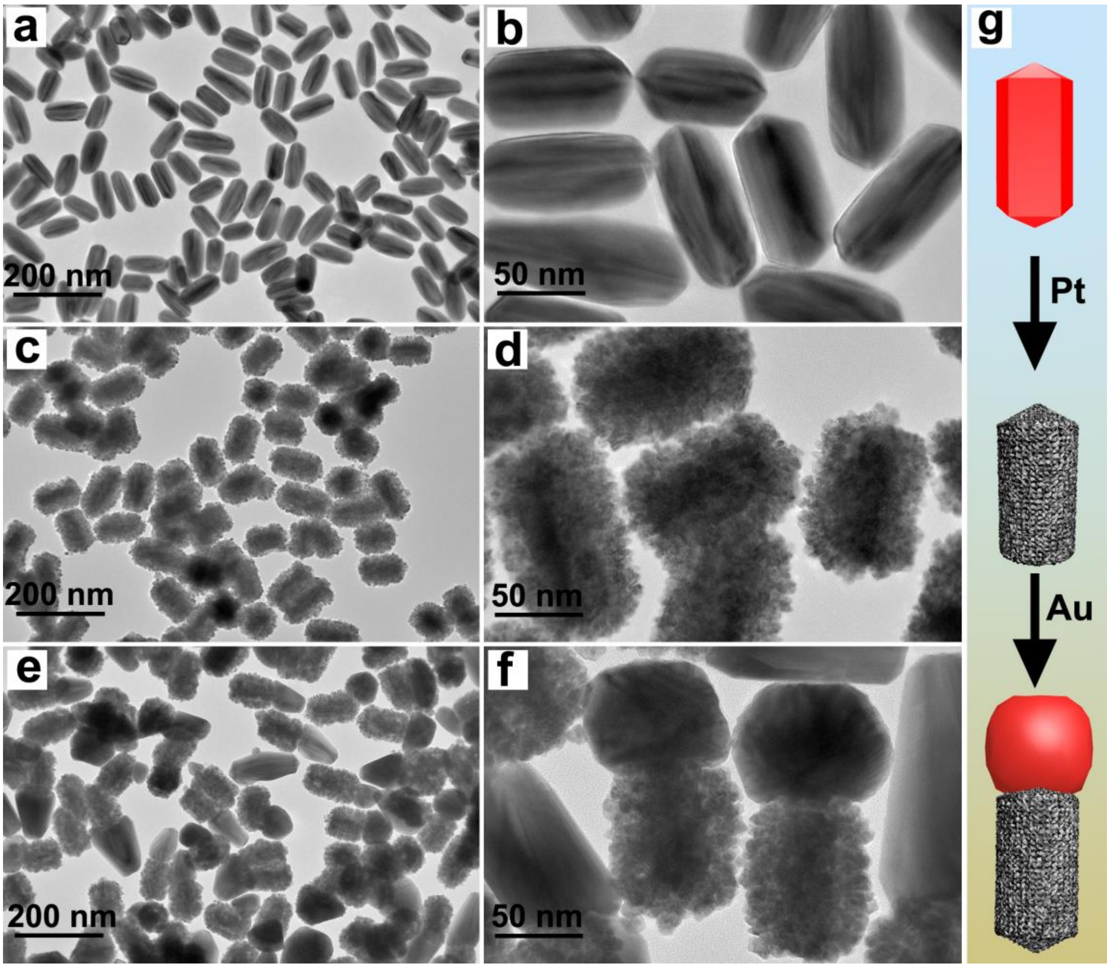


**Supplementary Fig. 45** TEM images of **a**, **b** AuNRs (mean transverse length and longitudinal length are 50 nm and 100 nm, respectively), **c**, **d** AuNR@Pt nanocrystals prepared through overgrowing Pt on the AuNRs (Pt/Au=1:1 mol/mol), and **e**, **f** Janus-like nanocrystals prepared through overgrowing Au on the AuNR@Pt ($HAuCl_4$/Au=4:1 mol/mol). **g** Schematic illustration of growth. The used AuNRs are significantly larger than the AuNRs shown in Supplementary Fig. 19, indicating that Pt-dependent growth is independent of the size of AuNRs.

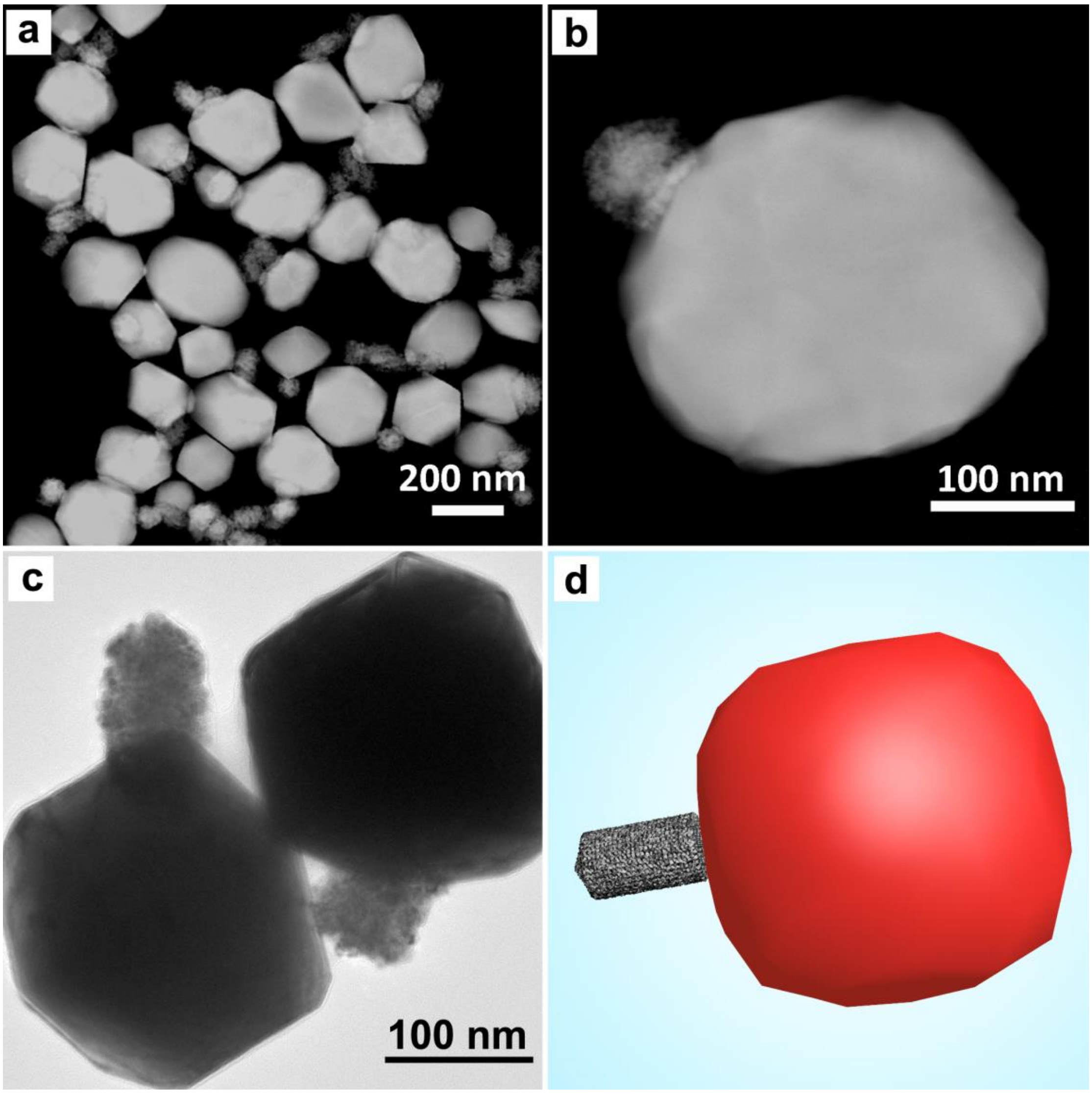


**Supplementary Fig. 46 a, b** HAADF images, **c** TEM image, and **d** schematic illustration of nanocrystals prepared through overgrowing Au on particles shown in Supplementary Fig. 45c ($HAuCl_4$/Au=12:1 mol/mol). These newly-formed Au domains have approximately 200-nm diameter much larger than that shown in Supplementary Fig. 45e, demonstrating that their dimension is tunable within a large size range.

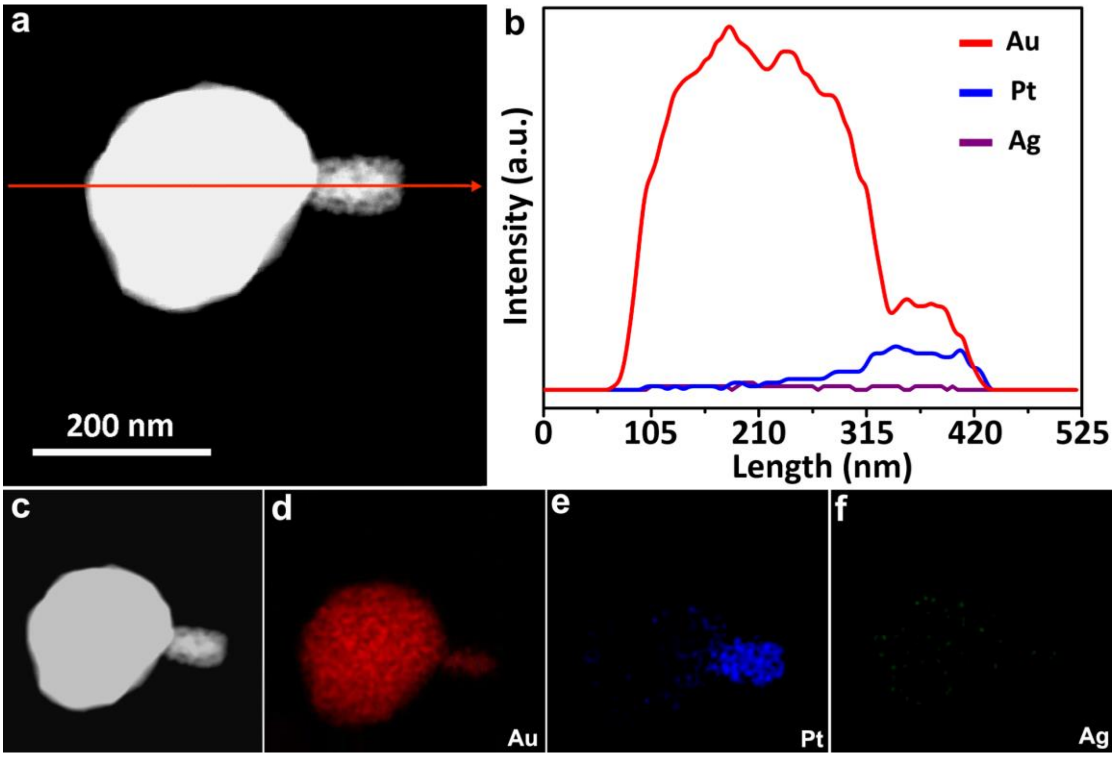


**Supplementary Fig. 47** **a** HAADF image, **b** composition line profiles, and **c, d, e, f** elemental maps of an individual nanocrystal shown in Supplementary Fig. 46. The red arrow in **a** indicates the direction of composition line scanning.

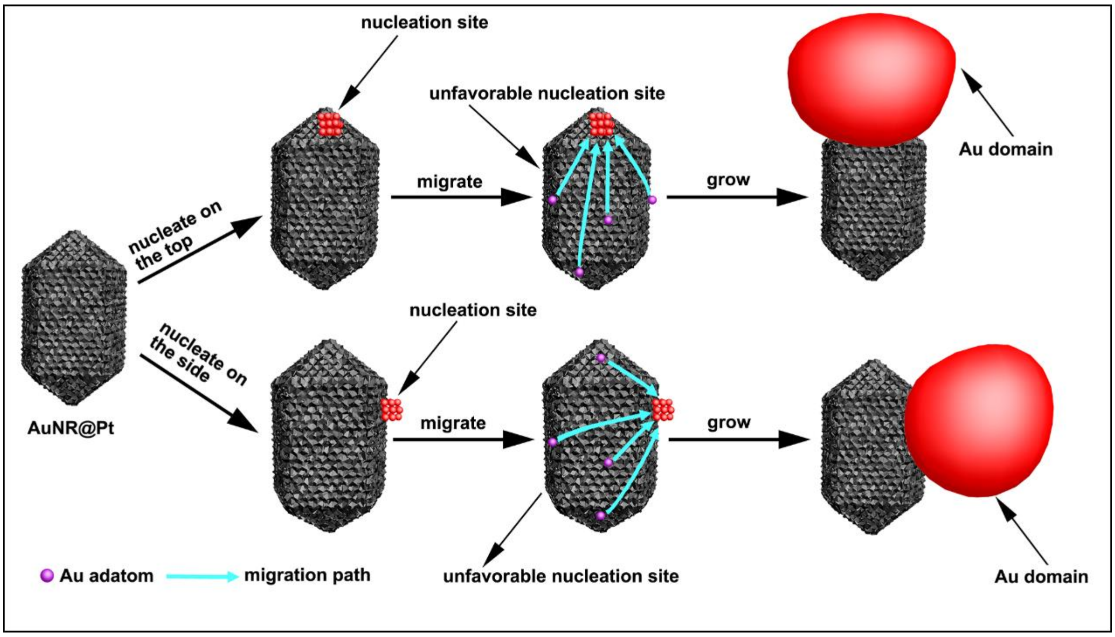


**Supplementary Fig. 48** Schematic illustration for the selective growth of Au on one side of AuNR@Pt nanocrystal seed.

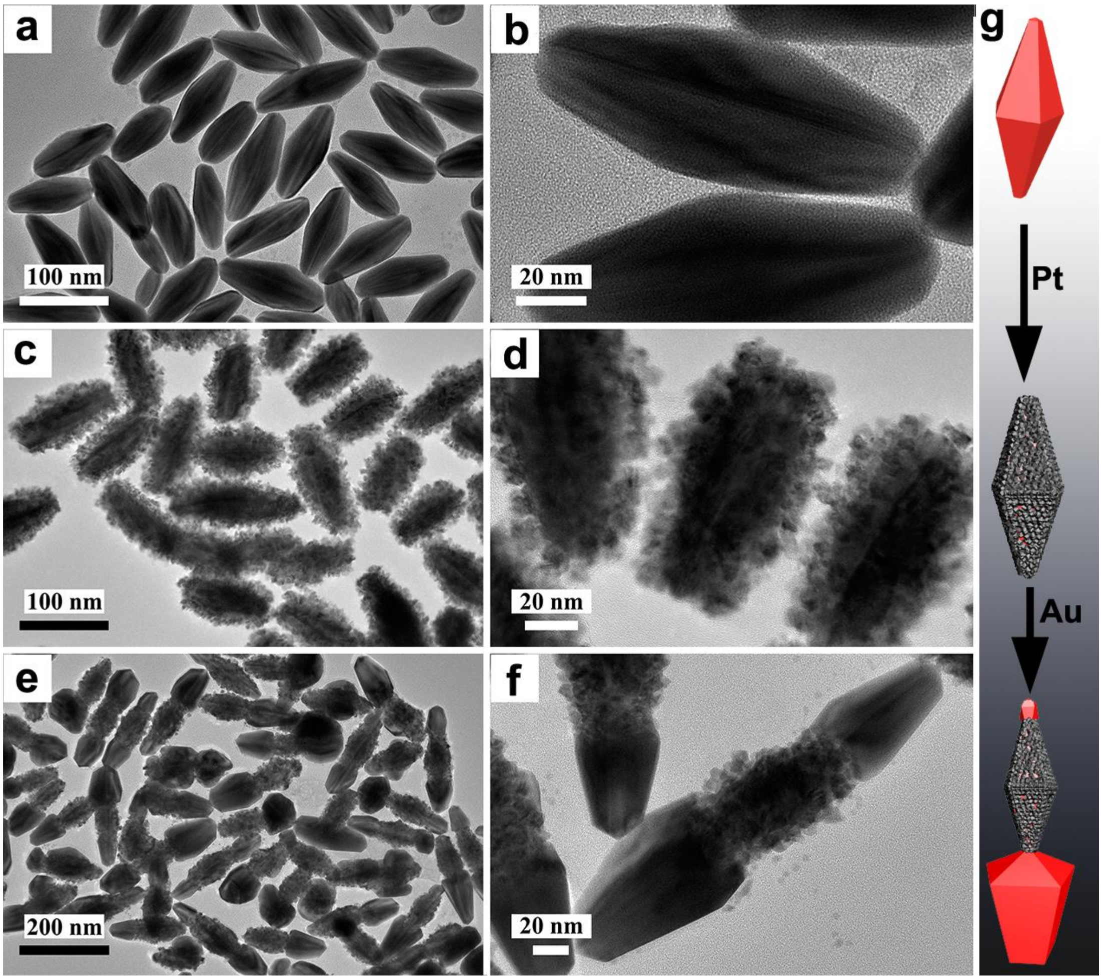


**Supplementary Fig. 49** TEM images of **a, b** AuNBs, **c**, **d** AuNB@Pt nanocrystals prepared through overgrowing Pt on the AuNBs (Pt/Au=1:4 mol/mol), and **e**, **f** multi-segmental nanocrystals prepared through overgrowing Au on the AuNB@Pt seeds ($HAuCl_4$/Au=4:1 mol/mol). **g** Schematic illustration of growth.

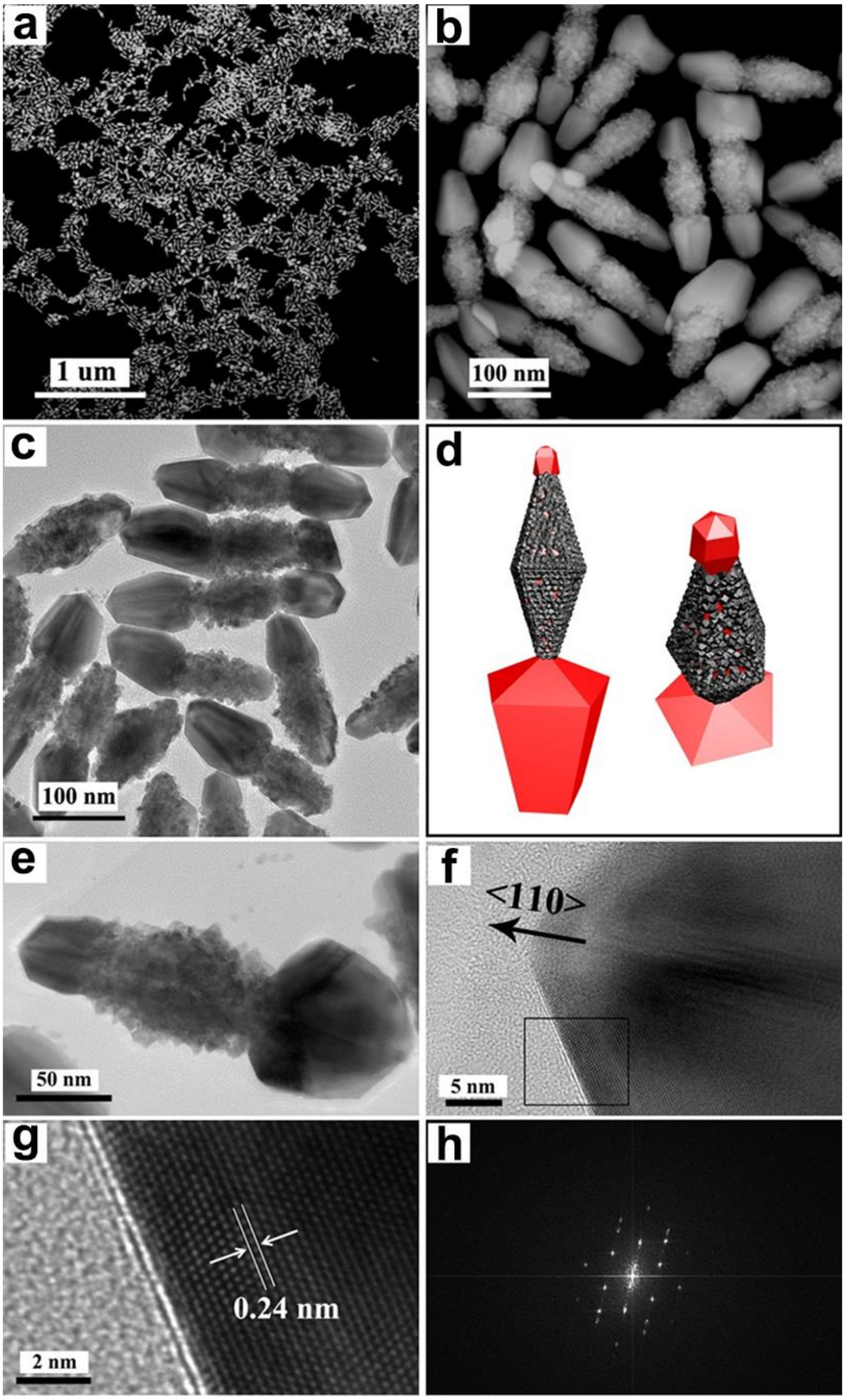


**Supplementary Fig. 50** **a**, **b** HAADF images, **c**, **e**, **f**, **g** TEM images, and **d** schematic model of nanocrystals shown in Supplementary Fig. 49c**.** **g** Detailed structure of area marked by the box in **f**. **h** FFT pattern obtained from the TEM image in **f**. The HAADF images in **a** and **b** demonstrate the high yield. 0.24 nm in **g** is assigned to the interplanar spacing of Au(111).

**Supplementary Note 4. Characterizations of AuTNO-based nanocrystals**

This note mainly presents the structure characterization of AuTNO nanocrystals, AuTNO@Pt nanocrystals, and their derivative products.

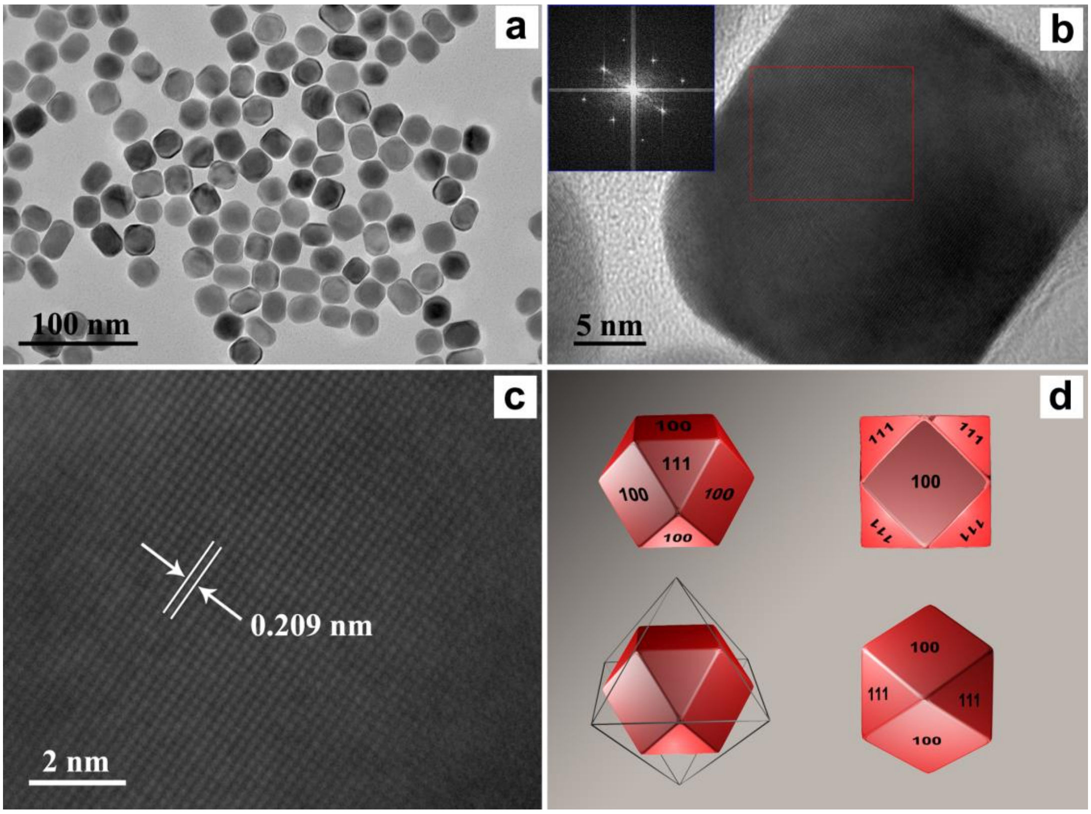


**Supplementary Fig. 51** **a** TEM image, **b**, **c** HRTEM images, and **d** schematic illustration of truncated Au octahedron nanocrystals. **c** Detailed structure of area marked by the red box in **b**. Clearly, almost all products are single crystalline.

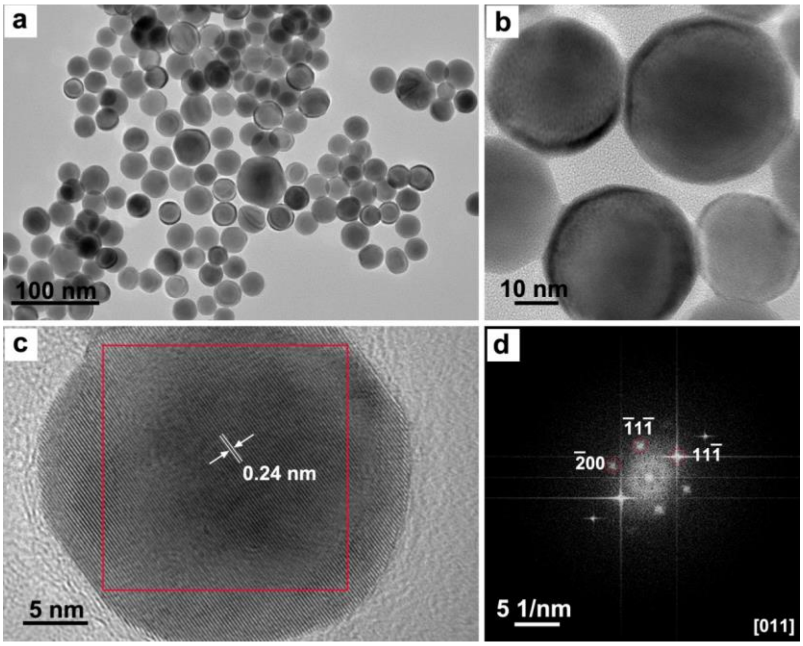


**Supplementary Fig. 52** **a** TEM image and **b**, **c** HRTEM images of products prepared through overgrowing Au on nanocrystals shown in Supplementary Fig. 51a-c. **d** Corresponding FFT pattern of the HRTEM result marked by the red box in **c**. The TEM image in **a** shows that most of products are spherical.

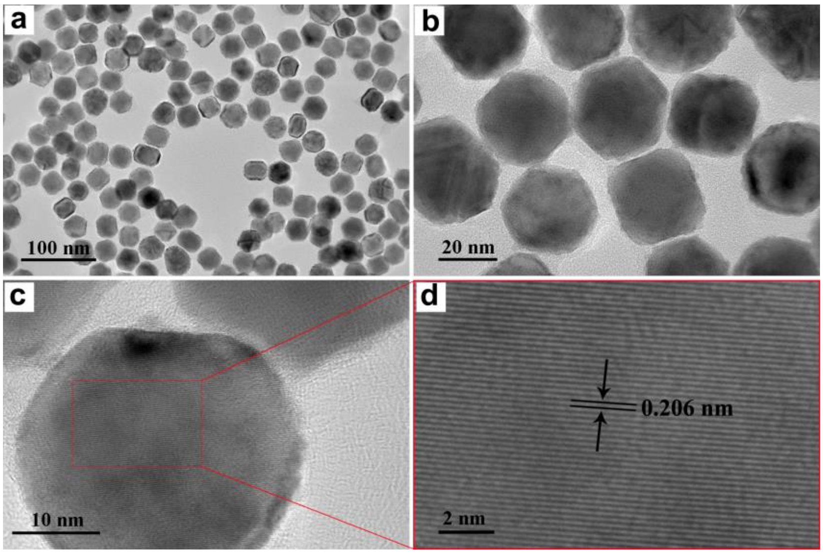


**Supplementary Fig. 53** **a** TEM image and **b**, **c**, **d** HRTEM images of products prepared through overgrowing Pt on nanocrystals shown in Supplementary Fig. 51a-c (The molar ratio of Pt and Au is 0.05). **d** Detailed structure of area marked by the red box in **c**. The HRTEM image in **d** shows that the lattice spacing of Au(200) has a little decrease due to Pt deposition.

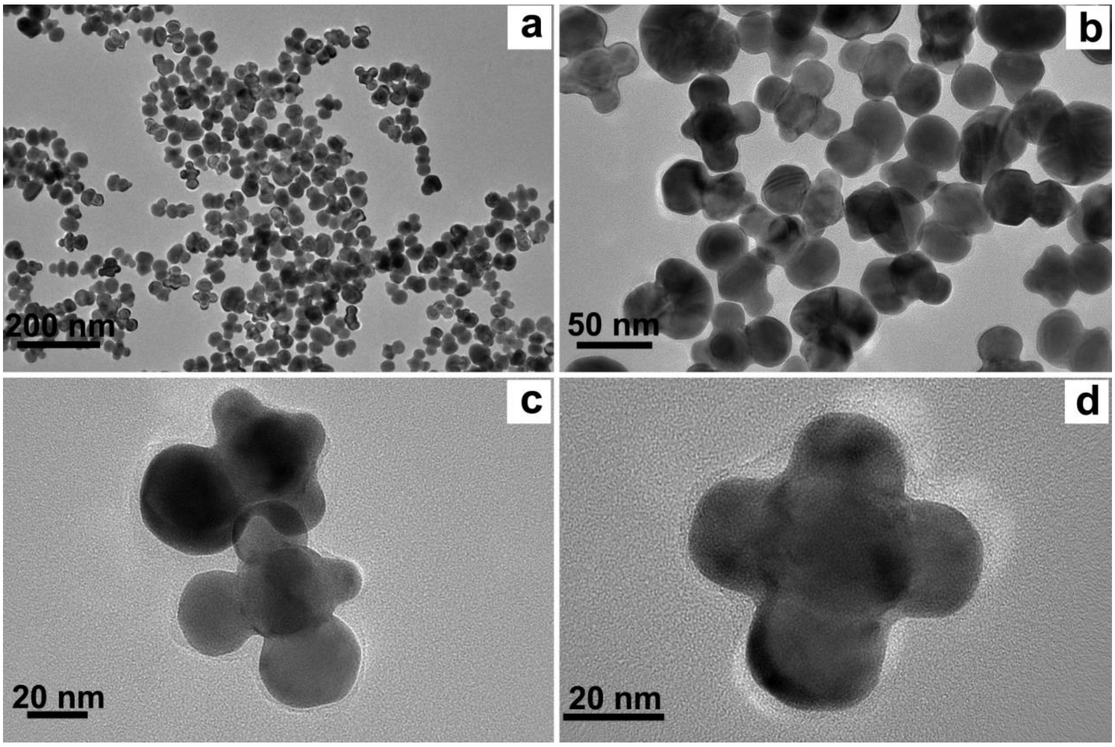


**Supplementary Fig. 54 a**, **b** TEM images and **c, d** HRTEM images of products prepared through overgrowing Au on nanocrystals shown in Supplementary Fig. 53. The molar ratio of $HAuCl_4$ and Au is 2 in the preparation. These TEM observations imply that products have multi-pod structure.

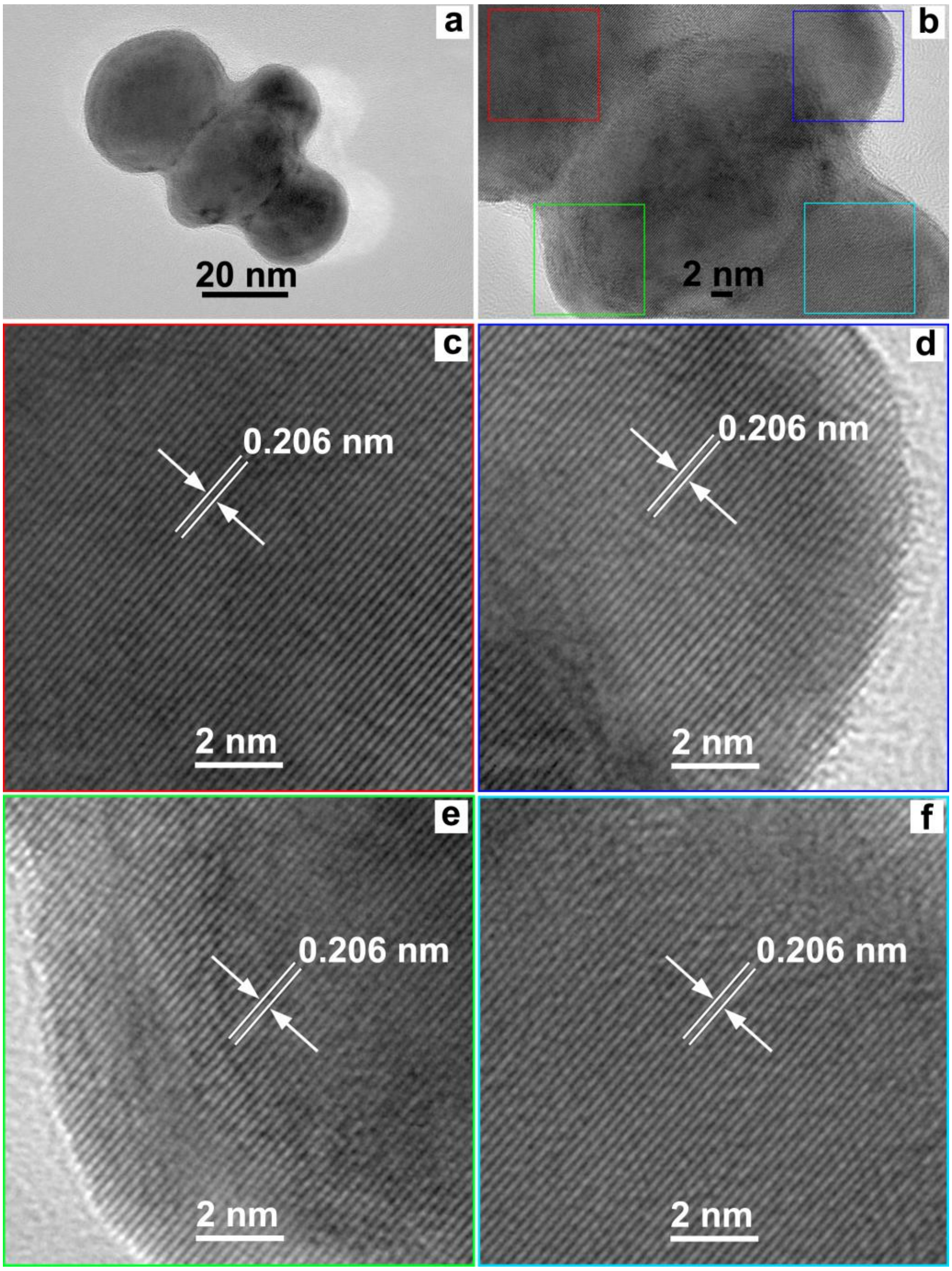


**Supplementary Fig. 55** **a**, **b**, **c**, **d**, **e**, **f** HRTEM images of one individual nanocrystal shown in Supplementary Fig. 54. **c**, **d**, **e**, and **f** are the details of areas marked by the red, blue, green, and cyan boxes in **b**, respectively. These HRTEM observations imply that the growth mainly occurred on {100} facets.

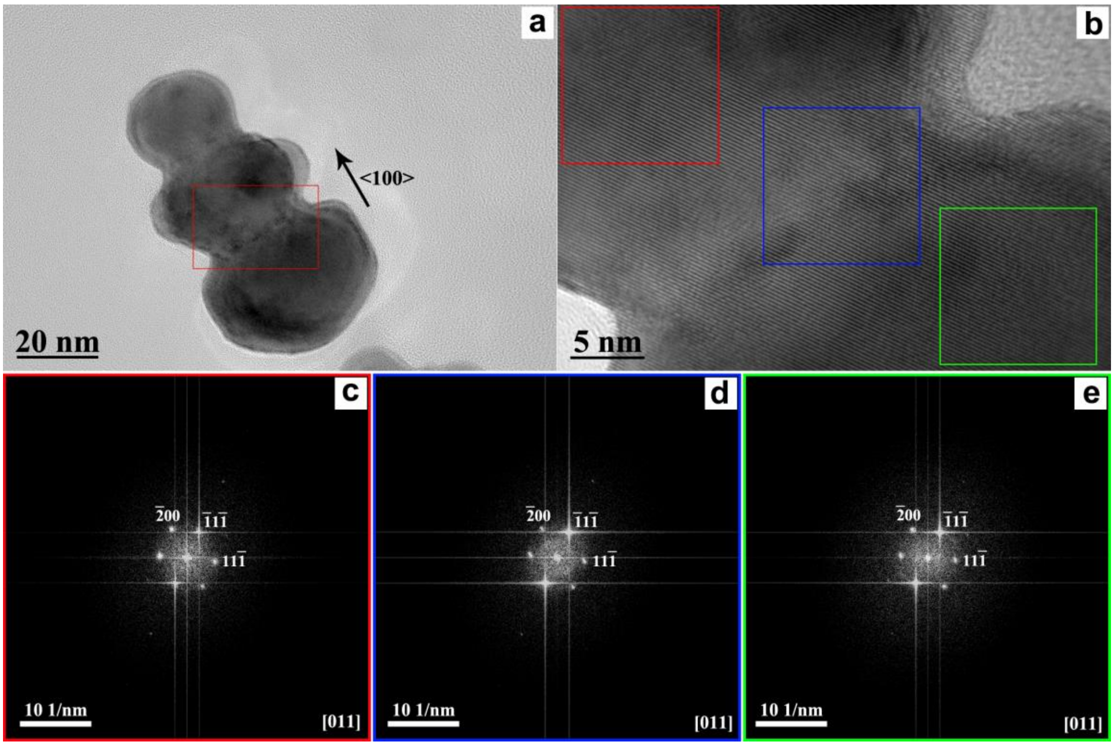


**Supplementary Fig. 56 a**, **b** HRTEM images of one nanocrystal shown in Supplementary Fig. 54. **c** FFT pattern obtained on the position marked by the red box in **b**, **d** FFT pattern obtained on the position marked by the blue box in **b**, and **e** FFT pattern obtained on the position marked by the green box in **b**. These results confirm that the growth happened along <100>.

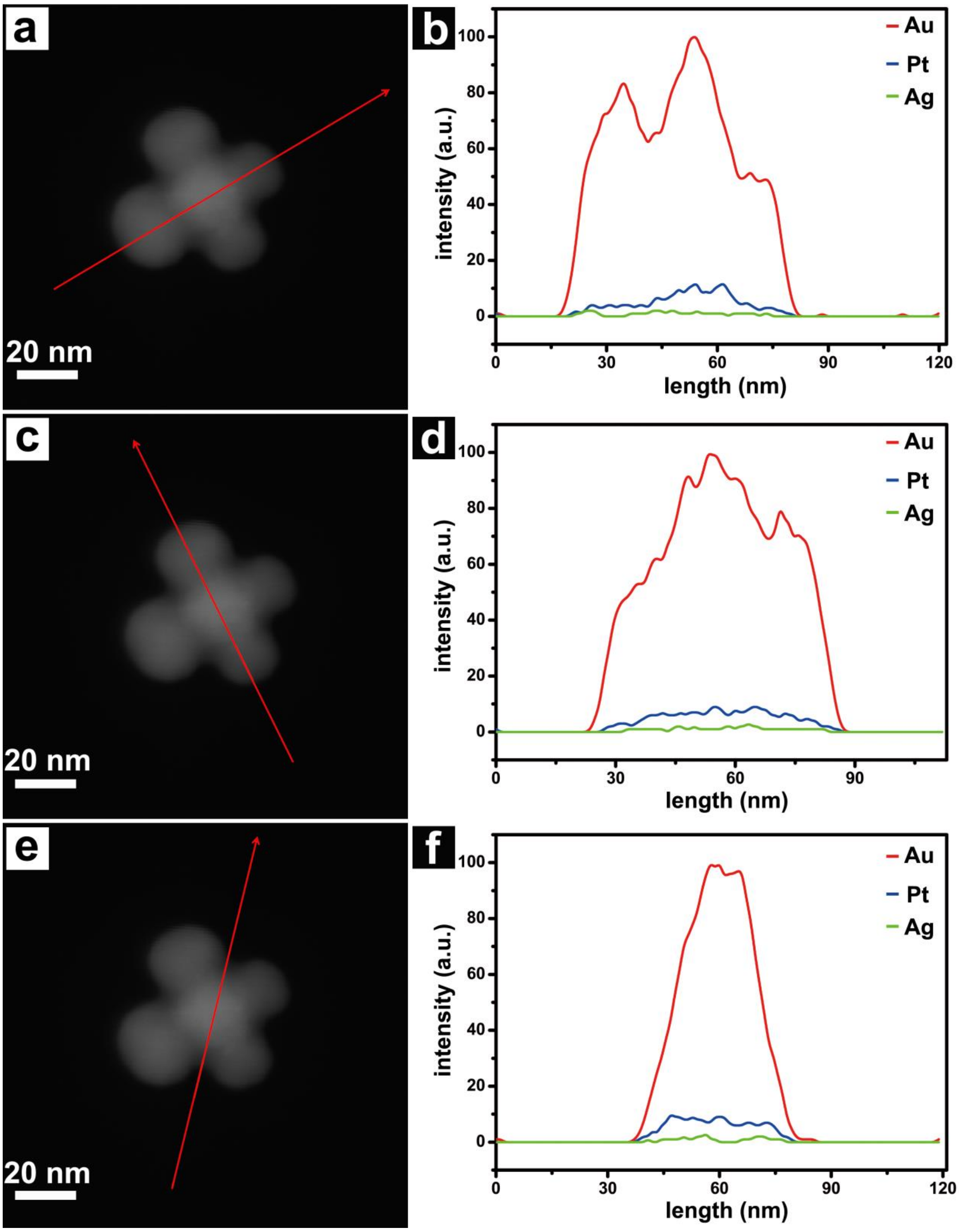


**Supplementary Fig. 57** **a** HAADF image of an individual nanocrystal from products shown in Supplementary Fig. 54 and **b** composition line profiles along the scanning direction marked by the red arrow in **a**. **c** HAADF image of the same nanocrystal and **d** composition line profiles along the scanning direction marked by the red arrow in **c**. **e** HAADF image of the same nanocrystal and **f** composition line profiles along the scanning direction marked by the red arrow in **e**.

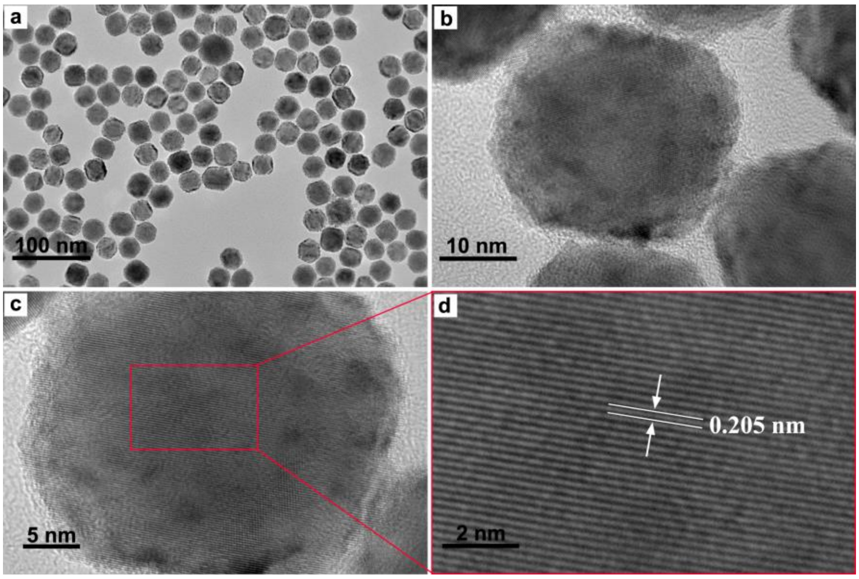


**Supplementary Fig. 58 a** TEM image and **b**, **c**, **d** HRTEM images of products prepared through overgrowing Pt on nanocrystals shown in Supplementary Fig. 51a-c. The molar ratio of Pt and Au is 0.15. **d** Detailed structure of area marked by the red box in **c**.

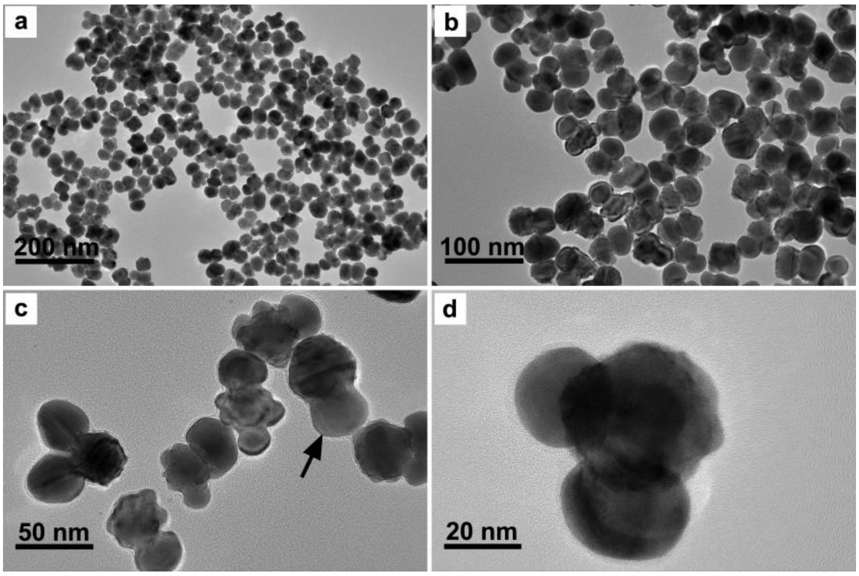


**Supplementary Fig. 59 a**, **b** TEM images and **c**, **d** HRTEM images of products prepared through overgrowing Au on nanocrystals shown in Supplementary Fig. 58. The molar ratio of $HAuCl_4$ and Au is 2 in the preparation. These TEM observations imply that products still have multi-lump structure, but the number of pod clearly decreased, demonstrating the growth symmetry became low. Even one-lump nanocrystal labeled by the arrow in **c** was observed.

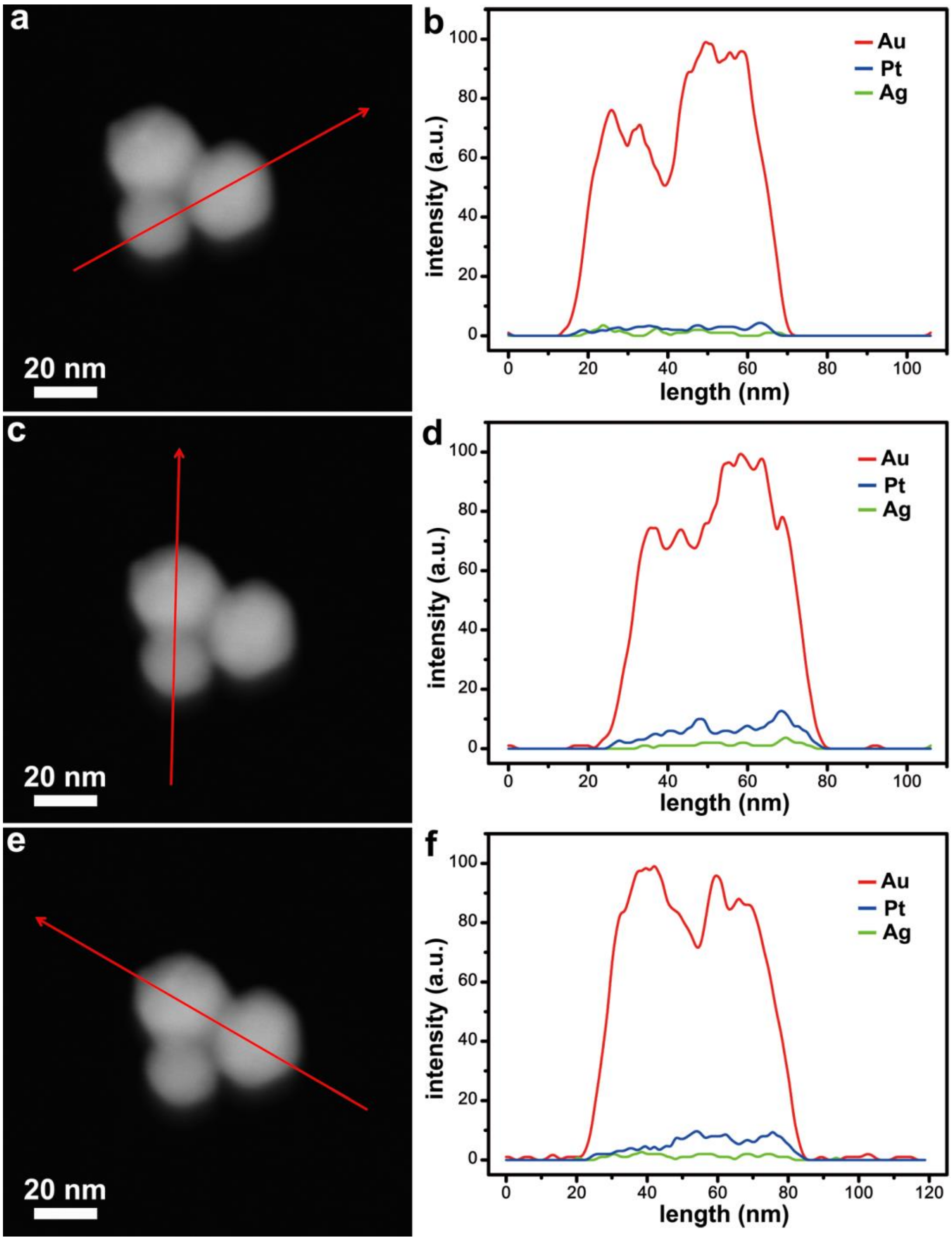


**Supplementary Fig. 60 a** HAADF image of an individual nanocrystal from products shown in Supplementary Fig. 59 and **b** composition line profiles along the scanning direction marked by the red arrow in **a**. **c** HAADF image of the same nanocrystal and **d** composition line profiles along the scanning direction marked by the red arrow in **c**. **e** HAADF image of the same nanocrystal and **f** composition line profiles along the scanning direction marked by the red arrow in **e**.

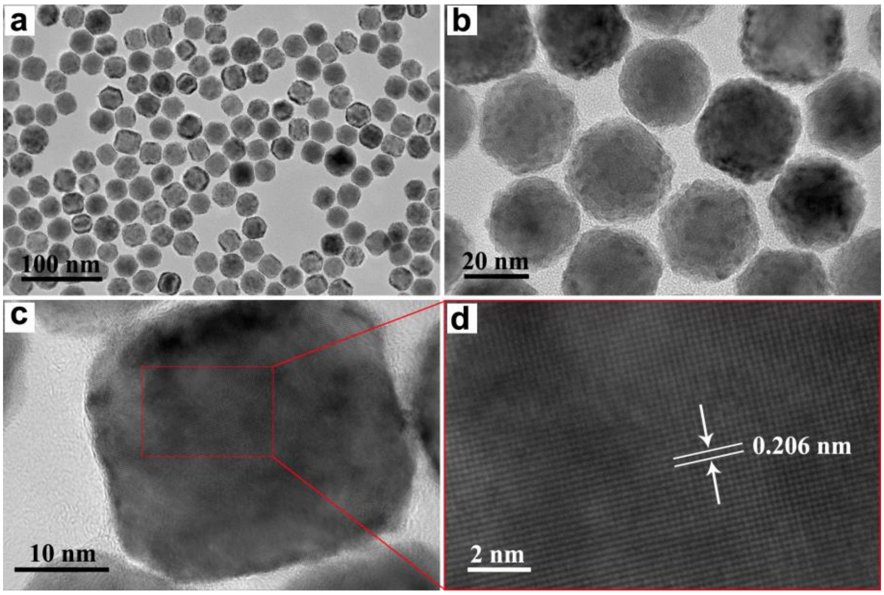


**Supplementary Fig. 61** **a**, **b** TEM images and **c**, **d** HRTEM images of products prepared through overgrowing Pt on nanocrystals shown in Supplementary Fig. 51a-c. The molar ratio of Pt and Au is 0.3. **d** Detailed structure of area marked by the red box in **c**.

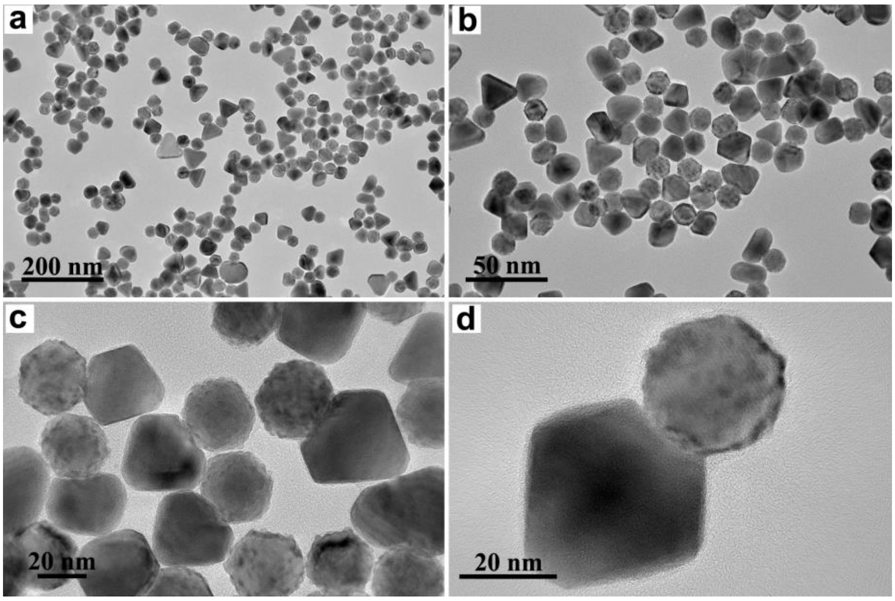


**Supplementary Fig. 62** **a**, **b** TEM images and **c**, **d** HRTEM images of products prepared through overgrowing Au on nanocrystals shown in Supplementary Fig. 61. The molar ratio of $HAuCl_4$ and Au is 2 in the preparation. These TEM observations imply that almost all products are dimer, demonstrating the growth symmetry further decreased with Pt content on seed surface.

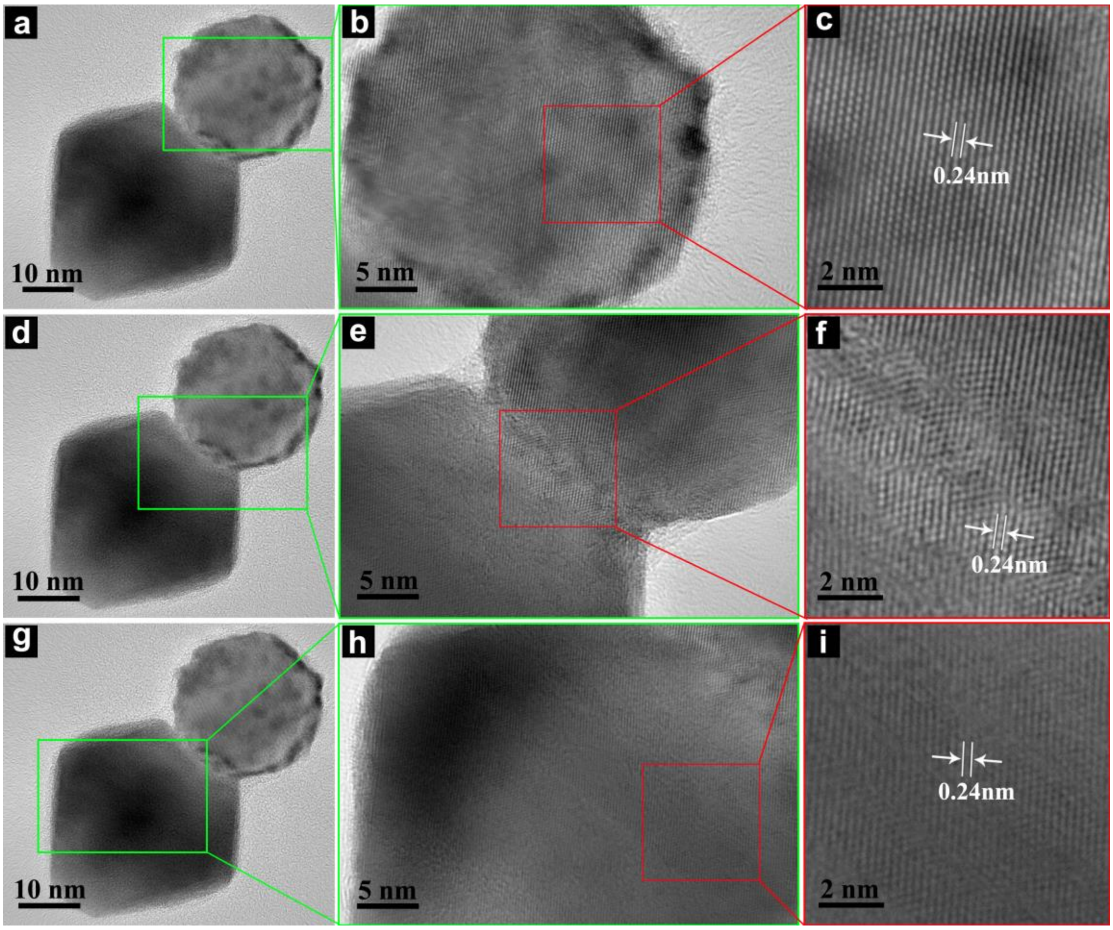


**Supplementary Fig. 63** HRTEM images of different positions in the nanocrystal shown in Supplementary Fig. 62d: **a**, **b**, **c** AuTNO@Pt; **d**, **e**, **f** the interface between AuTNO@Pt and newly-formed Au domain; **g**, **h**, **i** newly-formed Au domain. **b**, **e**, and **h** are the details of areas marked by the green boxes in **a**, **d**, and **g**, respectively. **c**, **f**, and **i** are the details of areas marked by the red boxes in **b**, **e**, and **h**, respectively.

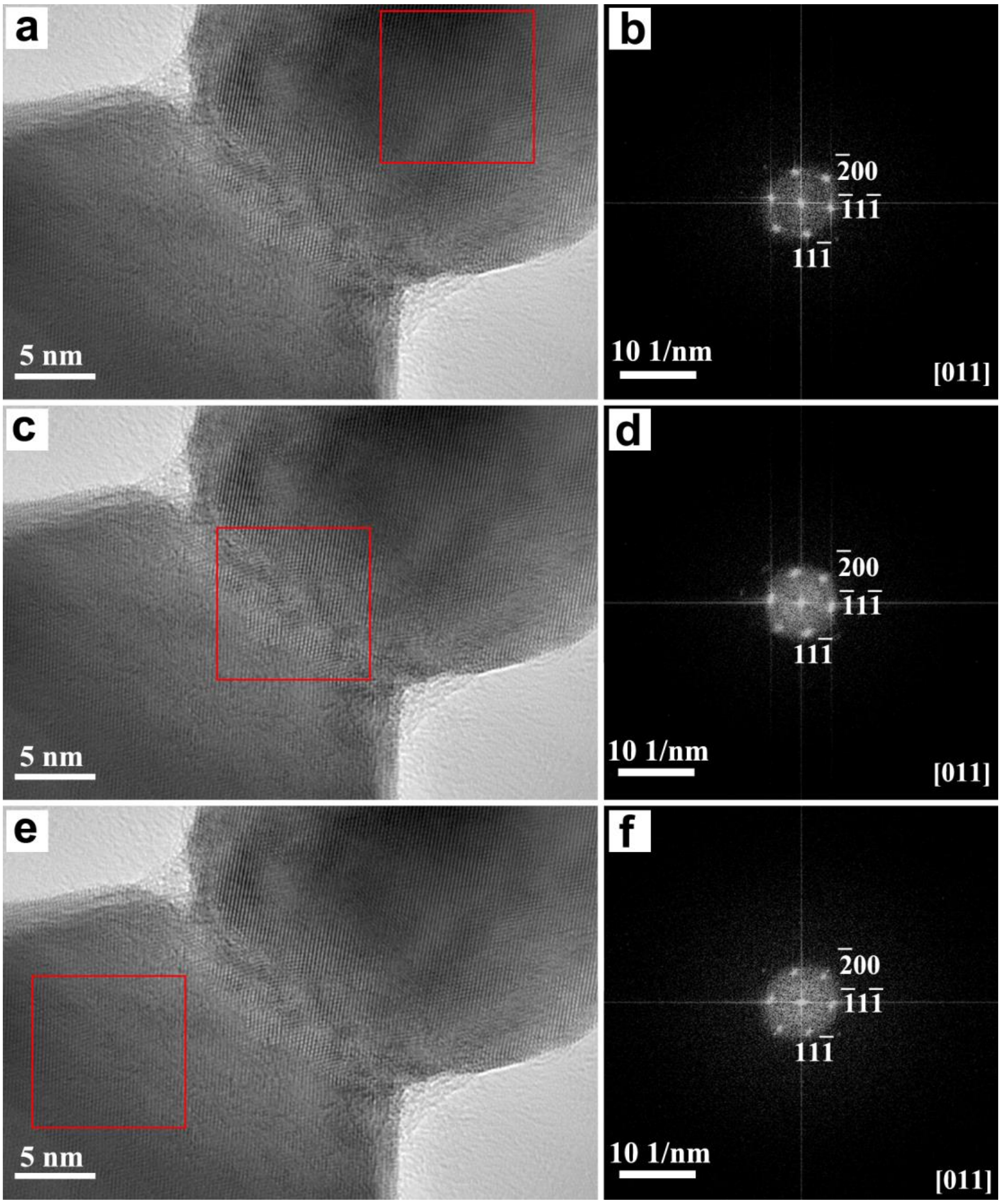


**Supplementary Fig. 64** HRTEM images and corresponding FFT patterns of different positions in the nanocrystal shown in Supplementary Fig. 62d: **a**, **b** AuTNO@Pt; **c**, **d** the interface between AuTNO@Pt and newly-formed Au domain; **e**, **f** newly-formed Au domain. These results confirm that the growth orientation had no change and still occurred along <100>.

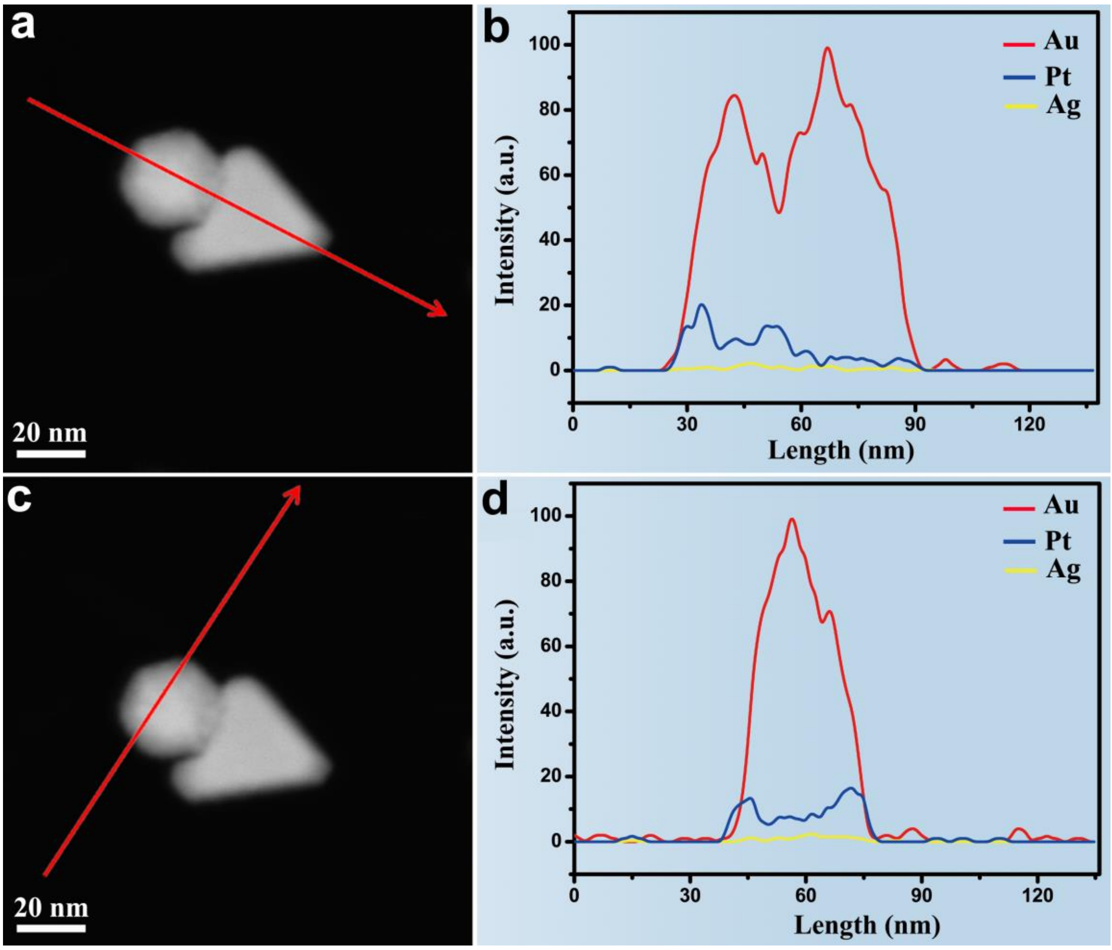


**Supplementary Fig. 65 a** HAADF image of an individual nanocrystal from products shown in Supplementary Fig. 62 and **b** composition line profiles along the scanning direction marked by the red arrow in **a**. **c** HAADF image of the same nanocrystal and **d** composition line profiles along the scanning direction marked by the red arrow in **c**.

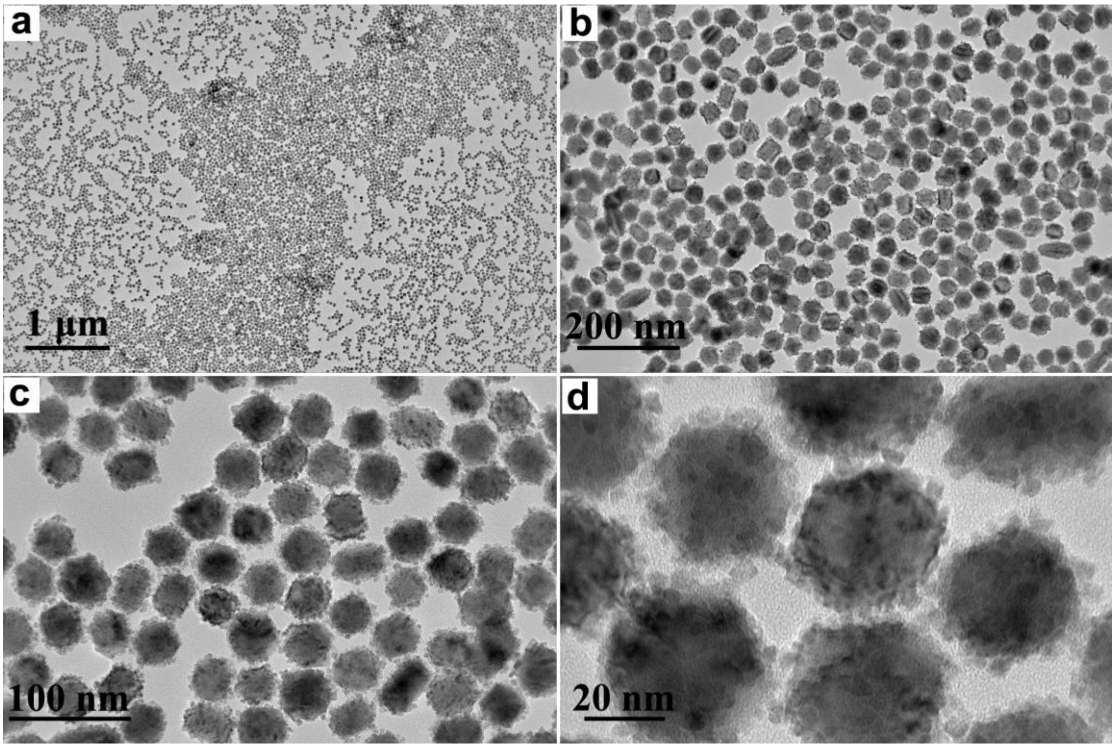


**Supplementary Fig. 66 a**, **b**, **c** TEM images and **d** HRTEM image of products prepared through overgrowing Pt on nanocrystals shown in Supplementary Fig. 51a. The molar ratio of Pt and Au is 0.5.

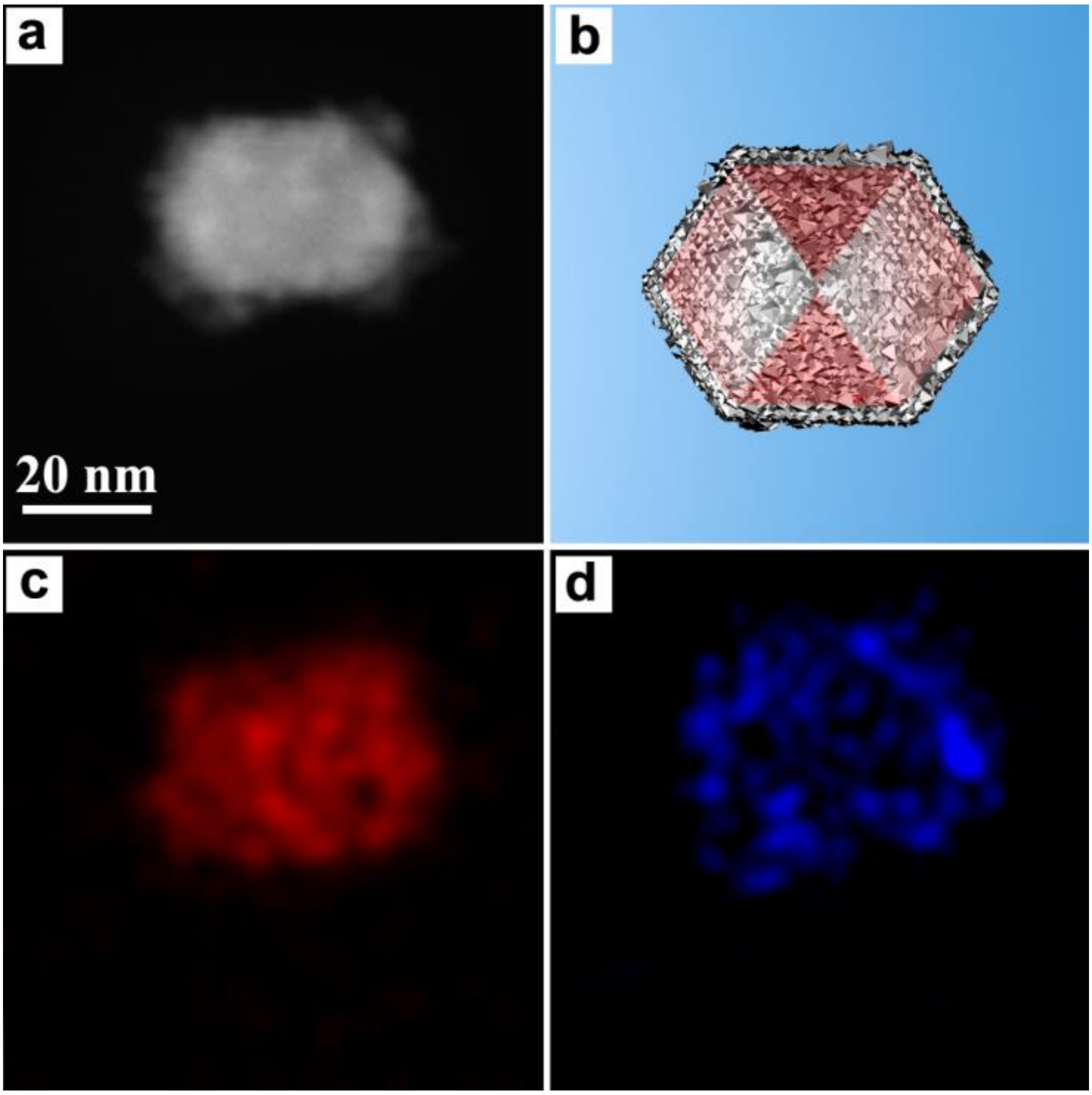


**Supplementary Fig. 67** **a** HAADF image, **b** schematic illustration, and **c**, **d** elemental maps of one individual nanocrystal shown in Supplementary Fig. 66.

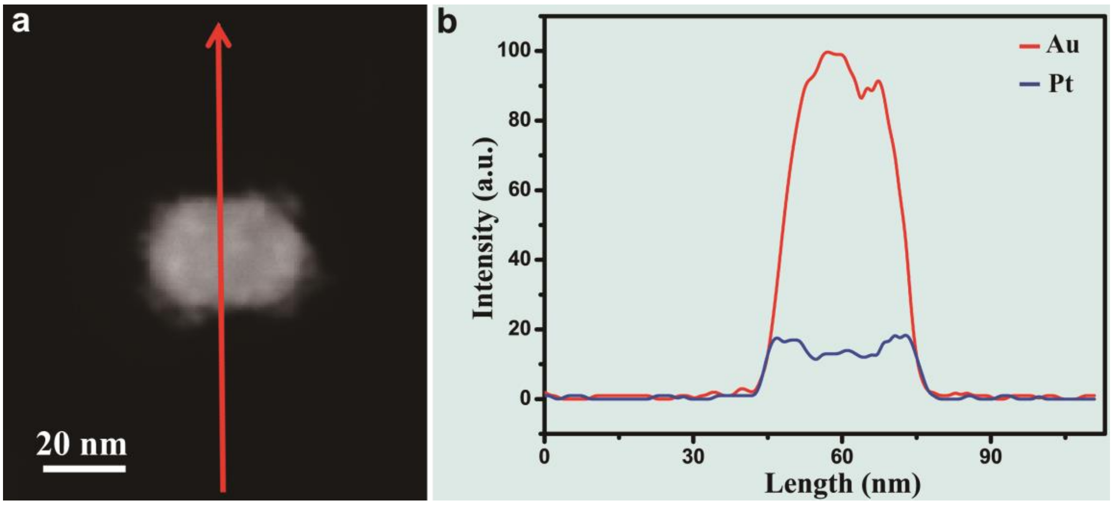


**Supplementary Fig. 68 a** HAADF image and **b** composition line profiles of the nanocrystal shown in Supplementary Fig. 67. The red arrow in **a** indicates the directions of composition line scanning.

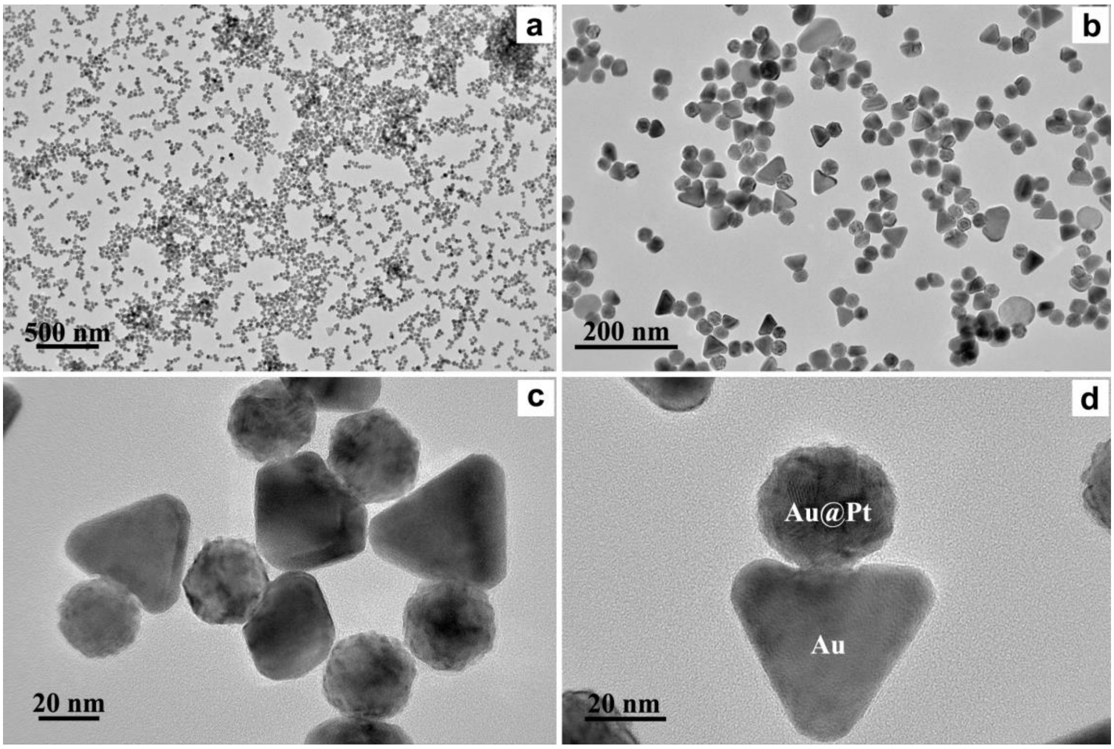


**Supplementary Fig. 69 a**, **b** TEM images and **c**, **d** HRTEM images of products prepared through overgrowing Au on nanocrystals shown in Supplementary Fig. 66. The molar ratio of $HAuCl_4$ and Au is 2 in the preparation.

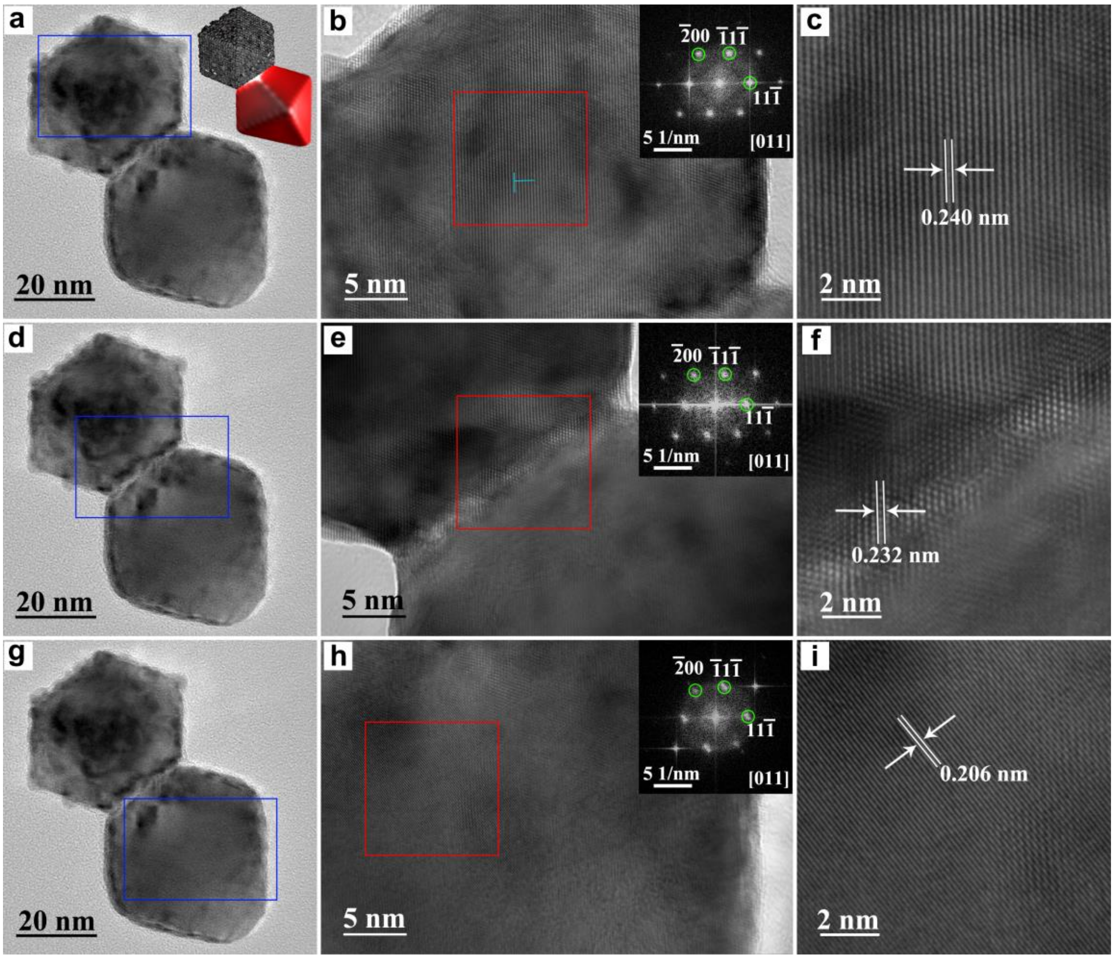


**Supplementary Fig. 70** HRTEM images of different positions in the nanocrystal shown in Supplementary Fig. 69: **a**, **b**, **c** AuTNO@Pt; **d**, **e**, **f** the interface between AuTNO@Pt and newly-formed Au domain; **g**, **h**, **i** newly-formed Au domain. **b**, **e**, and **h** are the details of areas marked by the blue boxes in **a**, **d**, and **g**, respectively. The insets in **b**, **e**, and **h** are the corresponding FFT patterns of HRTEM results marked by the red boxes in **b**, **e**, and **h**, respectively. **c**, **f**, and **i** are the details of areas marked by the red boxes in **b**, **e**, and **h**, respectively. These results demonstrates again that the growth of Au domain along <100>. The HRTEM images in **c** and **f** clearly also show that the lattice spacing near interface is smaller than that in other positions.

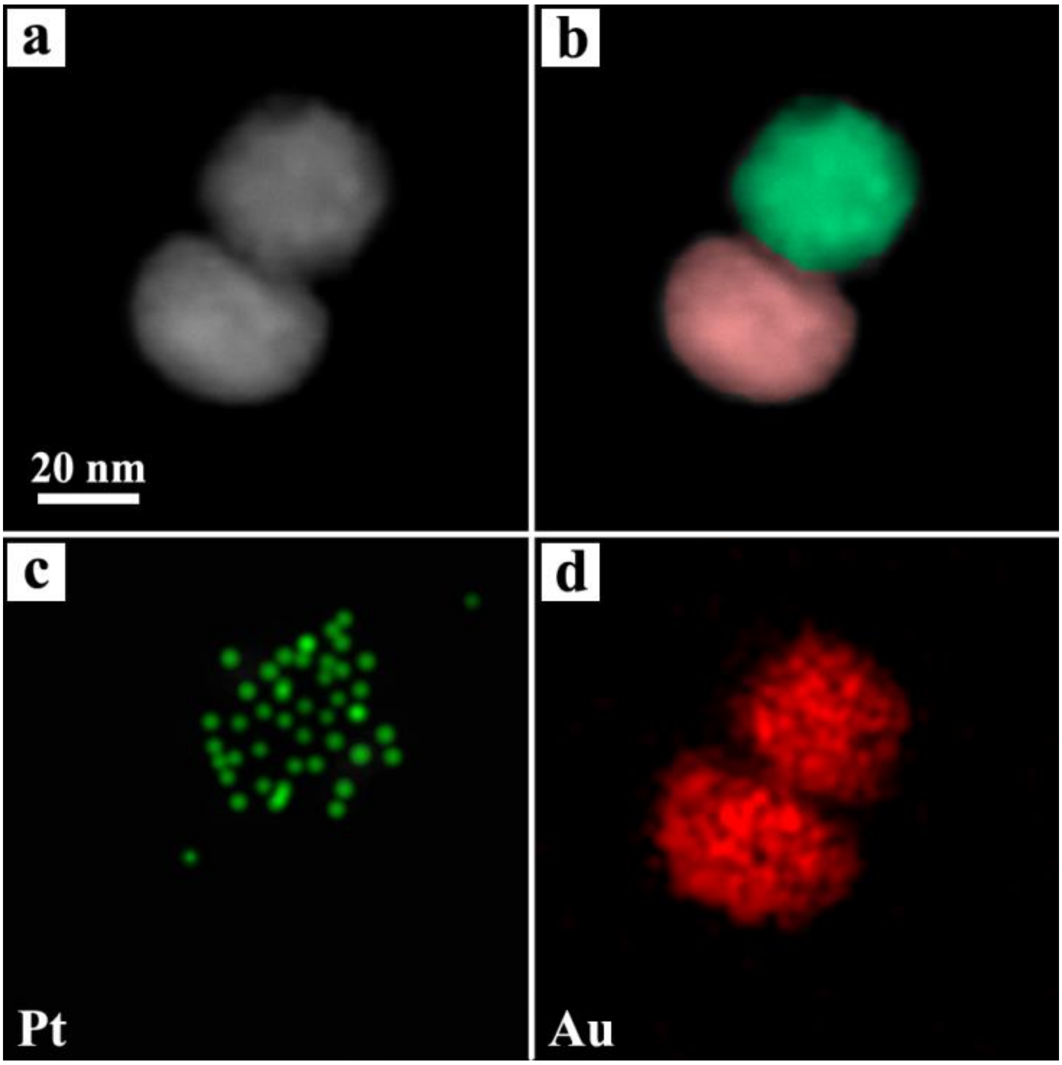


**Supplementary Fig. 71 a** HAADF image and **b**, **c**, **d** elemental maps of one individual nanocrystal shown in Supplementary Fig. 69.

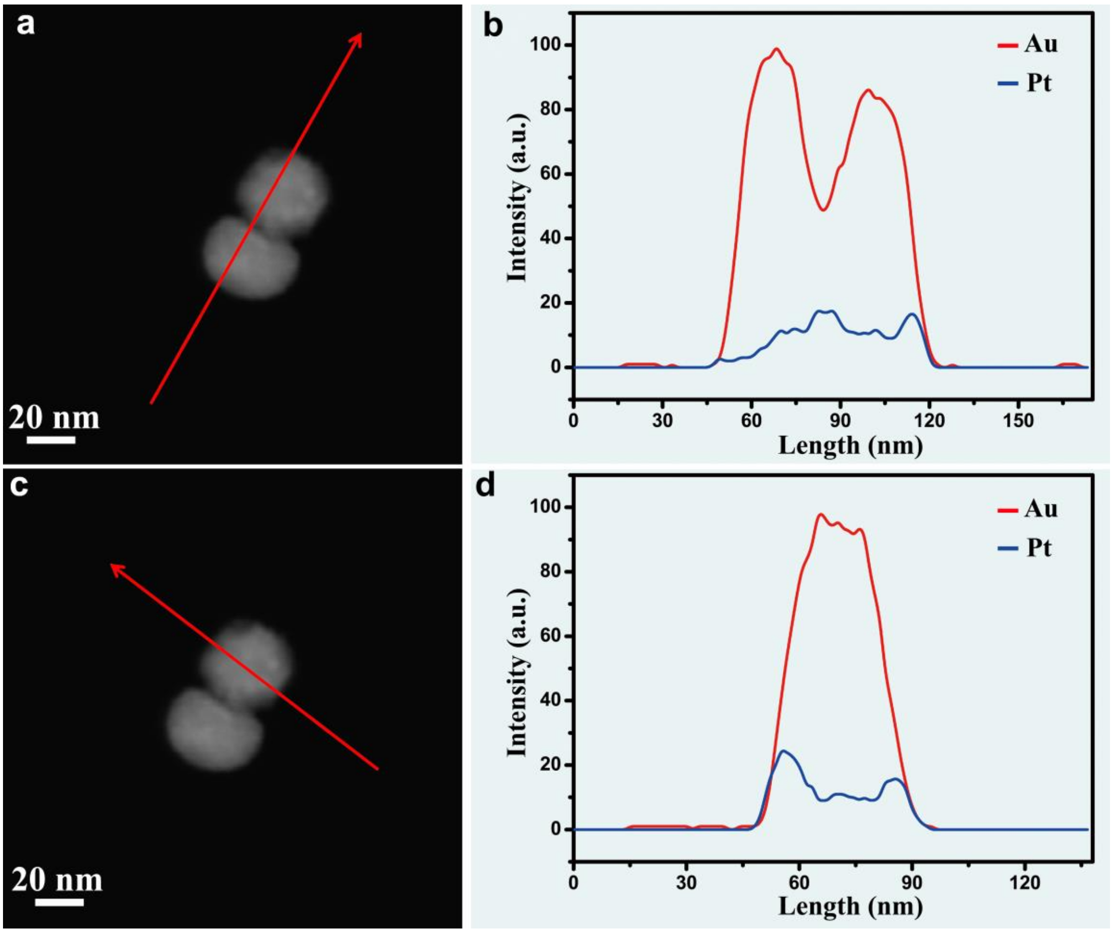


**Supplementary Fig. 72 a** HAADF image of an individual nanocrystal from products shown in Supplementary Fig. 71 and **b** composition line profiles along the scanning direction marked by the red arrow in **a**. **c** HAADF image of the same nanocrystal and **d** composition line profiles along the scanning direction marked by the red arrow in **c**.

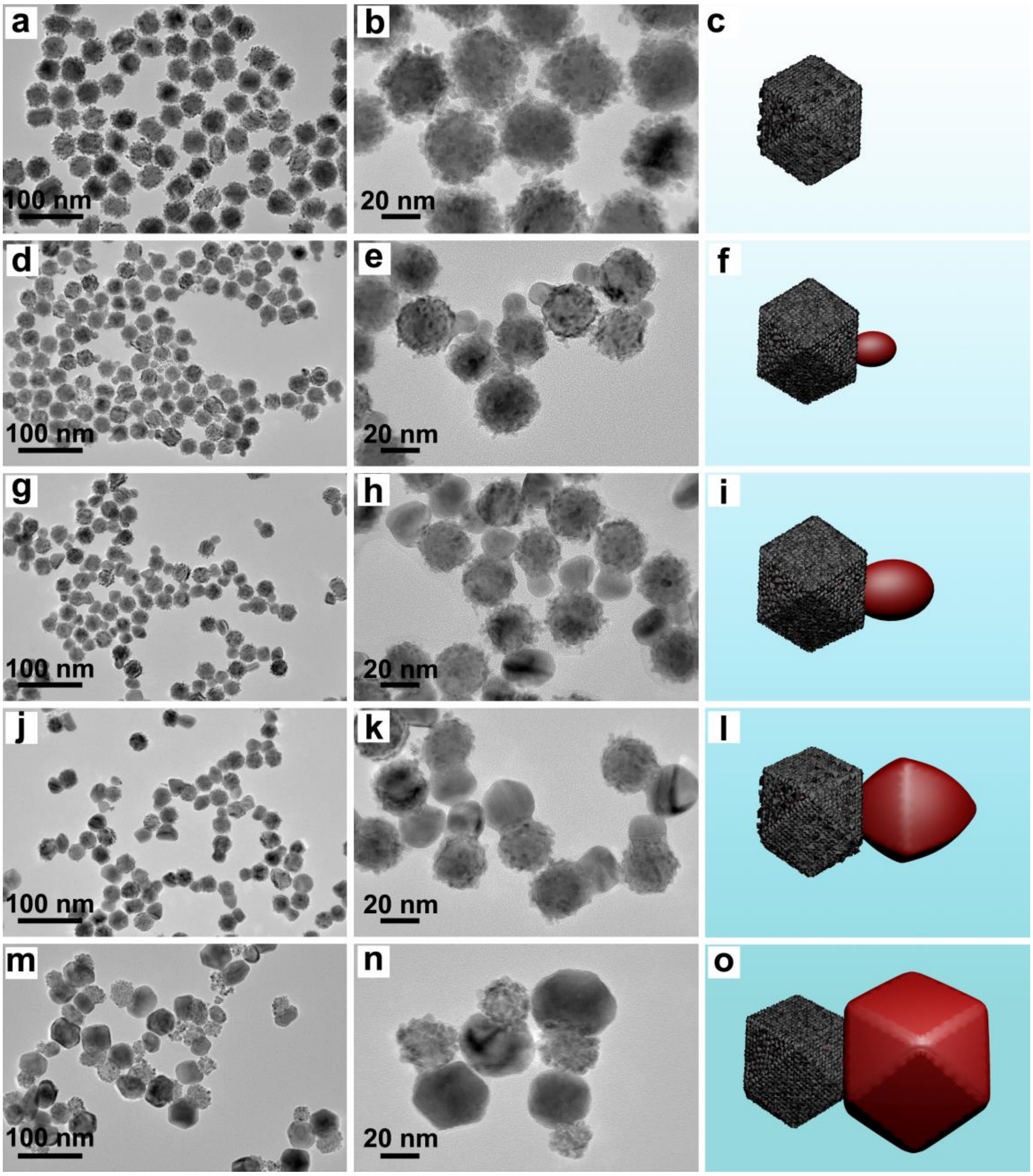


**Supplementary Fig. 73** TEM images and schematic illustrations of products prepared through tuning the molar ratio of $HAuCl_4$ and Au when using AuTNO@Pt nanocrystals as seeds (molar ratio of Pt and Au in the AuTNO@Pt seeds was 1): **a**, **b**, **c** 0; **d**, **e**, **f** 0.2; **g**, **h**, **i** 0.4; **j**, **k**, **l** 0.8; **m**, **n**, **o** 3.

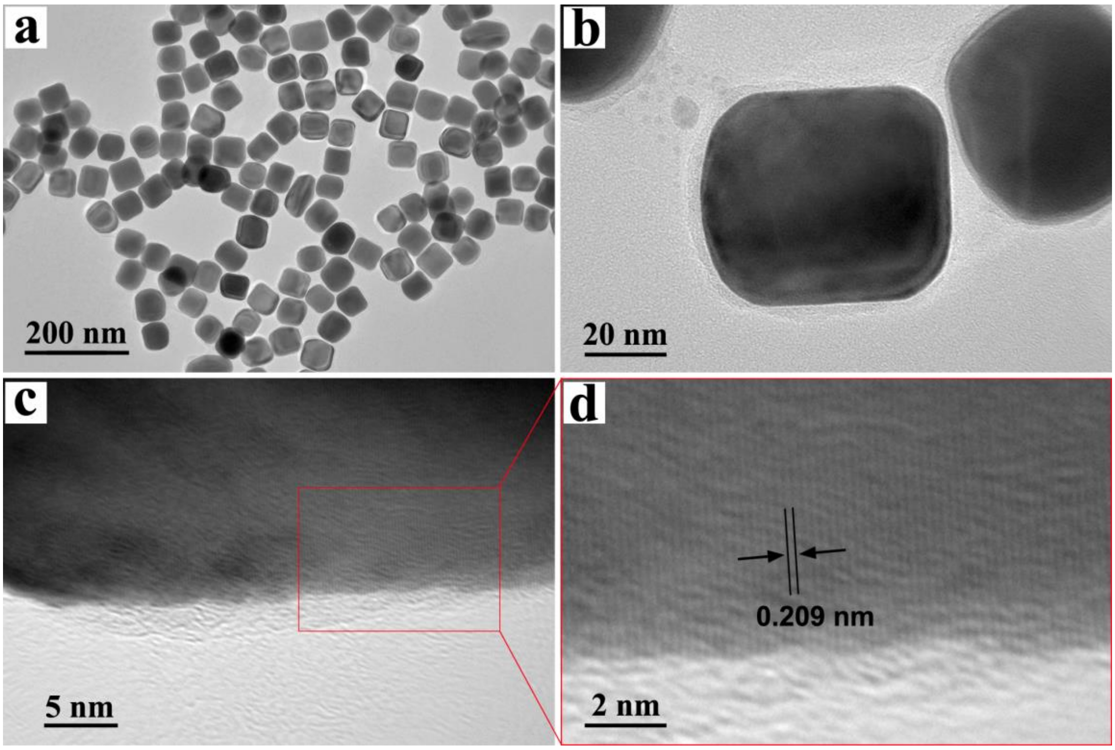


**Supplementary Fig. 74 a** TEM image and **b**, **c** HRTEM images of AuTNO nanocrystal. **d** Detailed structure of the area marked by the red box in **c**.

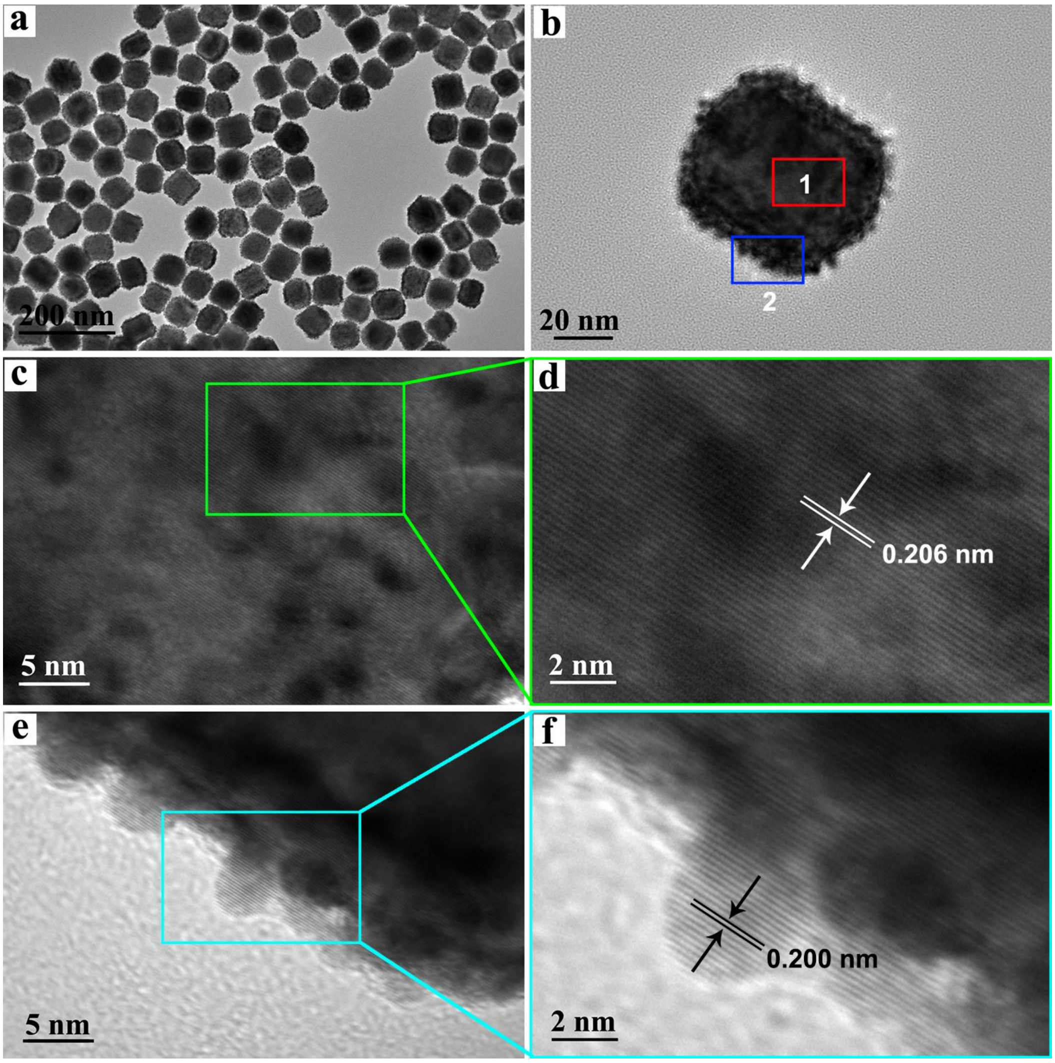


**Supplementary Fig. 75** **a**, **b** TEM images and **c**, **d**, **e**, **f** HRTEM images of AuTNO@Pt nanocrystals prepared through overgrowing Pt on AuTNO seeds shown in Supplementary Fig. 74. The HRTEM images in **c** and **e** are the detailed structures of region 1 and 2 in **b**, respectively. **d** Detailed structure of the area marked by the green box in **c**. **f** Detailed structure of the area marked by the cyan box in **e**.

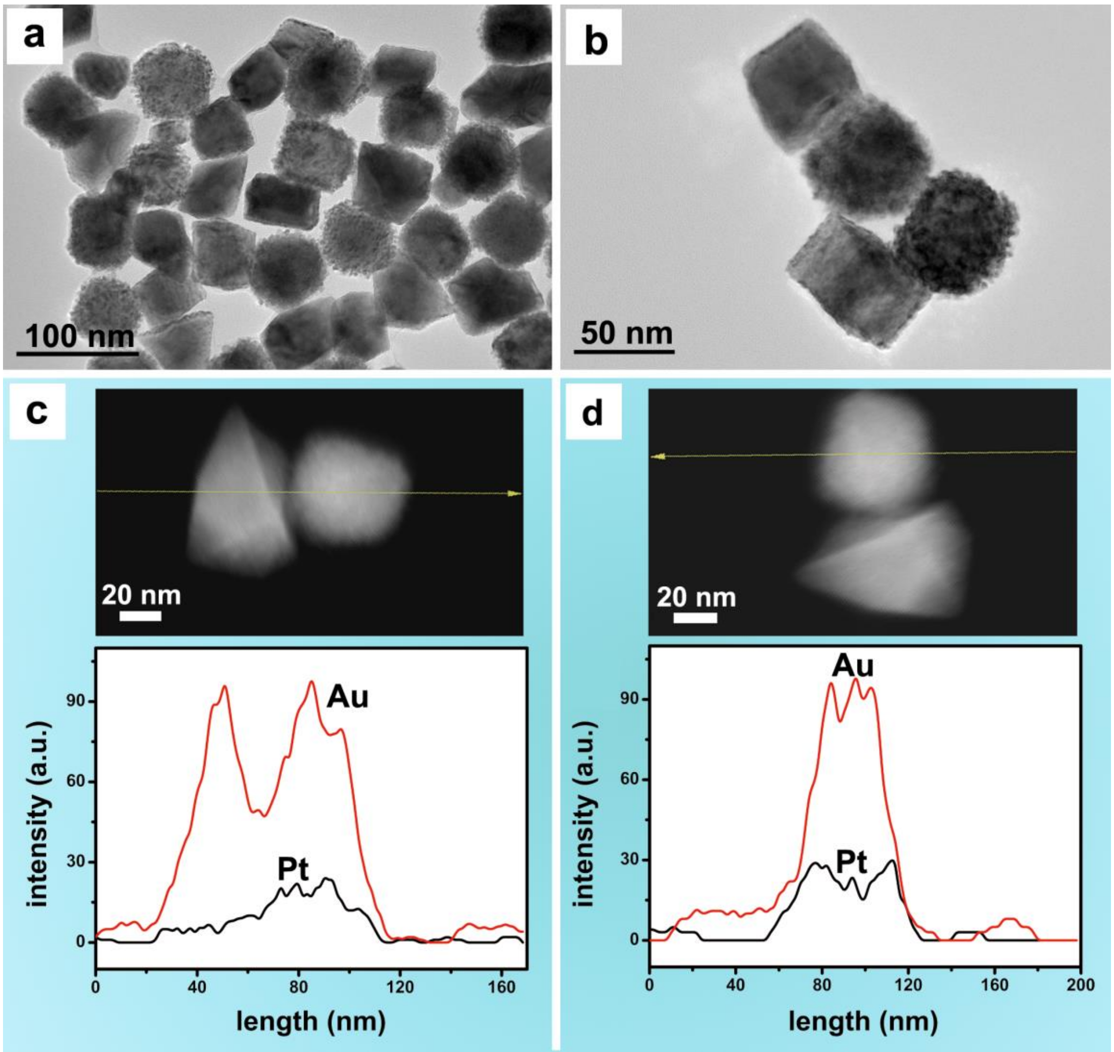


**Supplementary Fig. 76** **a** TEM image and **b** HRTEM image of nanocrystals prepared through overgrowing Au on AuTNO@Pt seeds shown in Supplementary Fig. 75. **c**, **d** HAADF images and **c**omposition line profiles of one nanocrystal along different directions indicated by the yellow arrows.

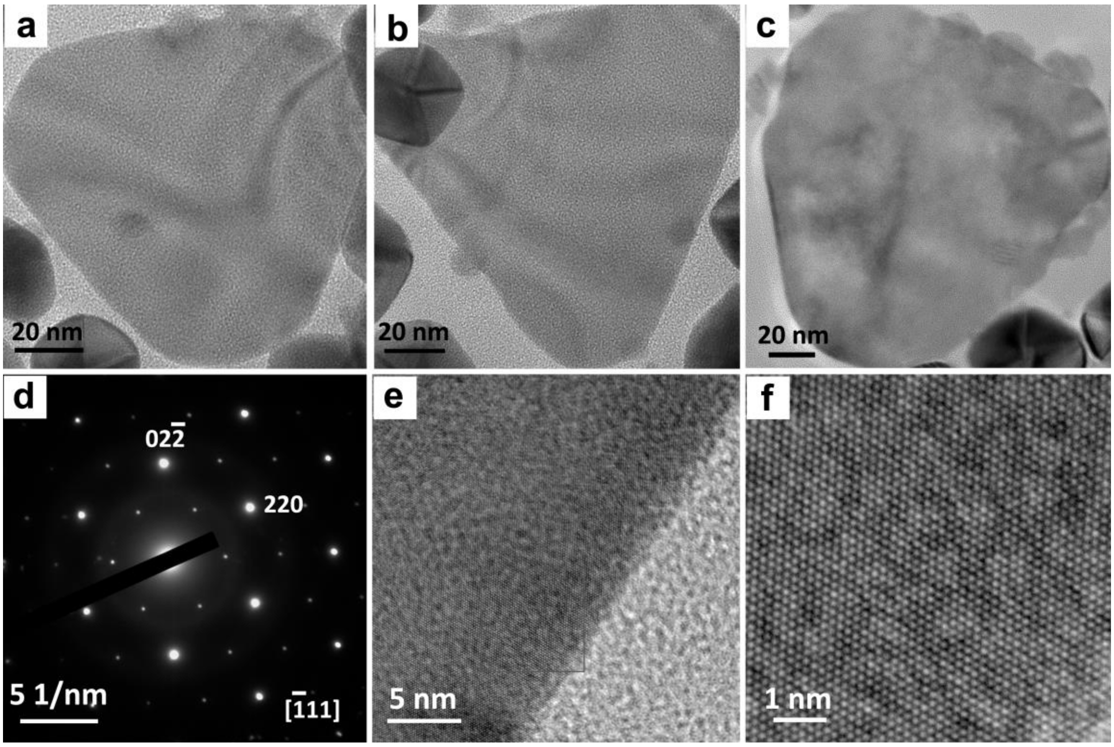


**Supplementary Fig. 77 a**, **b**, **c** TEM images, **d** selected area electron diffraction (SEAD) pattern, and **e**, **f** HRTEM images of an individual Au nanoplate (AuNP). The HRTEM image in **f** clearly shows the cubic closest packing.

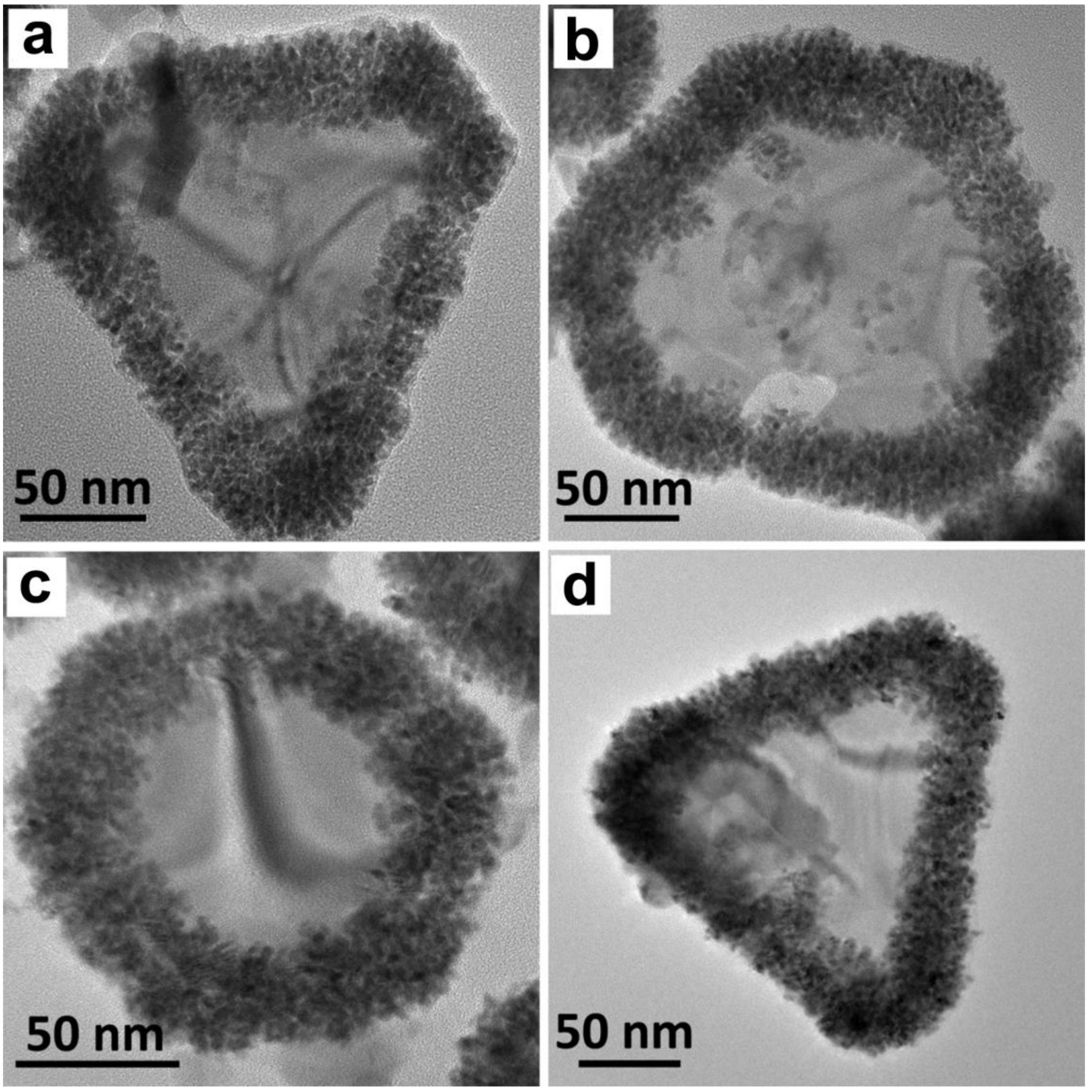


**Supplementary Fig. 78 a**, **b**, **c**, **d** HRTEM images of AuNP@PtNC nanocrystal (Pt/Au=2:1 mol/mol).

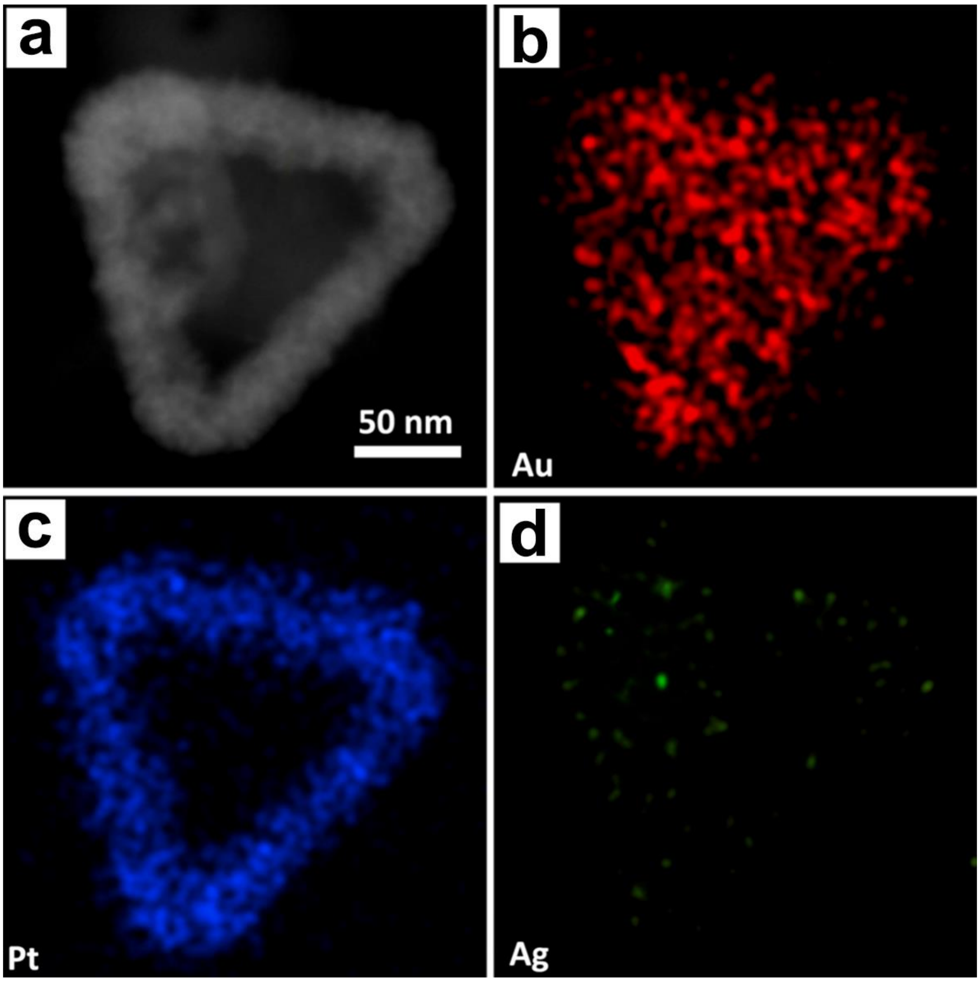


**Supplementary Fig. 79** **a** HAADF image and **b**, **c, d** EDS elemental maps of an individual AuNP@PtNC nanocrystal.

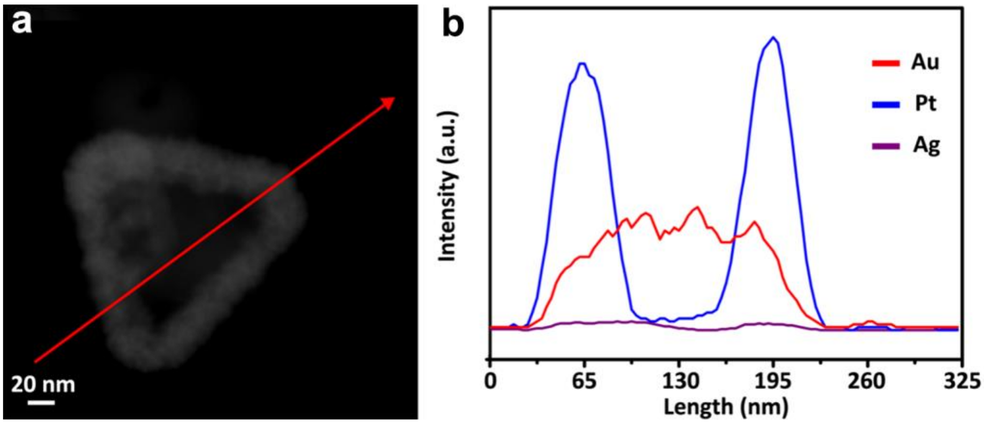


**Supplementary Fig. 80** **a** HAADF image and **b** composition line profiles of the AuNP@PtNC nanocrystal shown in Supplementary Fig. 79a. The red arrow in **a** indicates the direction of composition line scanning.

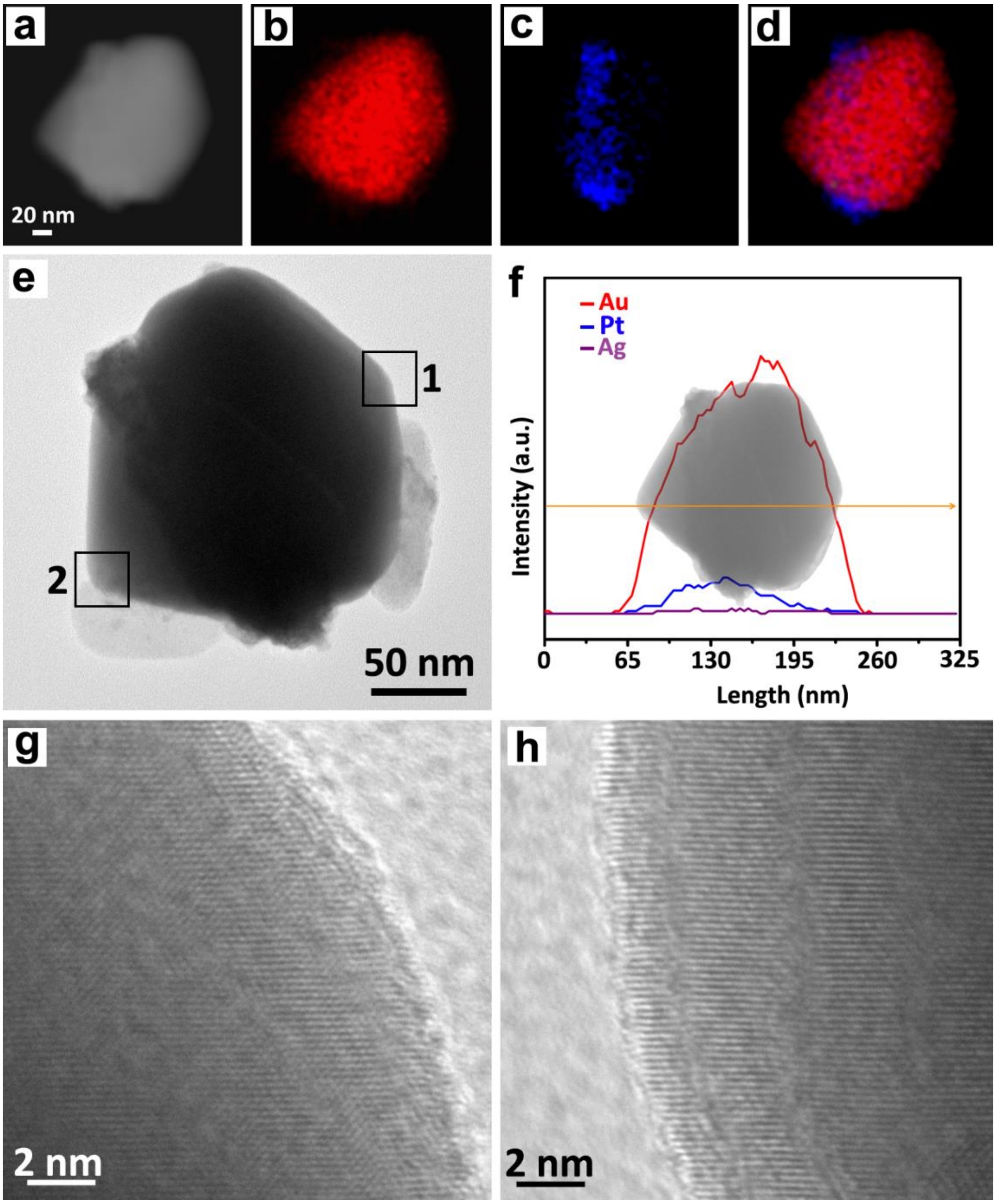


**Supplementary Fig. 81** **a**, **b**, **c**, **d** Elemental maps, **e** TEM image, **f** composition line profiles (The arrow indicates the scanning direction), and **g**, **h** HRTEM images of an individual nanocrystal prepared through overgrowing Au on an AuNP@PtNC seed. **g** and **h** are the details of areas marked by the black boxes 1 and 2 in **e**, respectively. These HRTEM images show that no twinned interfaces are present in two tips.

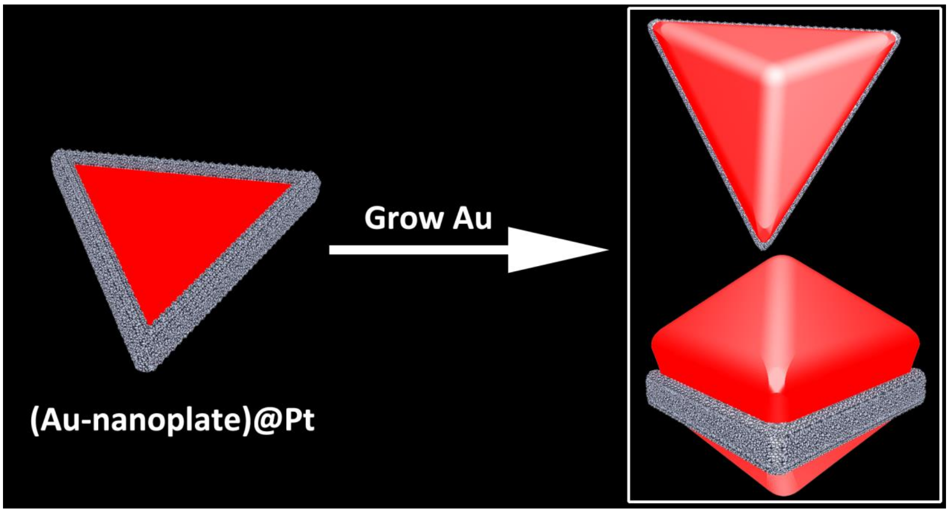


**Supplementary Fig. 82** The schematic overgrowth of Au on the AuNP@PtNC nanocrystal**.**

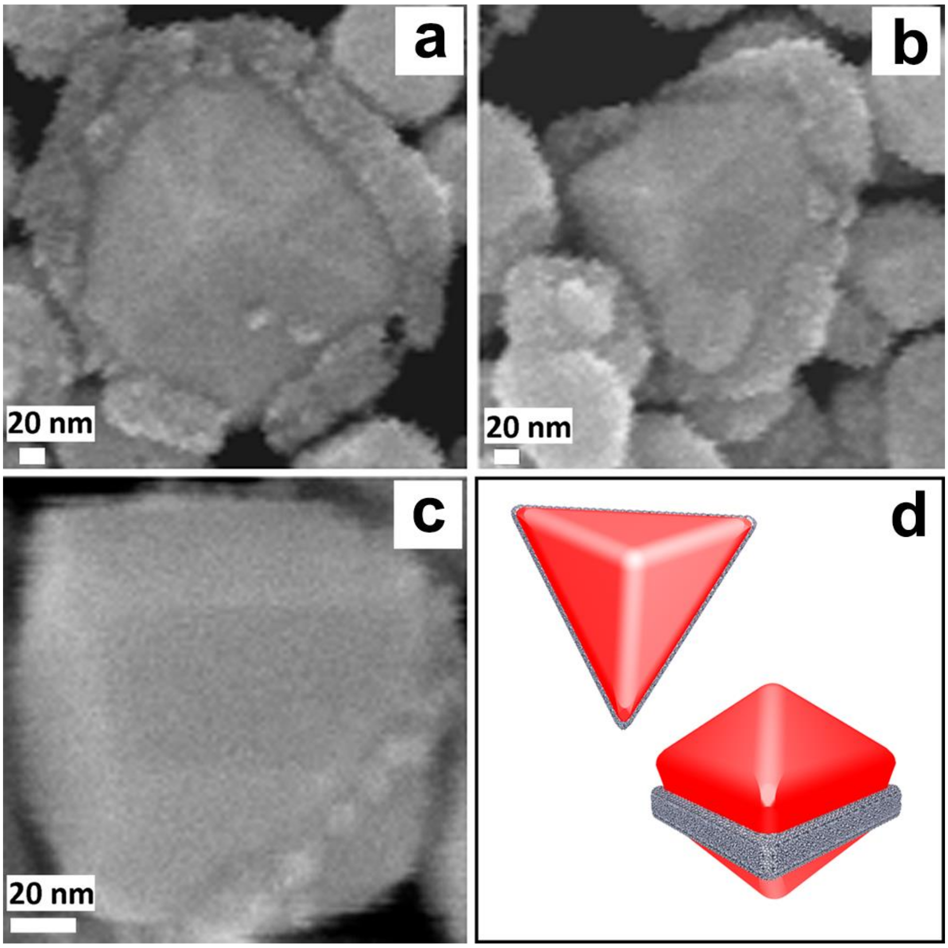


**Supplementary Fig. 83 a**, **b**, **c** SEM images and **d** schematic illustration of nanocrystal prepared through overgrowing Au on AuNP@PtNC seed.

## Supplementary Note 5. Characterizations of high-order complex nanocrystals

This note mainly presents the detailed structure characterizations of high-order complex multi-metal nanocrystals.

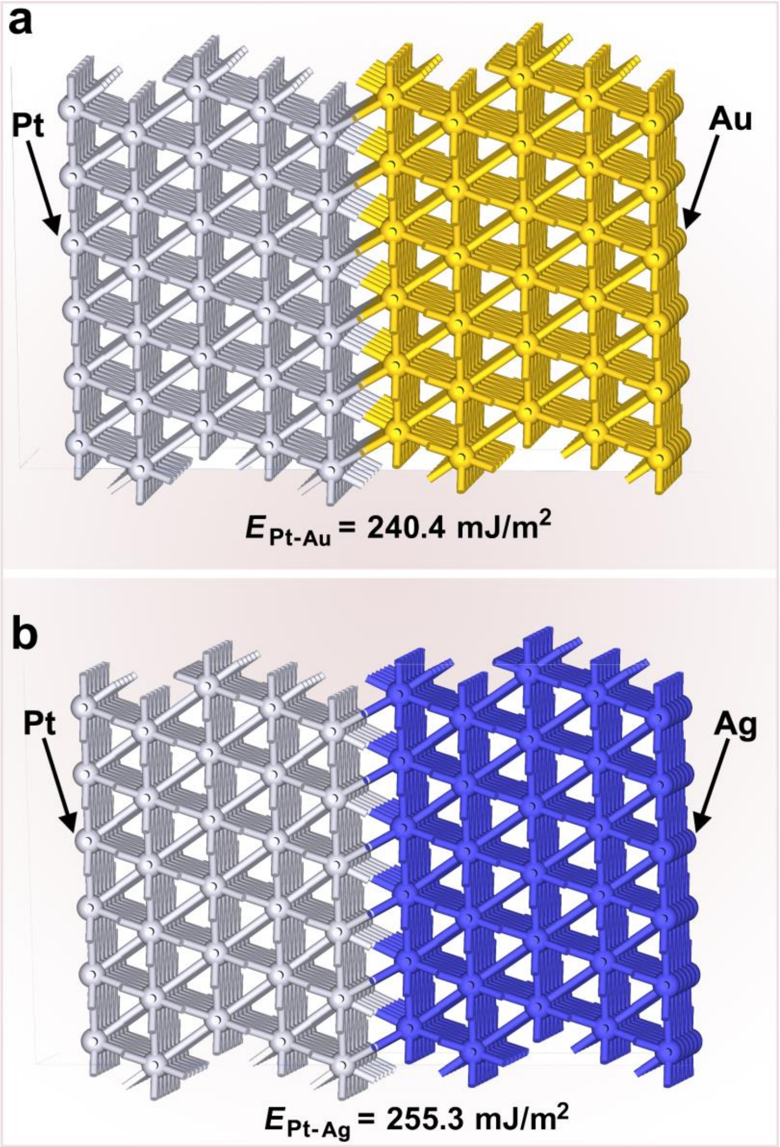


**Supplementary Fig. 84** Schematic of **a** Pt-Au interface and **b** Pt-Ag interface and corresponding interface energies.

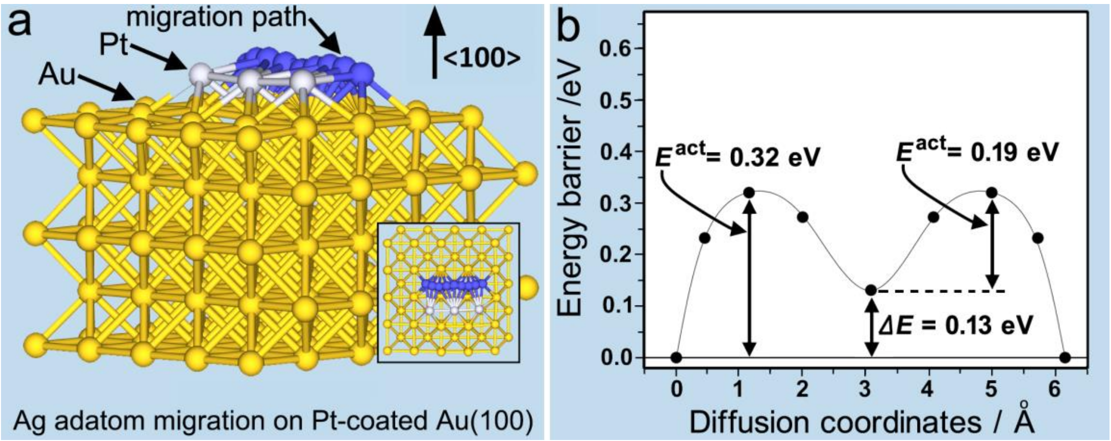


**Supplementary Fig. 85 a** Schematic of Ag adatom migration on Pt-coated Au(100) and **b** DFT study. The inset in **a** is the top view of adsorbate structure.

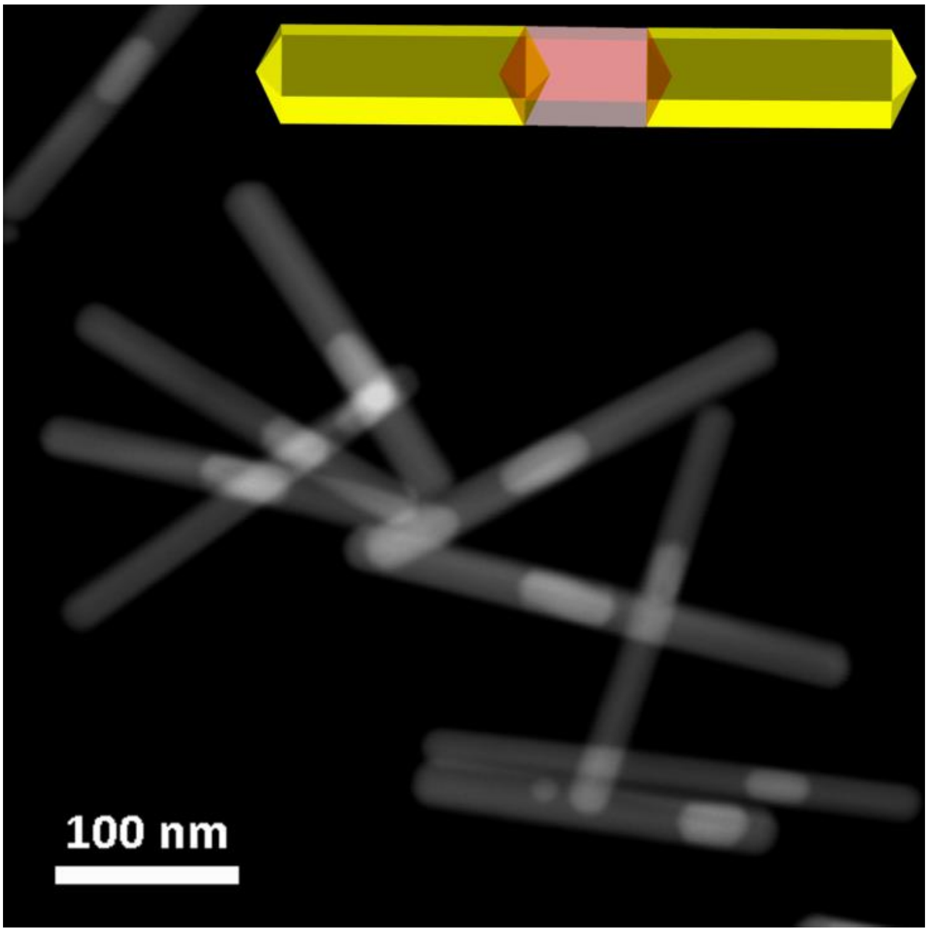


**Supplementary Fig. 86** HAADF image of AgNR-Au(rod)-AgNR multi-segment nanocrystals prepared through overgrowing Ag on AuNRs (Ag/Au=4:1 mol/mol). The inset is the schematic illustration.

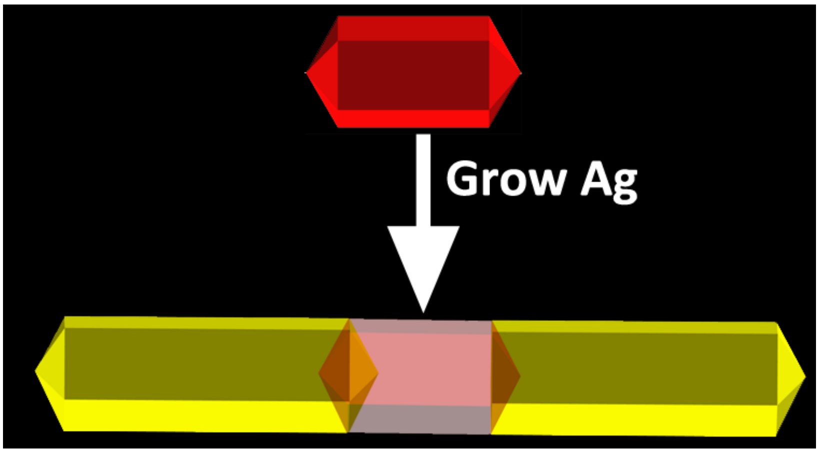


**Supplementary Fig. 87** Schematic illustration of forming AgNR-Au(rod)-AgNR multi-segment nanocrystal shown in Supplementary Fig. 86.

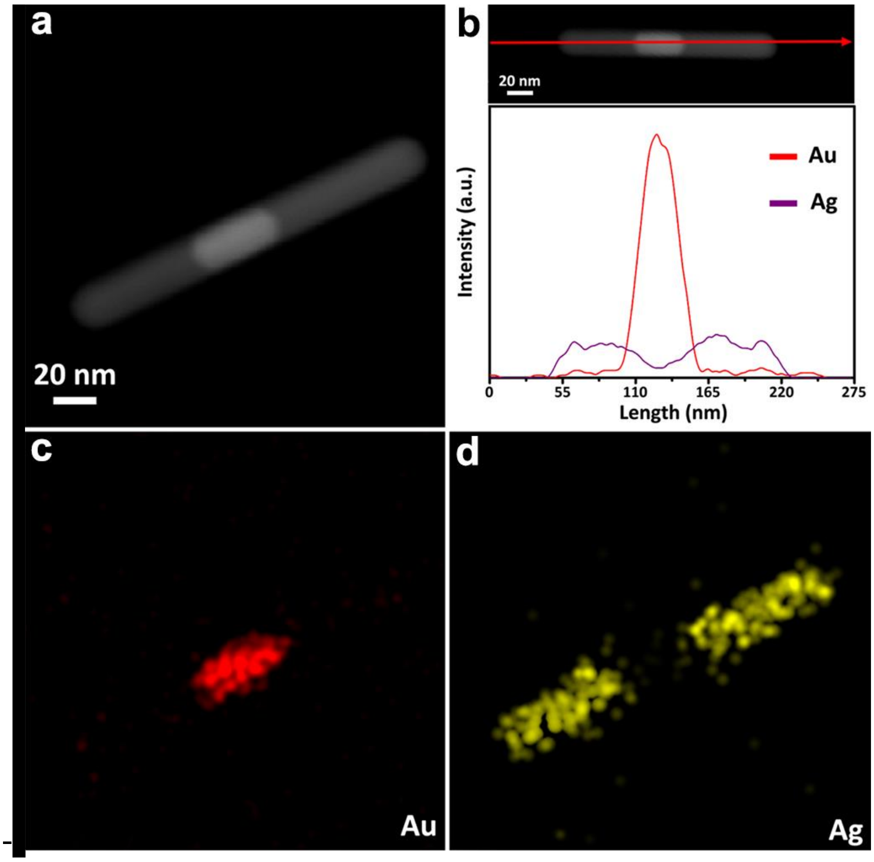


**Supplementary Fig. 88 a** HAADF image, **b** composition line profiles, and **c, d** elemental maps of an individual AgNR-Au(rod)-AgNR multi-segment nanocrystal from products shown in Supplementary Fig. 86. The red arrow in **b** indicates the direction of composition line scanning.

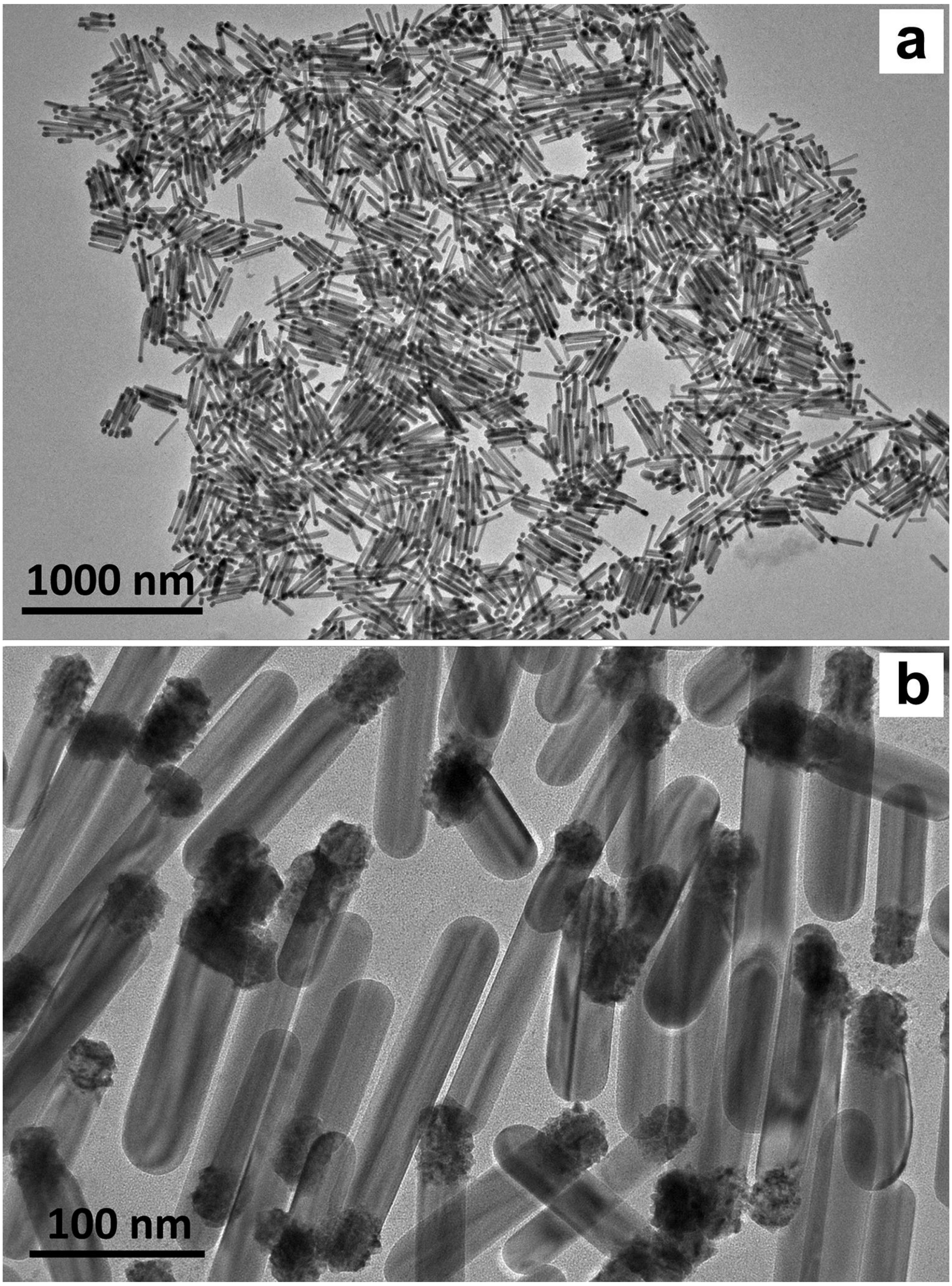


**Supplementary Fig. 89 a**, **b** TEM images of (AuNR@Pt)-AgNR segmented nanocrystals prepared thorough overgrowing Ag on AuNR@Pt seeds shown in Supplementary Fig. 25 (Ag/Au=5:1 mol/mol. Pt/Au=0.3:1 mol/mol). These TEM observations demonstrate the high yield.

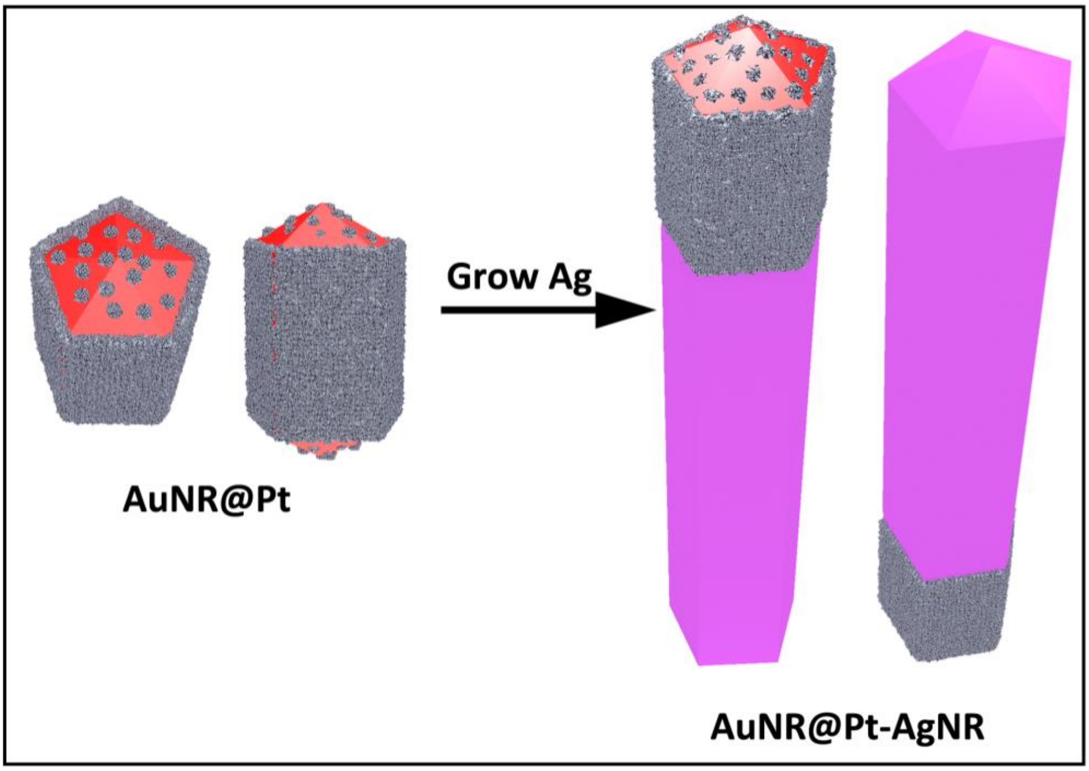


**Supplementary Fig. 90** Schematic illustration for preparing (AuNR@Pt)-AgNR nanocrystals shown in Supplementary Fig. 89.

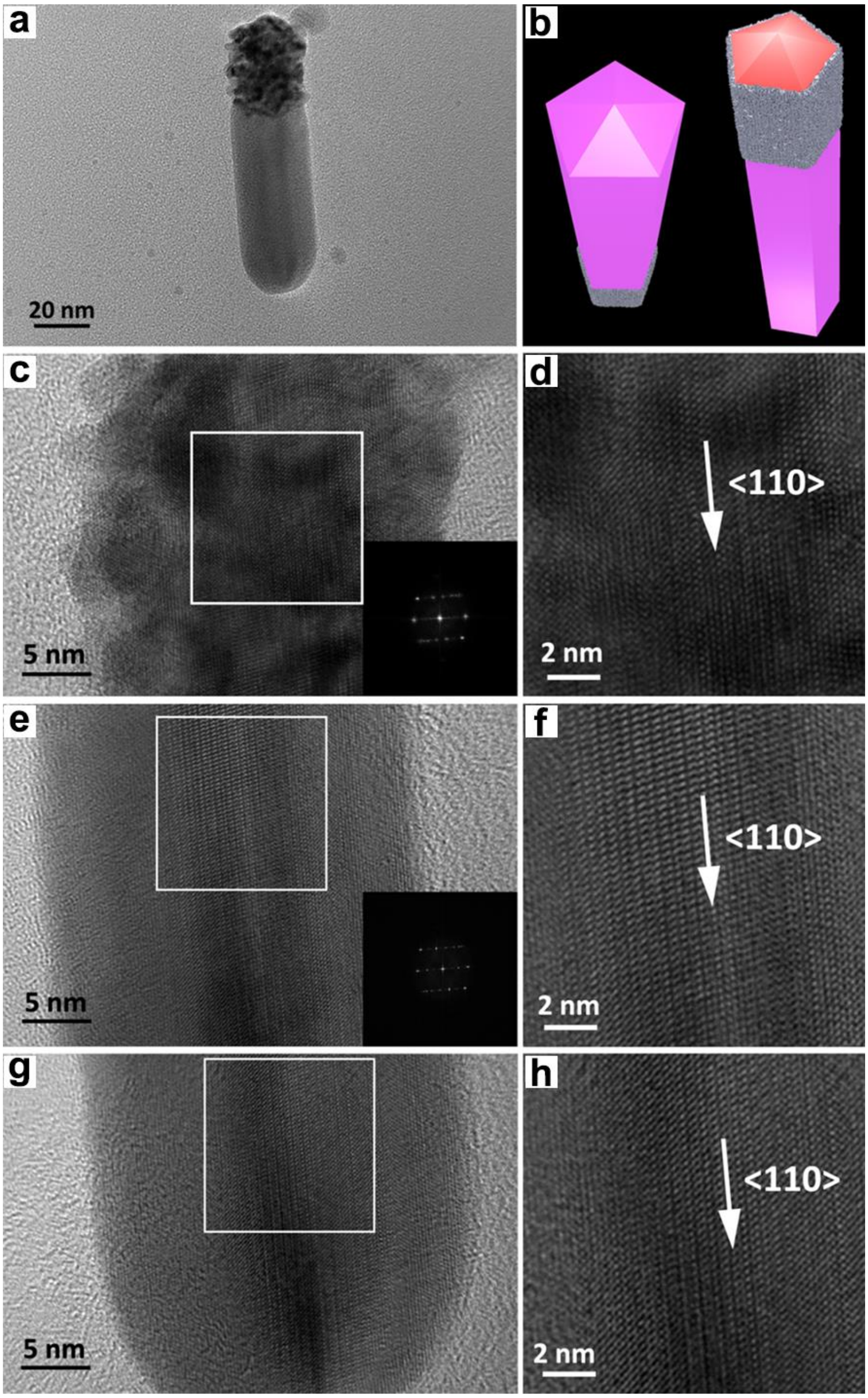


**Supplementary Fig. 91** **a** TEM image, **b** schematic illustration, and **c**, **d**, **e**, **f**, **g**, **h** HRTEM images of an individual (AuNR@Pt)-AgNR segmented nanocrystal from products shown in Supplementary Fig. 89. **d**, **f**, and **h** are the details of areas marked by the white boxes in **c**, **e**, and **g**, respectively. The insets in **c** and **e** are corresponding FFT patterns which demonstrate AgNR domain is five-fold twinned.

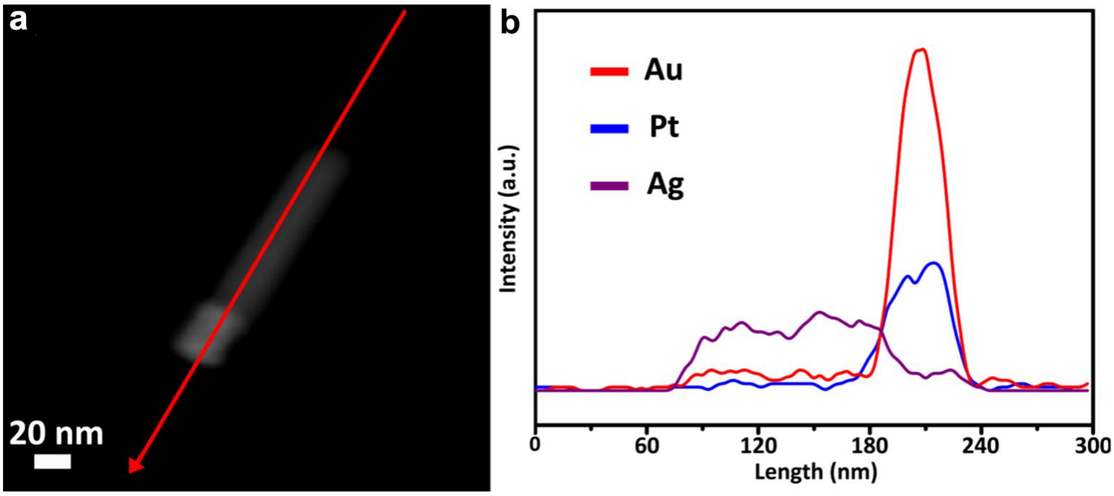


**Supplementary Fig. 92 a** HAADF image and **b** composition line profiles of an individual (AuNR@Pt)-AgNR segmented nanocrystal from products shown in Supplementary Fig. 89. The red arrow in **a** indicates the direction of composition line scanning.

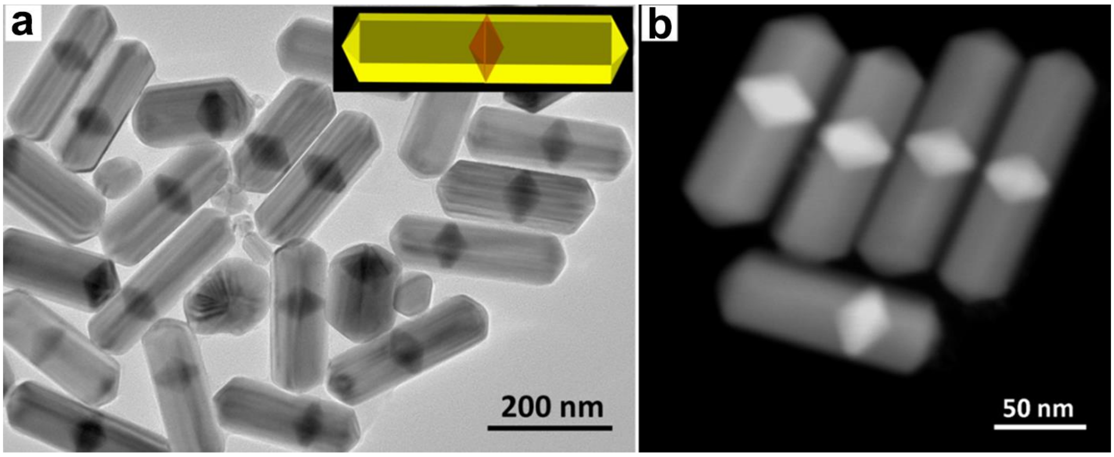


**Supplementary Fig. 93 a** TEM image and **b** HAADF image of AgNR-AuND-AgNR segmented nanocrystals prepared using Pt-free AuNDs as seeds (Ag/Au=6:1 mol/mol). The inset in **a** is the schematic illustration.

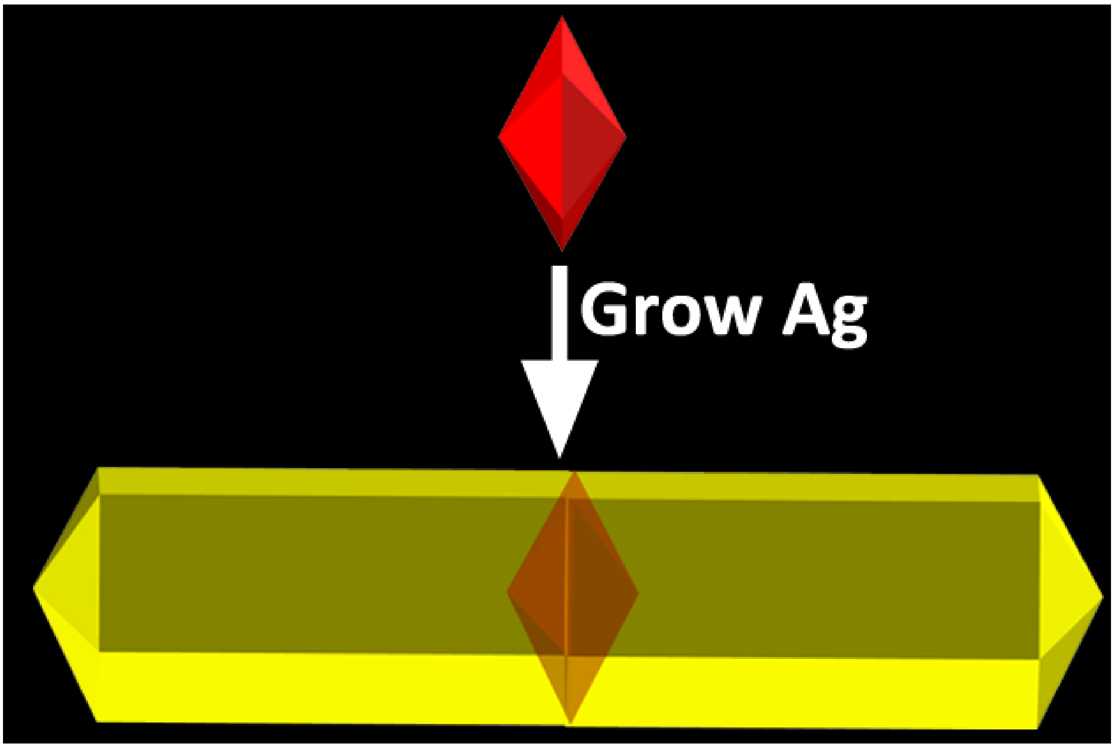


**Supplementary Fig. 94** Schematic illustration of forming AgNR-AuND-AgNR segmented nanocrystal shown in Supplementary Fig. 93.

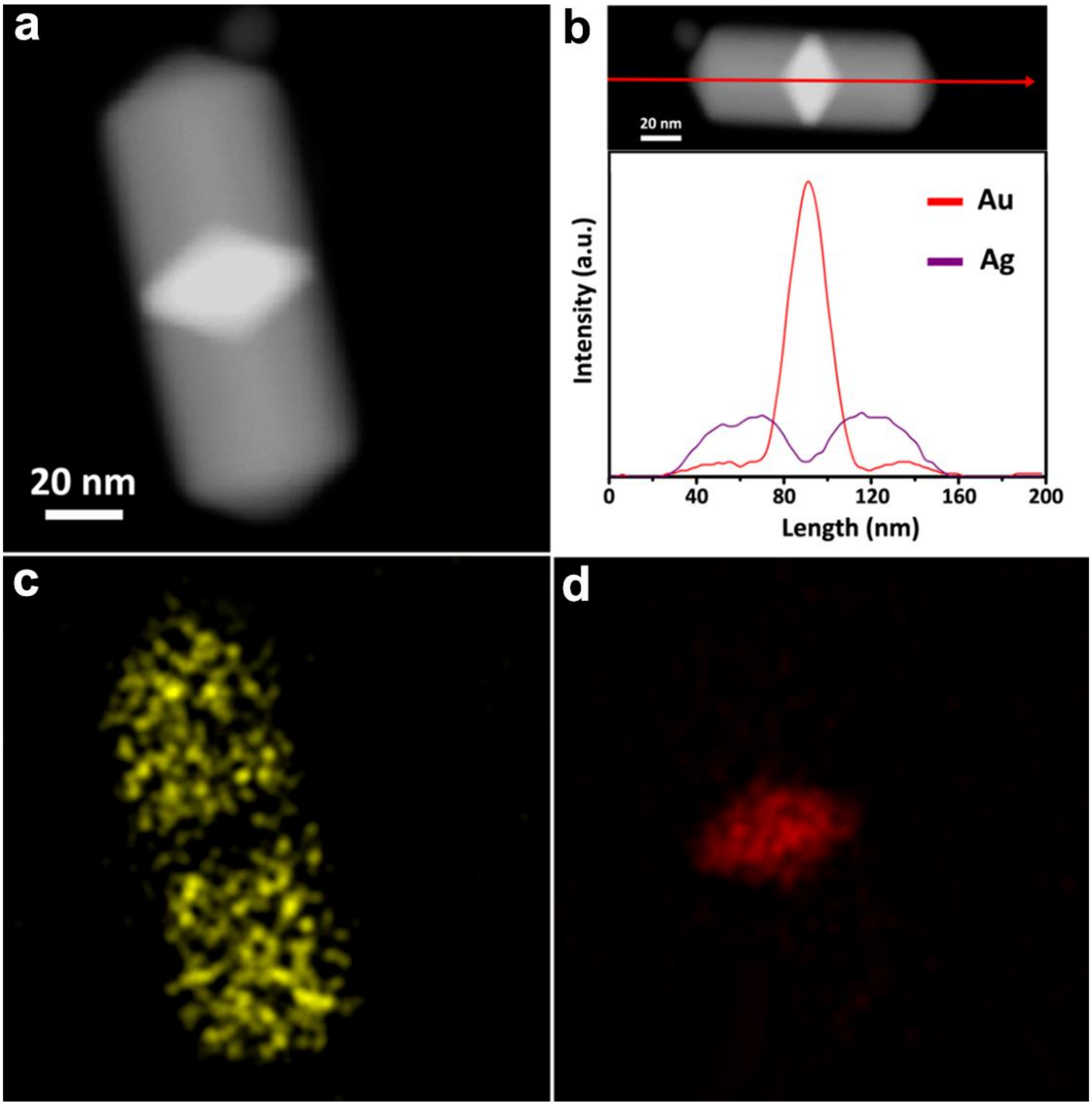


**Supplementary Fig. 95** **a** HAADF image, **b** composition line profiles (The red arrow indicates the scanning direction), and **c**, **d** elemental maps of an individual AgNR-AuND-AgNR segmented nanocrystal from products shown in Supplementary Fig. 93.

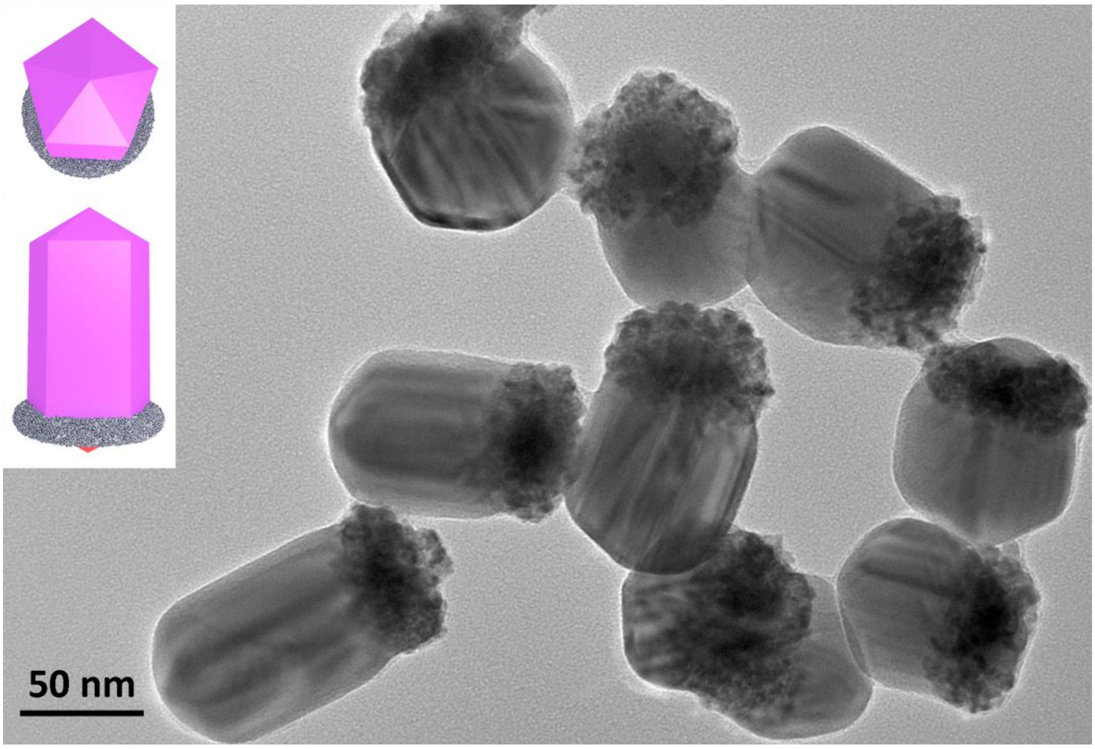


**Supplementary Fig. 96** TEM image of (AuND@PtNC)-AgNRs prepared through overgrowing Ag on AuND@PtNC nanocrystals shown in Supplementary Fig. 1b (Ag/Au=4:1 mol/mol. Pt/Au=1:2 mol/mol). The inset is the schematic illustration.

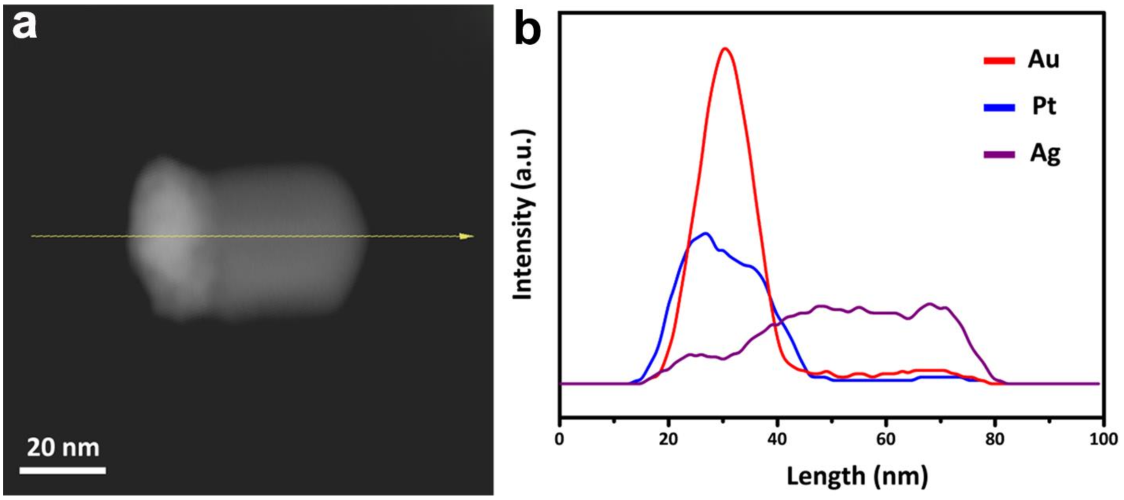


**Supplementary Fig. 97** **a** HAADF image and **b** composition line profiles of an individual (AuND@PtNC)-AgNR from products shown in Supplementary Fig. 96. The yellow arrow in **a** represents the direction of composition line scanning.

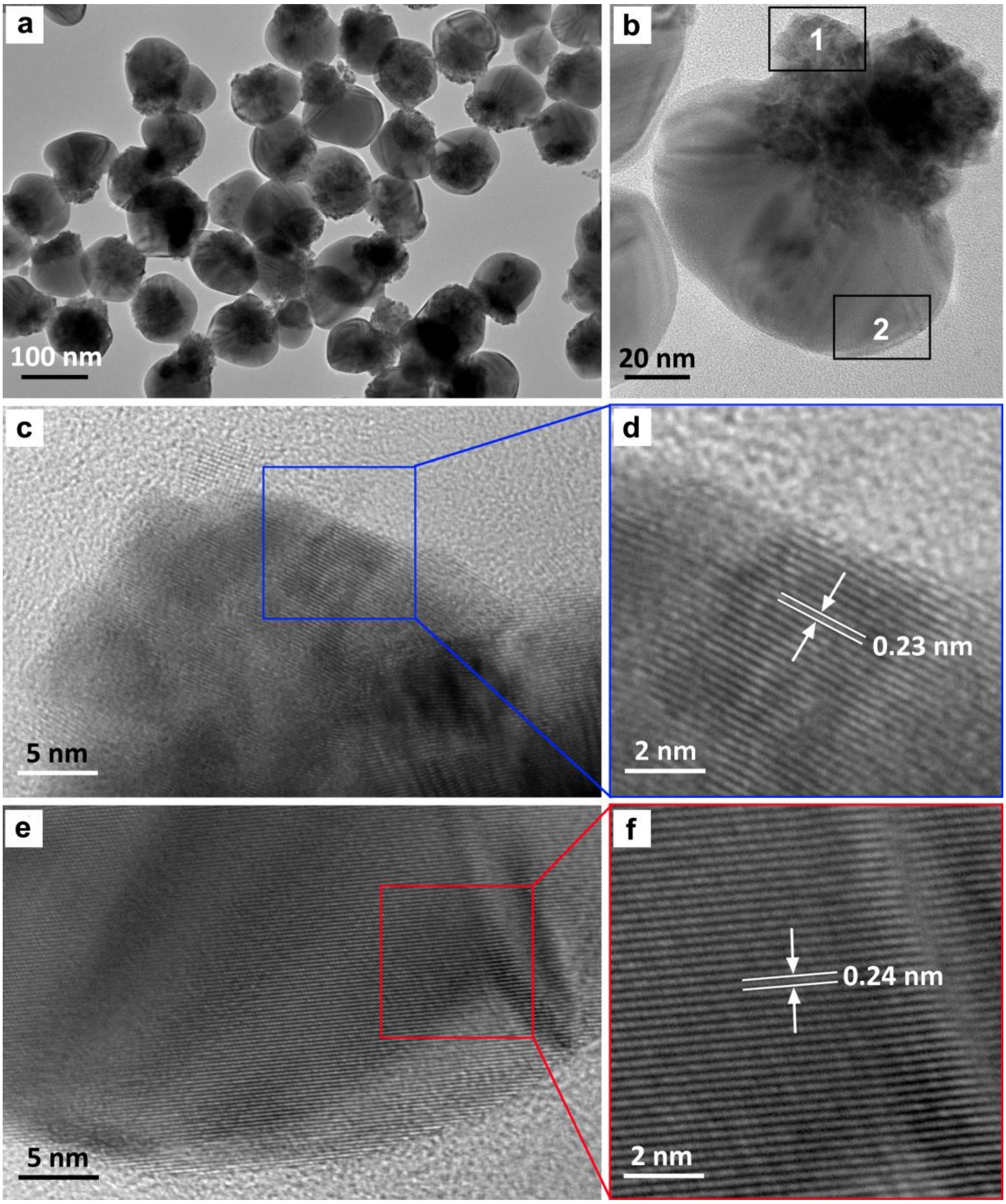


**Supplementary Fig. 98 a** TEM image and **b**, **c**, **d**, **e**, **f** HRTEM images of nanocrystals prepared through overgrowing Ag on AuND@PtNC seeds shown in Supplementary Fig. 1d (Pt/Au=2:1 mol/mol. Ag/Au=6:1 mol/mol). **c** and **e** are the detailed structures of regions 1 and 2 in **b**, respectively. **d** Detailed structures of area marked by the blue box in **c**. **f** Detailed structures of area marked by the red box in **e**. 0.23 nm in **d** and 0.24 nm in **f** are assigned to the interplanar spacing of Pt(111) and Ag(111), respectively.

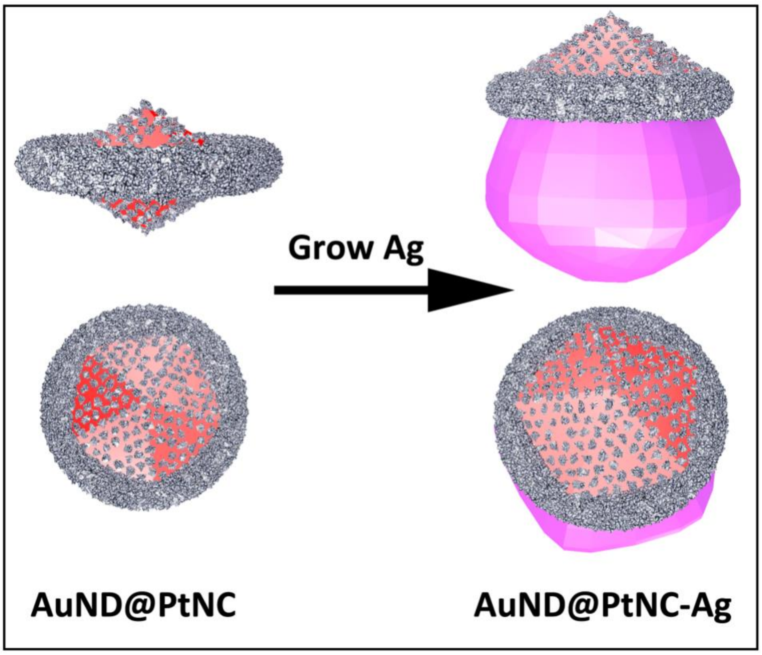


**Supplementary Fig. 99** Schematic illustration for synthesizing nanocrystals shown in Supplementary Fig. 98.

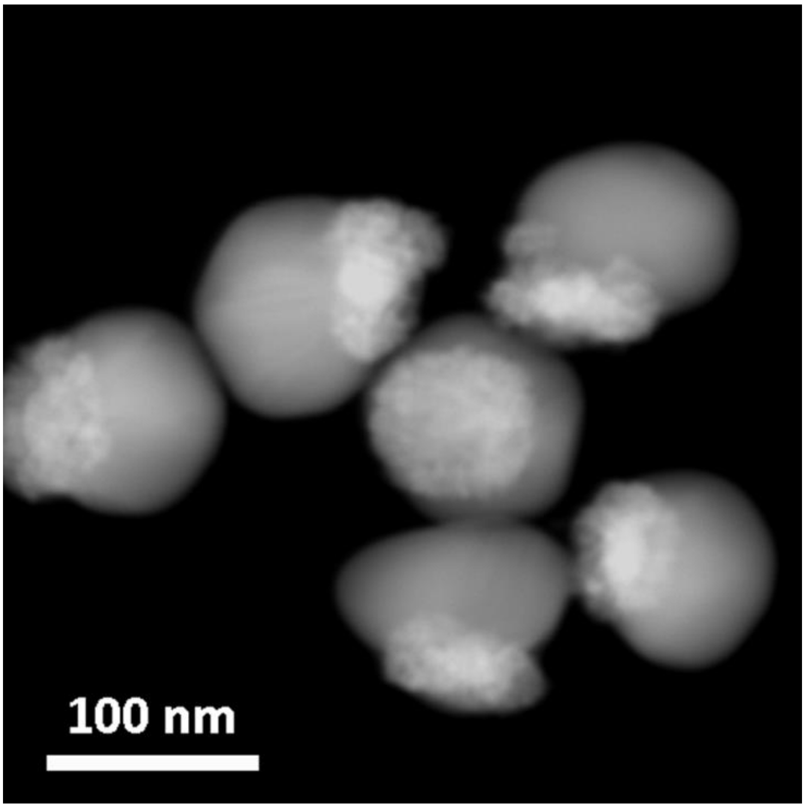


**Supplementary Fig. 100** HAADF image of nanocrystals from product shown in Supplementary Fig. 98.

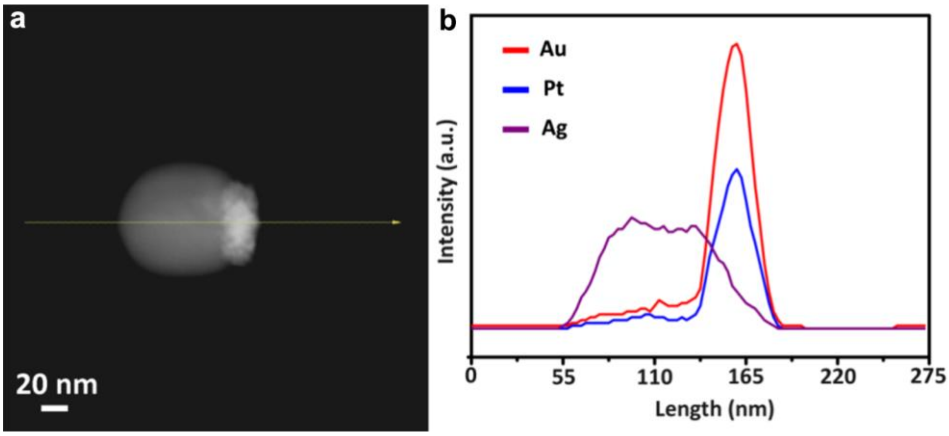


**Supplementary Fig. 101 a** HAADF image and **b** composition line profiles of an individual nanocrystal from products shown in Supplementary Fig. 100. The yellow arrow in **a** represents the direction of composition line scanning.

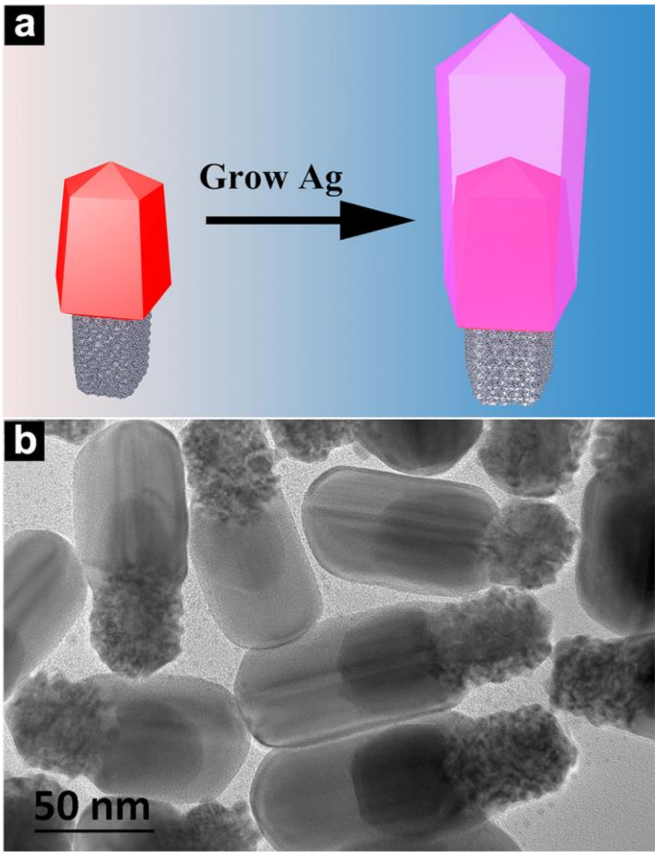


**Supplementary Fig. 102 a** Schematic illustration and **b** TEM image of (AuNR@Pt)-AuNR-AgNR nanocrystals through overgrowing Ag on particles shown in Supplementary Fig. 31. It is difficult to prepare these nanocrystals in high yield because the seeds are not monodispersed.

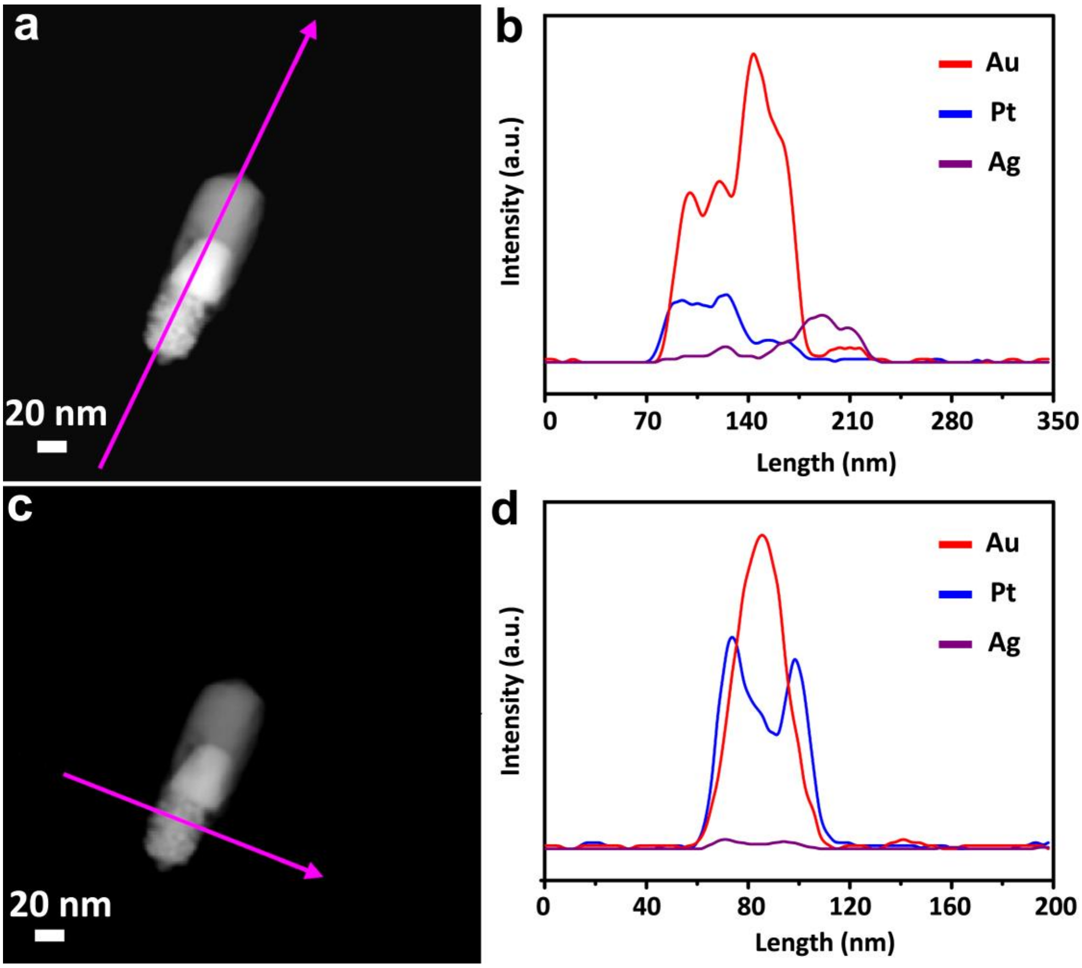


**Supplementary Fig. 103** **a** HAADF image of an individual nanocrystal from products shown in Supplementary Fig. 102 and **b** composition line profiles along the scanning direction marked by the arrow in **a**. **c** HAADF image of the same nanocrystal and **d** composition line profiles along the scanning direction marked by the arrow in **c**.

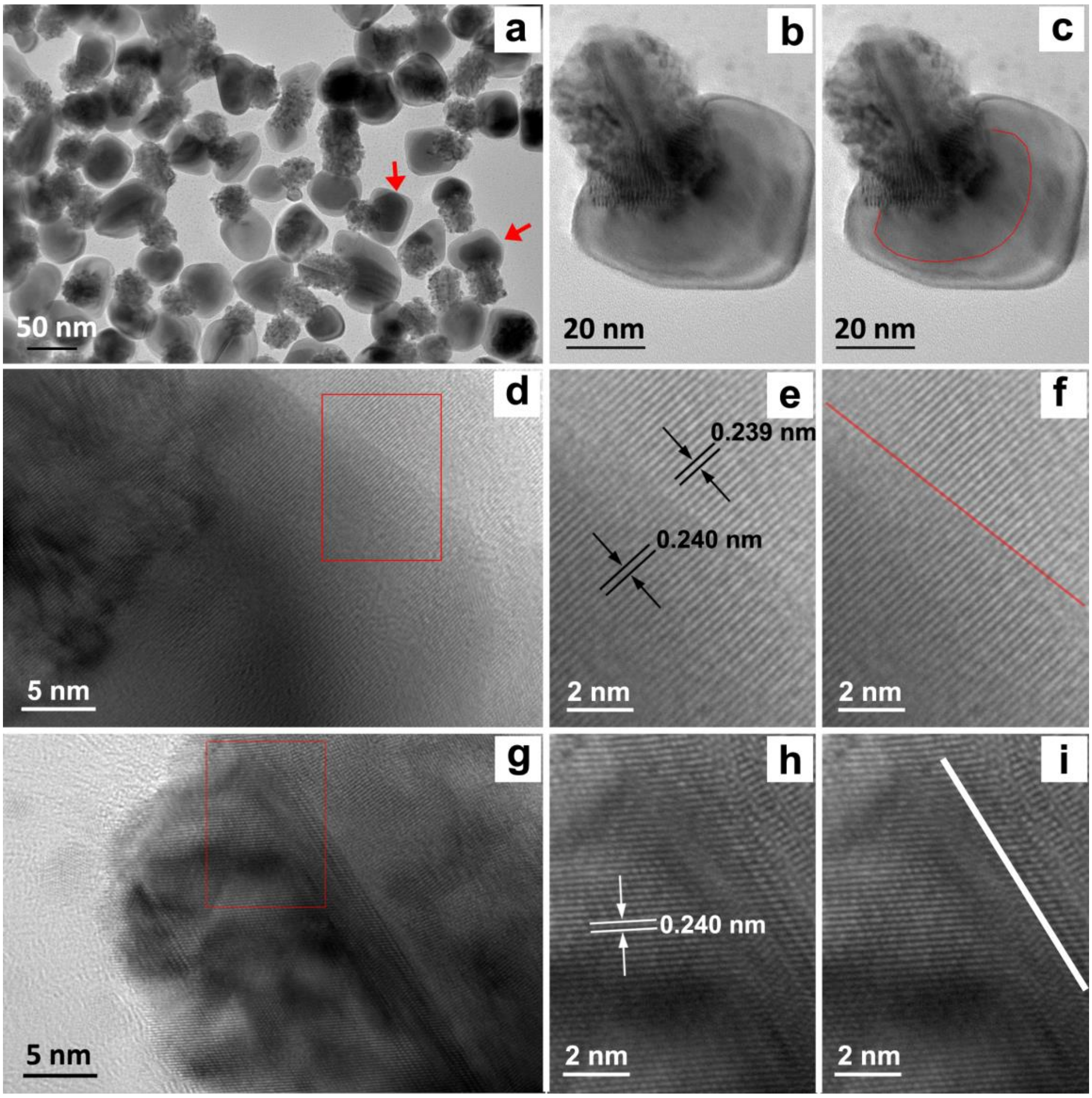


**Supplementary Fig. 104** **a** TEM image and **b, c, d, e, f, g, h, i** HRTEM images of (AuNR@Pt)-(Au@Ag) nanocrystals prepared through overgrowing Ag on nanocrystals shown in Supplementary Fig. 38 (Ag/Au=2:1 mol/mol). **e** and **f** are the details of area marked by the red box in **d**. **h** and **i** are the details of area marked by the red box in **g**. The red line in **f** indicates the Ag-Au interface. The white line in **i** indicates the five-fold axis.

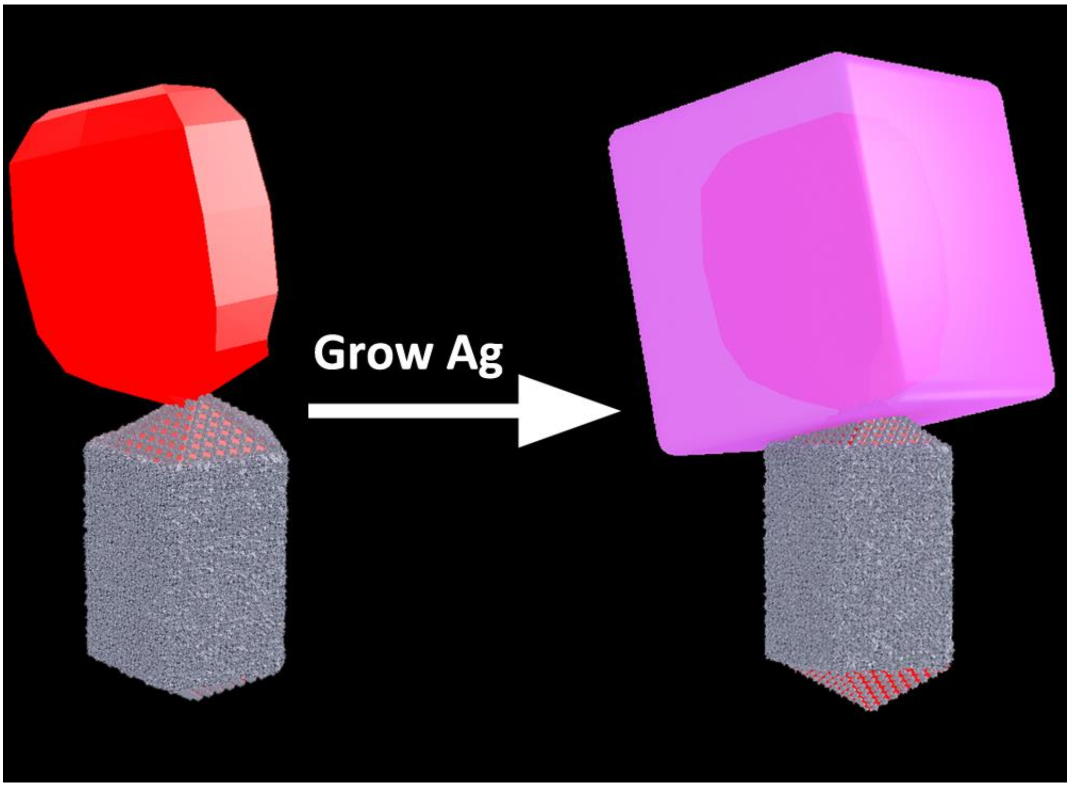


**Supplementary Fig. 105** Schematic illustration of forming (AuNR@Pt)-(Au@Ag) nanocrystals.

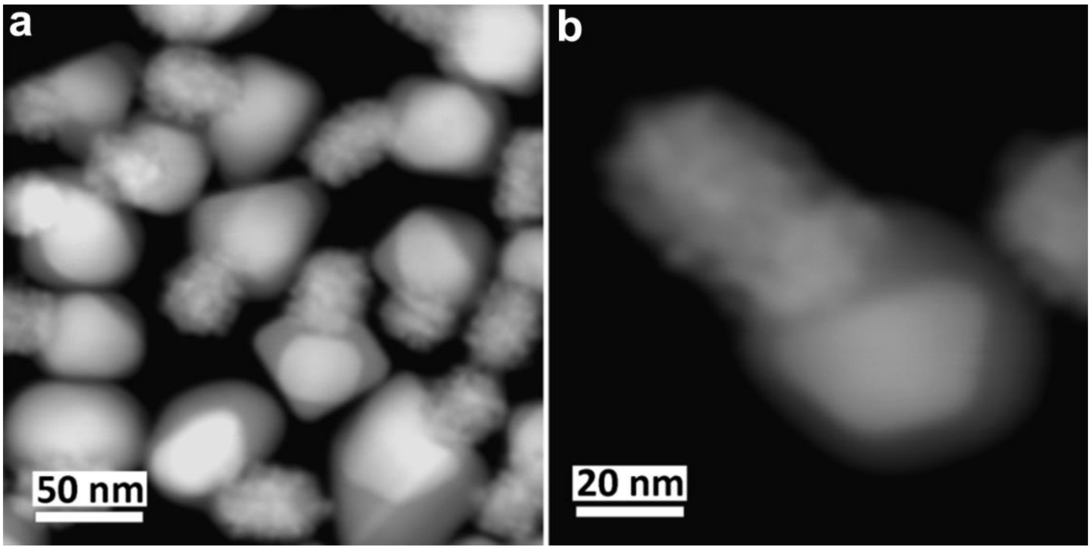


**Supplementary Fig. 106** **a**, **b** HAADF images of (AuNR@Pt)-(Au@Ag) nanocrystals shown in Supplementary Fig. 104.

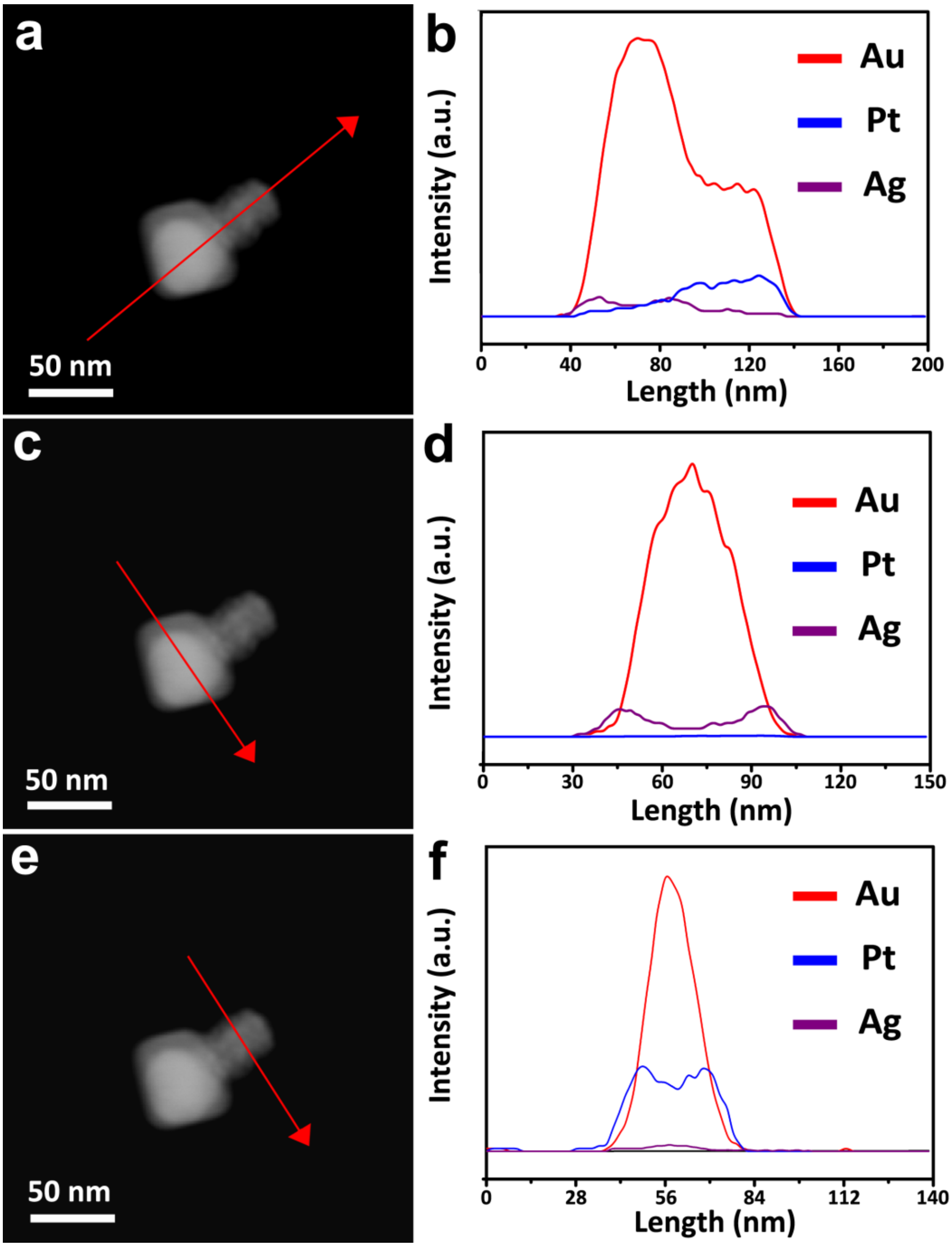


**Supplementary Fig. 107** **a** HAADF image of an individual nanocrystal from products shown in Supplementary Fig. 106 and **b** composition line profiles along the scanning direction marked by the red arrow in **a**. **c** HAADF image of the same nanocrystal and **d** composition line profiles along the scanning direction marked by the red arrow in **c**. **e** HAADF image of the same nanocrystal and **f** composition line profiles along the scanning direction marked by the red arrow in **e**.

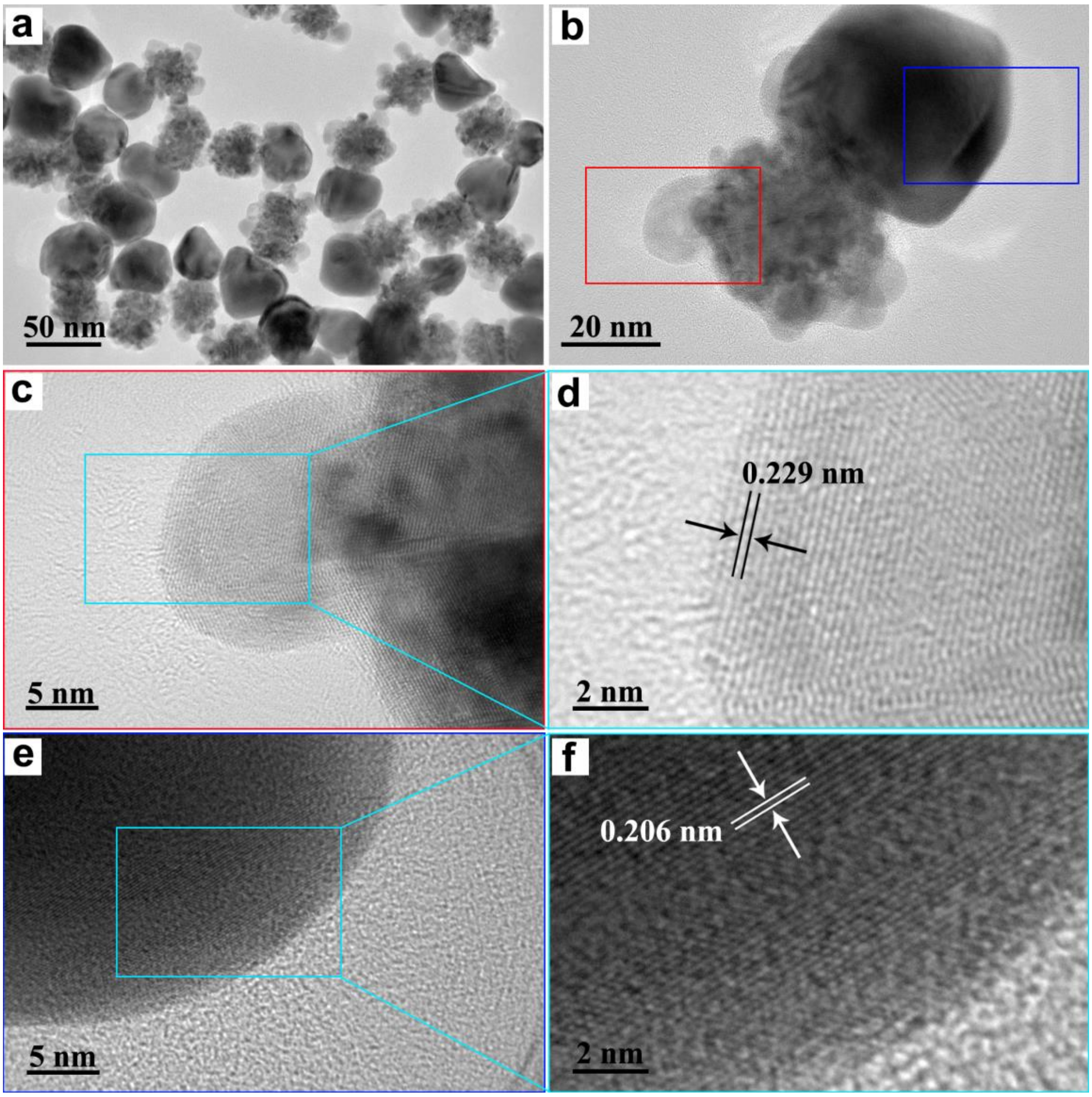


**Supplementary Fig. 108** **a** TEM image and **b**, **c**, **d**, **e**, **f** HRTEM images of [(AuNR@Pt)@Pd]-Au nanocrystals prepared through overgrowing Pd on nanocrystals shown in Supplementary Fig. 38 (Pd/Pt=1:1 mol/mol). **c** and **e** are the details of areas marked by the red and blue boxes in **b**, respectively. **d** and **f** are the details of areas marked by the cyan boxes in **c** and **e**, respectively.

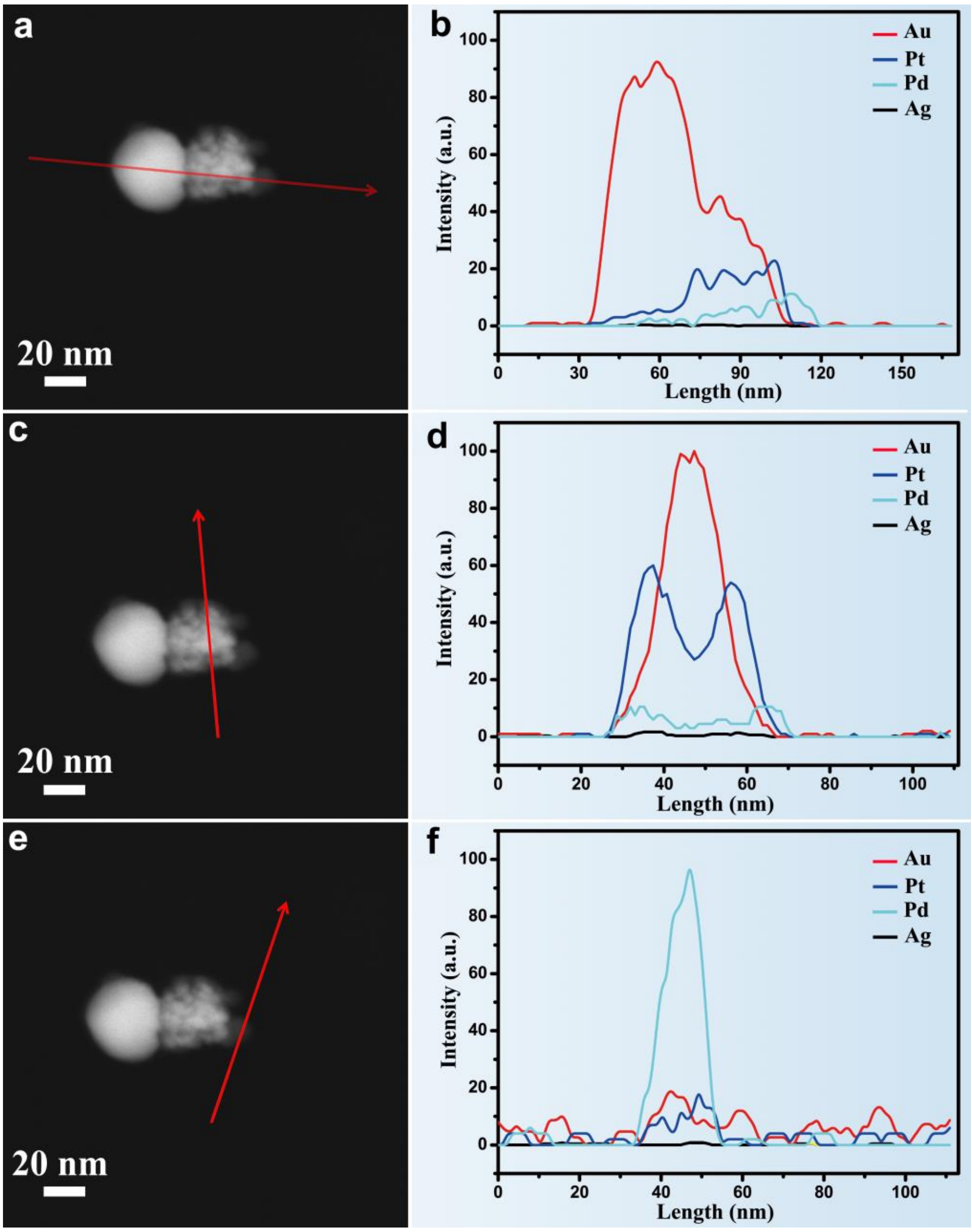


**Supplementary Fig. 109** **a** HAADF image of an individual [(AuNR@Pt)@Pd]-Au nanocrystal from products shown in Supplementary Fig. 108 and **b** composition line profiles along the scanning direction marked by the red arrow in **a**. **c** HAADF image of the same nanocrystal and **d** composition line profiles along the scanning direction marked by the red arrow in **c**. **e** HAADF image of the same nanocrystal and **f** composition line profiles along the scanning direction marked by the red arrow in **e**. These results demonstrate that Pd atoms selectively grow on Pt layer.

## Supplementary Note 6. SERS measurements

This note mainly presents the schematic of liquid cell for SERS measurements, SERS measurement results of various nanocrystals, and structure characterizations of (AuNB-core)@(Pt-shell) nanocrystals in SERS measurement.

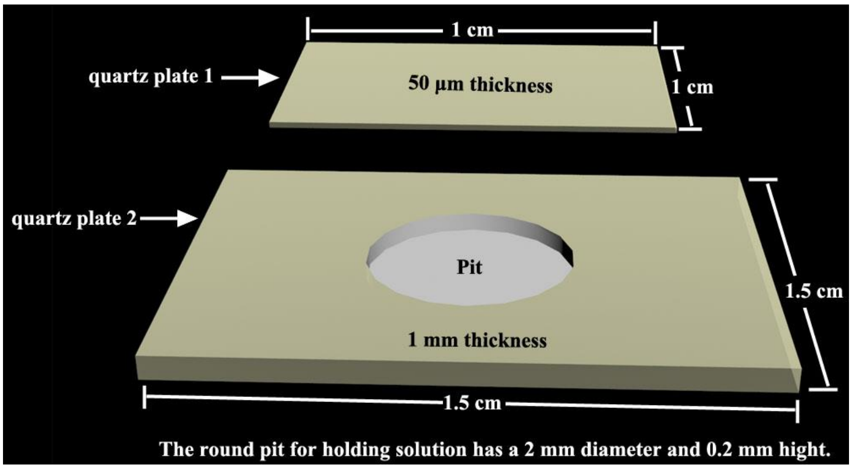


**Supplementary Fig. 110** Schematic illustration of quartz cell for SERS test (Quartz plate 1 is used to cover the pit and quartz plate 2 with pit is used to hold the tested solution).

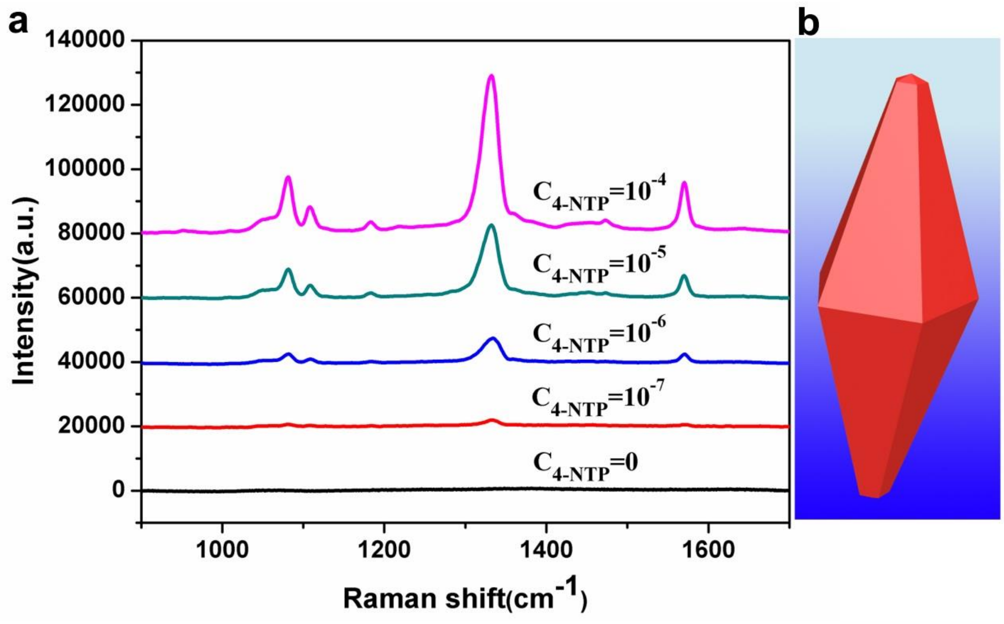


**Supplementary Fig. 111 a** Raman spectra of various concentrations of 4-NTP on AuNB nanocrystals shown in Supplementary Fig. 10a. **b** Schematic illustration of AuNB nanocrystal. Source data are provided as a Source Data file.

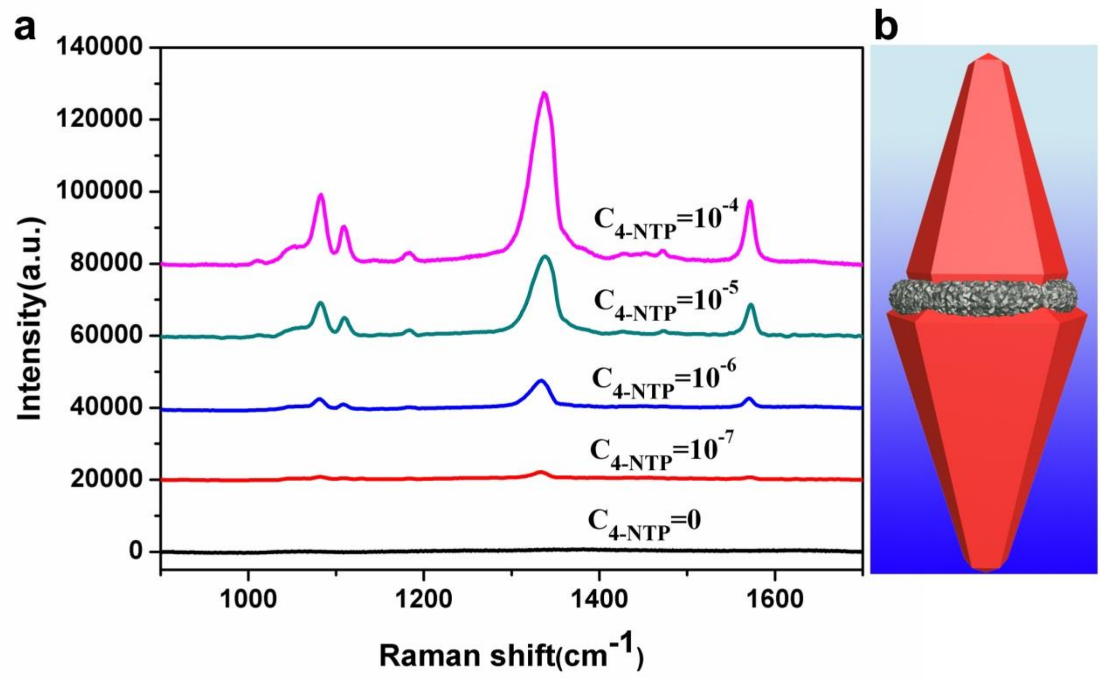


**Supplementary Fig. 112 a** Raman spectra of various concentrations of 4-NTP on AuNB@PtNC nanocrystals shown in Supplementary Fig. 10e**. b** Schematic illustration of AuNB@PtNC nanocrystal. Source data are provided as a Source Data file.

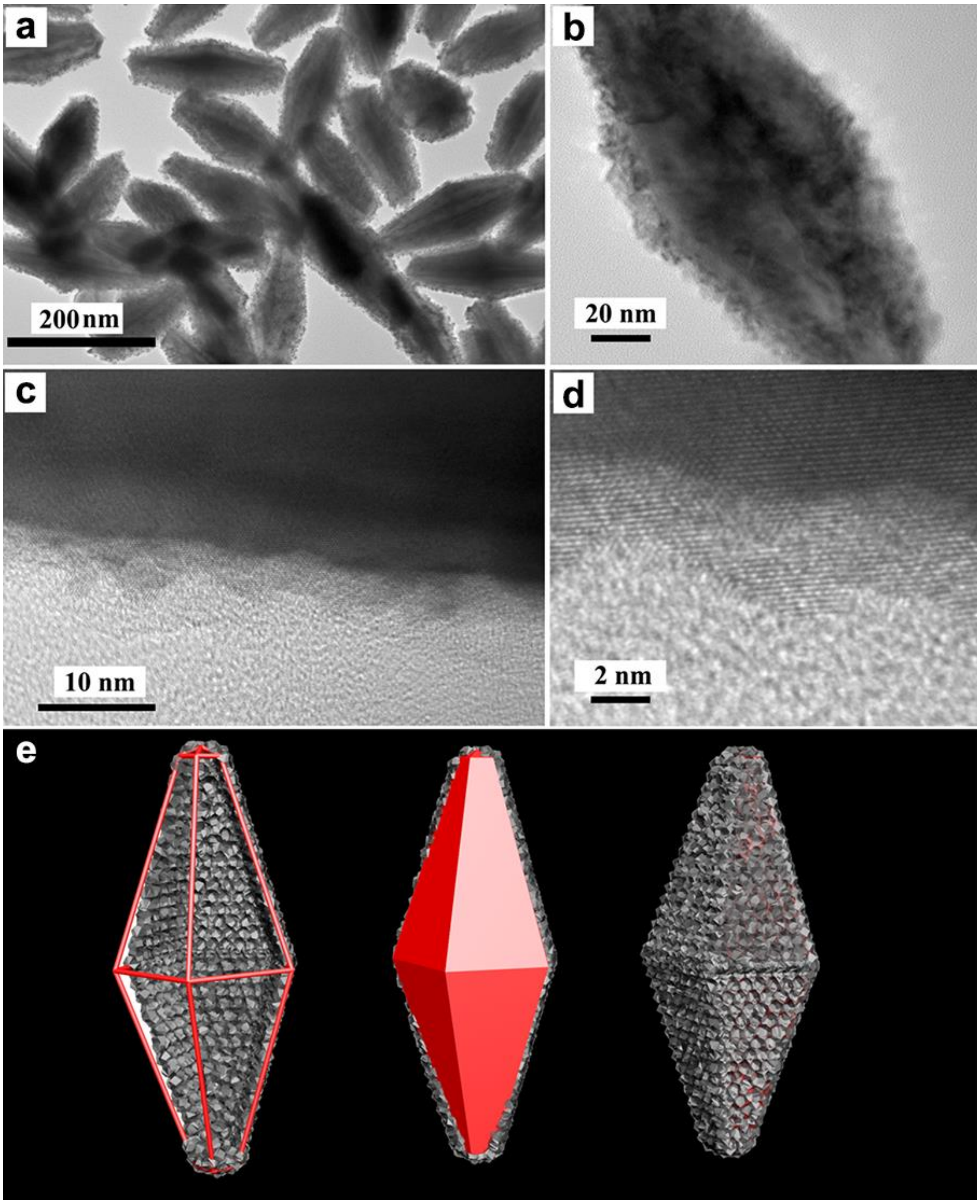


**Supplementary Fig. 113 a** TEM image, **b**, **c**, **d** HRTEM images, and **e** schematic illustrations of (AuNB-core)@(Pt-shell) nanocrystals prepared with conventional synthetic process in aqueous CTAB solution (Pt/Au=1:2 mol/mol). The HRTEM images in **c** and **d** show that the thickness of island-like Pt layer is close to 2.6 nm, and the FDTD model was built based this HRTEM result.

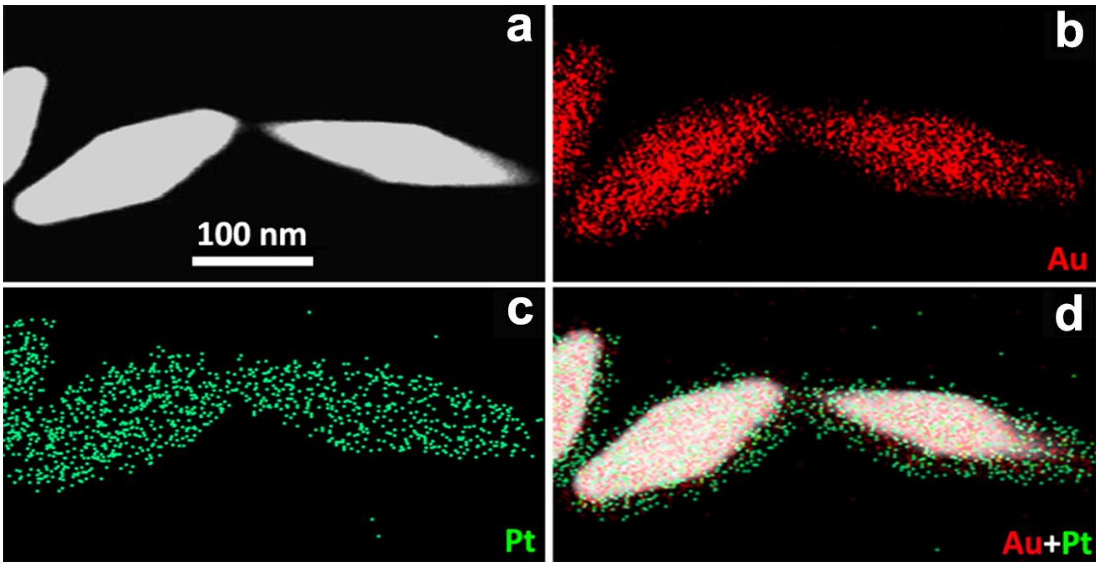


**Supplementary Fig. 114 a** HAADF image and **b**, **c**, **d** element maps of (AuNB-core)@(Pt-shell) nanocrystals from products shown in Supplementary Fig. 113.

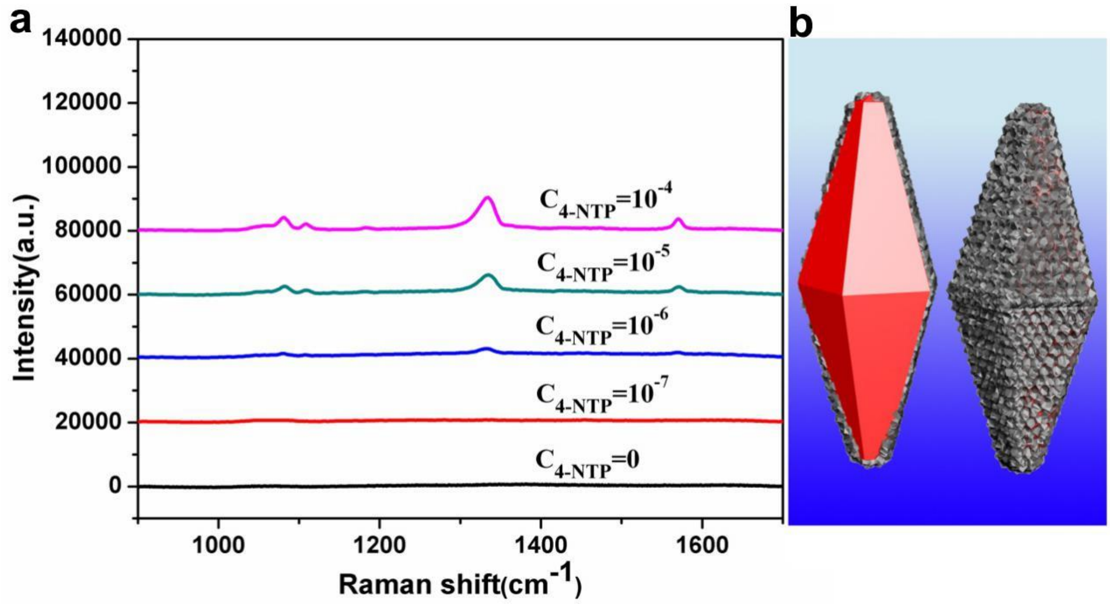


**Supplementary Fig. 115 a** Raman spectra of various concentrations of 4-NTP on (AuNB-core)@(Pt-shell) nanocrystals shown in Supplementary Fig. 113. **b** Schematic illustration of (AuNB-core)@(Pt-shell) nanocrystal. Source data are provided as a Source Data file.

## Supplementary References


1. Li, X. et al. The unusual effect of $AgNO_3$ on the growth of Au nanostructures and their catalytic performance. *Nanoscale* **5**, 4976−4985(2013).
2. Han, S. et al., The alloying effect and AgCl-directing growth for synthesizing a trimetallic nanoring with improved SERS. *Nanoscale* **7**, 20414-20425 (2015).
3. Johnson, P. B. & Christy, R. W. Optical constants of the noble metals. *Phys. Rev. B* **6**, 4370-4379 (1972).
4. Kresse, G. & Furthmüller, J. Efficient iterative schemes for ab initio total-energy calculations using a plane-wave basis set. *Phy. Rew. B* **54**, 11169–11186 (1996).
5. Perdew, J. P. Burke, K. & Ernzerhof, M. Generalized gradient approximation made simple. *Phys. Rev. Lett.* **77**, 3865–3868 (1996).
6. Blöhl, P. E. Projector augmented-wave method. *Phys. Rev. B* **50**, 17953–17979 (1994).
7. Perdew, J. P. et al. Restoring the density-gradient expansion for exchange in solids and surfaces. *Phys. Rev. Lett.* **100**, 136406 (2008).
8. Pack, J. D. & Monkhorst, H. J. Special points for Brillouin-zone integrations"-a reply. *Phys. Rev. B* **16**, 1748–1749 (1977).
9. Henkelman, G., Uberuaga, B. P. & Jónsson, H. A climbing image nudged elastic band method for finding saddle points and minimum energy paths. *J. Chem. Phys.* **113**, 9901–9904 (2000).
10. García-Negrete, C. A., Rojas, T. C., Knappett, B. R., Jefferson, D. A. Wheatley, A. E. H. & Fernández, A. Shape-defined nanodimers by tailored heterometallic epitaxy. *Nanoscale* **6**, 11090-11097 (2014).
11. Jang, H. J. et al. Fabrication of 2D Au nanorings with Pt framework. *J. Am. Chem. Soc*. **136**, 17674−17680 (2014).
